# Comparative clinical efficacy of acupuncture-related therapies for ulcerative colitis: a systematic review and network meta-analysis

**DOI:** 10.3389/fmed.2025.1676608

**Published:** 2025-12-12

**Authors:** Longtao Zhang, Yanxiao Sun, Ziyang Ru, Dan Li, Yuxia Ma

**Affiliations:** 1 Acupuncture and Tuina College of Shandong University of Traditional Chinese Medicine, Jinan, Shandong, China; 2 The First Clinical Medical College of Shandong University of Traditional Chinese Medicine, Jinan, Shandong, China; 3 Key Laboratory of Traditional Chinese Medicine Classical Theory, Ministry of Education, Shandong University of Traditional Chinese Medicine, Shandong, Jinan, China

**Keywords:** ulcerative colitis, acupuncture-related therapies, network meta-analysis, randomized controlled trials, systematic review

## Abstract

**Background:**

Ulcerative colitis (UC) is a chronic and recurrent inflammatory bowel disease. Current drug treatments are often associated with side effects, unstable efficacy, and high relapse rates. Therefore, exploring complementary and alternative therapies such as acupuncture is of significant importance for optimizing UC clinical management strategies. This study employs a network meta-analysis method to systematically compare the efficacy and safety of various acupuncture-related therapies in treating UC, aiming to provide evidence-based guidance for selecting the optimal clinical intervention.

**Methods:**

We systematically searched 8 databases for randomized controlled trials (RCTs) of acupuncture-related therapies for ulcerative colitis, including China National Knowledge Infrastructure (CNKI), Wanfang Database, VIP Database, China Biological Medicine (CBM), PubMed, EMbase, the Cochrane Library, and Web of Science. The total effective rate, Mayo score, Baron endoscopic score were used for primary outcomes, interleukin-6 (IL-6) levels, tumor necrosis factor-alpha (TNF-α) levels, and relapse rate were selected as secondary outcomes. The Cochrane risk of bias tool (RoB 2.0) was used to assess the quality of the articles, and StataMP 18 was used for statistical analysis. Heterogeneity and consistency were assessed, and the comparative effectiveness of different acupuncture interventions was ranked using Surface under the cumulative ranking curve (SUCRA).

**Results:**

A total of 76 RCTs were included, involving 7,484 participants. Auricular acupressure combined with Chinese herbal medicine (AA + CHM) (RR 1.71, 95% CI: 1.47, 1.99, SUCRA = 99.9%) was shown to have effective to improving total effective rate. For Mayo score, the most effective intervention was acupuncture combination therapy (ACU-CT) (SMD –4.85, 95% CI: –6.66, –3.05, SUCRA = 97.5%). In terms of reducing Baron endoscopic score, ACU-CT (SMD-2.31, 95% CI: –3.81, –0.81, SUCRA = 84.1%) had the best efficacy. For IL-6 levels, warm acupuncture (WA) (SMD –3.10, 95% CI: –4.56, –1.65, SUCRA = 96.1%) showed the best efficacy. For TNF-α levels, warm acupuncture combination therapy (WA-CT) (SMD –2.32, 95% CI: –4.54, –0.10, SUCRA = 76.8%) demonstrated the best efficacy. For recurrence rate, Acupuncture (ACU) (OR 0.15, 95% CI: 0.03, 0.65, SUCRA = 89.3%) achieved the greatest reductions. SUCRA analysis showed that WA, ACU-CT, WA-CT, and acupoint catgut embedding (ACE) ranked highest in most of the outcomes. Acupoint frequency statistics revealed that the most commonly used acupoints for treating UC were Tianshu (ST25), Zusanli (ST36), Shangjuxu (ST37), Zhongwan (CV12), Guanyuan (CV4), Dachangshu (BL25), Pishu (BL20), Shenque (CV8), Qihai (CV6), and Sanyinjiao (SP6).

**Conclusion:**

These findings may provide preliminary evidence-based guidance for acupuncture-related interventions as a potential complementary or alternative treatment for patients with UC, particularly those who have poor responses to conventional pharmaceutical treatment. In the future, more large-sample, high-quality RCTs are needed to further confirm the long-term efficacy and mechanisms of different acupuncture interventions.

**Systematic review registration:**

https://www.crd.york.ac.uk/prospero/, identifier CRD420251082924.

## 1 Introduction

Ulcerative colitis (UC) is a chronic immune-mediated inflammatory bowel disease (IBD) characterized by persistent inflammation of the colonic and rectal mucosa, often manifests clinically with diarrhea, abdominal pain, bloodymucopurulent, and tenesmus (1, 2). The global prevalence of UC reached 5 million cases in 2023 (2). With changes in the environment, dietary structure, and lifestyle, the incidence and prevalence of UC have been rising annually, particularly in developed countries. With the increasing disease burden, this trend has been extended to traditionally low-incidence regions that are undergoing industrialization (3, 4). Data indicate that UC patients face a 4.5% risk of developing colorectal cancer within 20 years of diagnosis (5), which is 2–3 times higher than that of the general population (6). Furthermore, UC not only causes gastrointestinal symptoms but is also frequently associated with chronic pain, anxiety, and depression, significantly reducing quality of life (7).

Traditional treatments include 5-aminosalicylic acid (5-ASA), corticosteroids, immunomodulators, and biologics (8, 9). While these medications can control inflammation in the short term, they are associated with significant side effects, unstable efficacy, high relapse rates, and drug dependence. For example, 5-ASA may cause gastrointestinal symptoms such as headaches and nausea, and in severe cases, it can lead to interstitial nephritis and other nephrotoxic reactions; Common side effects of corticosteroids include hyperglycemia, osteoporosis, and an increased risk of infection; Immunomodulators are associated with potential adverse effects such as myelosuppression and hepatotoxicity; Biologics carry risks of serious adverse effects, such as serious infections, reactivation of tuberculosis, and lymphoma (10–12). Long-term medication poses safety risks and increases the financial burden on patients. These limitations underscore the pressing need for improved and more effective therapeutic strategies in the management of ulcerative colitis.

Given the limitations of conventional pharmacotherapy, there is a pressing clinical need to explore complementary and alternative treatment options for UC. Acupuncture, a cornerstone of Traditional Chinese Medicine (TCM), was selected for this network meta-analysis for several compelling reasons. First, from a TCM theoretical perspective, the clinical manifestations of UC closely align with syndromes such as “dysentery” and “abdominal pain,” which are traditionally attributed to damp-heat, spleen-kidney deficiency, and qi stagnation. Acupuncture, through stimulation of specific acupoints, is believed to regulate the flow of Qi and Blood, clear damp-heat, and strengthen the spleen and kidney, thereby addressing the fundamental TCM pathological patterns underlying UC.

Furthermore, acupuncture has attracted increasing attention due to its favorable safety profile and multi-target regulatory potential. Clinical studies have shown that acupuncture can alleviate UC symptoms, improve intestinal function, reduce inflammation, and enhance quality of life (13–15). Multiple studies have confirmed that acupuncture therapy can improve the intestinal mucosal microenvironment by modulating the balance of pro-inflammatory and anti-inflammatory factors such as tumor necrosis factor-alpha (TNF-α) and interleukin-6 (IL-6) (16). It can also stimulate specific acupoints to modulate the balance of gut microbiota and regulate gut-brain axis function (17). Furthermore, acupuncture has been shown to inhibit fibrosis and promote epithelial regeneration, as well as to regulate gut microbiota balance (18). Moxibustion can enhance mucosal immune function and promote tissue repair (19). Therefore, a systematic comparison of the relative effectiveness of different acupuncture-related modalities is highly relevant for informing clinical practice and guiding future research.

A diverse range of acupuncture-related therapies is commonly used in clinical practice for UC, primarily including the following modalities: Acupuncture involves the insertion of fine, sterile needles into specific acupoints. Warm acupuncture integrates needle insertion with the burning of moxa on the needle handle to deliver combined needling and thermal stimulation. Electroacupuncture applies a mild electric current through inserted needles to provide continuous stimulation. Moxibustion stimulates acupoints primarily via thermal energy generated by burning moxa (a therapeutic material derived from the dried wool of the mugwort plant, Artemisia argyi), either directly or indirectly atop an insulating medium. Acupoint Catgut Embedding entails the subcutaneous implantation of absorbable sutures at acupoints to provoke a sustained, mild stimulatory effect. Auricular acupressure involves the application of seeds or magnetic beads on specific ear acupoints, which patients are instructed to press periodically for self-stimulation.

However, due to the diversity of acupuncture-related therapies, differing focuses on efficacy, and inconsistent conclusions, previous systematic reviews and meta-analyses (20–23) have only compared single control groups and lack clinical evidence for direct comparisons of efficacy between different acupuncture-related therapies. Therefore, this study employs a network meta-analysis approach to compare the efficacy and safety of various acupuncture-related therapies in the treatment of UC, aiming to provide evidence-based guidance for the use of Traditional Chinese Medicine in treating UC.

## 2 Methods

The protocol for this systematic review and network meta-analysis has been registered in PROSPERO (CRD420251082924). This study followed the PRISMA 2020 (Preferred Reporting Items for Systematic Reviews and Meta-Analyses) guidelines and the PRISMA extension statement for network meta-analyses (PRISMA-NMA) (24, 25), as detailed in Supplementary Material 1.

### 2.1 Search strategy

We searched PubMed, Embase, the Cochrane Library, Web of Science, CBM, CNKI, Wanfang, and VIP databases from inception to June 15, 2025. The search was conducted using a combination of subject headings and free words, and the language was limited to Chinese and English. Search keywords include terms such as “acupuncture, electroacupuncture, warm needling, moxibustion, ulcerative colitis, randomized controlled trials.” Additionally, relevant conference proceedings and clinical trial registries were searched for gray literature. A detailed search strategy is provided in Supplementary Material 2.

### 2.2 Inclusion and exclusion criteria

#### 2.2.1 Inclusion criteria

(1)    S (Study design): Published literature of RCTs, including English and Chinese publications;
(2)    P (Participants): Patients with a clear diagnosis of UC, regardless of gender, age, and course of the disease. The clinical diagnostic criteria of UC are mainly combined with clinical manifestations, endoscopy, histopathology, and laboratory tests;
(3)    I (Interventions): The intervention group was composed of various acupuncture-related therapies, including acupuncture (ACU), warm acupuncture (WA), electroacupuncture (EA), moxibustion (MOX), acupoint catgut embedding (ACE), and auricular acupressure (AA). These therapies can be used alone, in combination, or in conjunction with the control group. Combination therapy (CT) included auricular acupressure combined with Chinese herbal medicine (AA + CHM); acupuncture combination therapy (ACU-CT); moxibustion combination therapy (MOX-CT); warm acupuncture combination therapy (WA-CT); acupoint catgut embedding combination therapy (ACE-CT); electroacupuncture combination therapy (EA-CT);
(4)    C (Comparators): The interventions in the control group included Western medicine (WM), retention enema (RE), traditional Chinese medicine (CHM) or Western medicine combined with retention enema (WM + RE);
(5)    O (Outcomes): Primary outcomes included total effective rate, Mayo score and Baron endoscopic score. Secondary outcomes included IL-6 levels, TNF-α levels, and recurrence rates.

#### 2.2.2 Excluded criteria

(1)    Non-randomized controlled trials, such as case reports, observational studies, reviews, or animal experiments;
(2)    Studies in which patients received multiple concurrent interventions at the same time, such as psychotherapy, cupping, or scraping, or in which the control group also received acupuncture, resulting in the inability to assess the effects of acupuncture independently;
(3)    Studies that failed to report original outcome data or had unclear efficacy criteria and could not be contacted for further information;
(4)    Studies with small sample sizes or treatment durations of < 3 weeks.

### 2.3 Study selection and data extraction

Two researchers independently managed the literature using EndNote 20. The retrieved literatures were first deduplicated, and then titles, abstracts, and full texts were reviewed to select studies that met the inclusion criteria. Disagreements were resolved through consultation or with the assistance of a third researcher. Finally, two researchers cross-checked the literatures. After identifying the eligible studies, data extraction was performed using a pre-designed Excel spreadsheet. The extracted information included: title, first author, year of publication, sample size, age, disease duration, intervention, intervention details, duration of treatment, outcomes, and adverse events.

### 2.4 Quality assessment

Two researchers independently evaluated the risk of bias using the Cochrane risk of bias tool (RoB 2.0) (26) across five domains: randomization process, deviations from intended interventions, missing outcome data, measurement of the outcome, and selection of the reported results. The results of the assessment were categorized as “low risk,” “some concerns,” or “high risk.” Disagreements were resolved through discussion or by consultation with a third researcher.

### 2.5 Certainty of evidence

Two researchers assessed the certainty of evidence for the outcomes using the GRADE approach with GRADE pro GDT software (Grading of Recommendations, Assessment, Development and Evaluation) (27). The GRADE system evaluates the quality of evidence based on six domains: Study design, risk of bias, inconsistency, indirectness, imprecision, and publication bias. The overall quality of evidence is classified into four levels: High, moderate, low, and very low.

### 2.6 Statistical analysis

Network meta-analysis was performed using StataMP 18.0. For dichotomous variables, total effective rate was expressed as the risk ratio (RR), and recurrence rate was represented by the odds ratio (OR). For continuous variables, such as Mayo score, Baron endoscopic score, IL-6, and TNF-α levels, the standardized mean difference (SMD) was used as the Effect size measure, with both expressed with 95% confidence intervals (CIs). A statistically significant difference between groups was considered when the 95% CI for dichotomous variables did not include 1 or for continuous variables did not include 0. Heterogeneity was quantified using τ^2^, categorized as low (< 0.04), low to moderate (0.04–0.16), moderate to high (0.16–0.36), or high (> 0.36) (28–30). Consistency between direct and indirect comparisons was evaluated using the node splitting method (31). The surface under the cumulative ranking curve (SUCRA) was used to compare the efficacy of different interventions, with a larger area indicating better efficacy of the intervention (32). Funnel plots were used to assess publication bias in the included literatures (33).

## 3 Results

### 3.1 Literature search results

**FIGURE 1:**
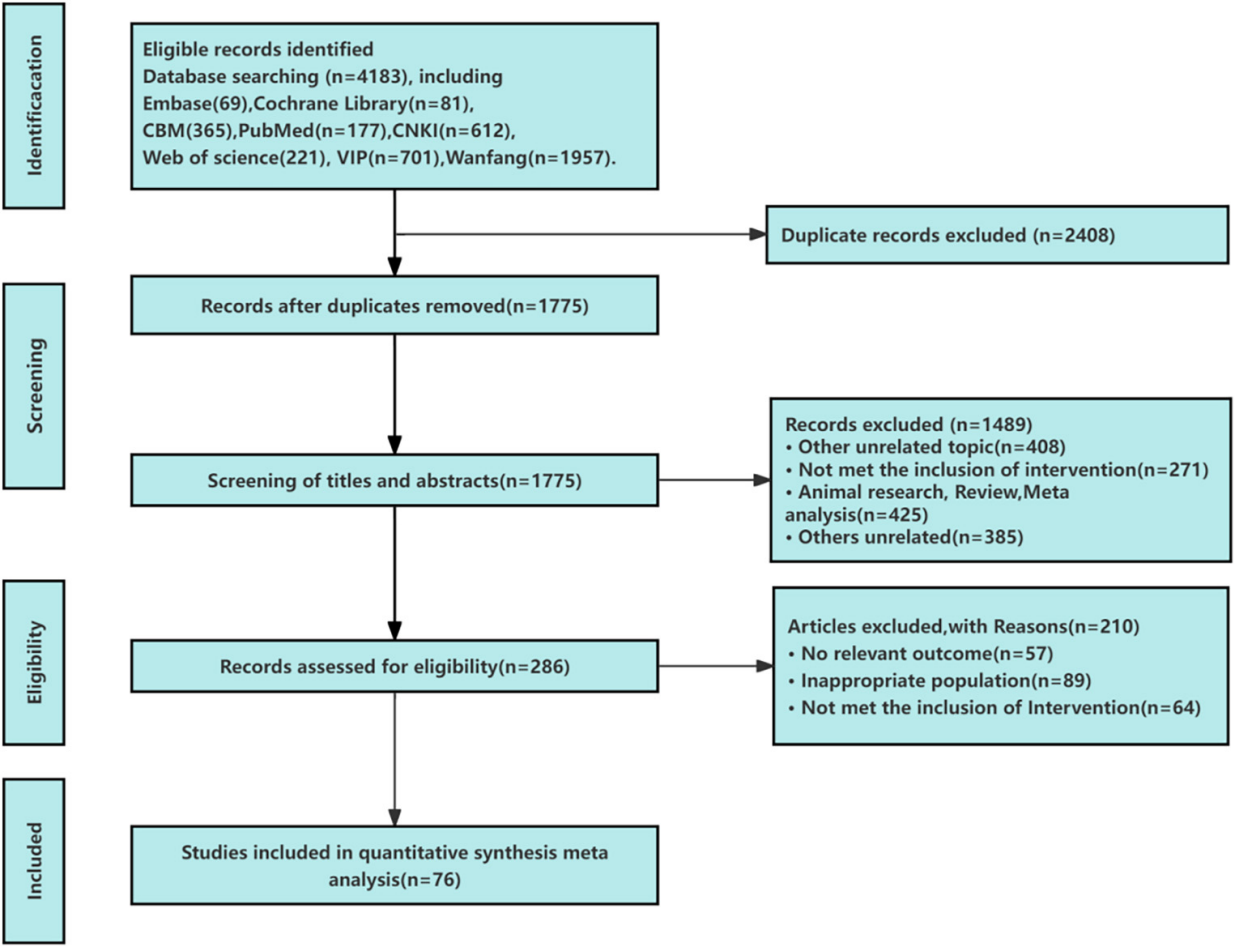
Flow chart of the search for eligible RCTs.

A total of 4183 relevant articles were retrieved through database search, 2408 duplicate articles were eliminated by Endnote 20 software, and 286 articles were obtained by reading the titles and abstracts according to the inclusion and exclusion criteria. After reading the full text, 210 articles were excluded. A total of 76 articles were finally determined to meet the inclusion criteria. The literature screening process is shown in Figure 1.

### 3.2 Included study characteristics

A total of 76 randomized controlled trials were included in this study (13–15, 34–106), with 3,847 cases in the experimental group and 3,637 cases in the control group. The maximum sample size was 220 and the minimum was 60. The age ranged from 30 to 50 years old. The duration of treatment varied from 3 to 6 weeks. The acupuncture point selection schemes differed among the studies, with high-frequency acupoints including Tianshu (ST25), Zusanli (ST36), Shangjuxu (ST37), Zhongwan (CV12), Guanyuan (CV4), and Dachangshu (BL25). Study characteristics are summarized in Tables 1, 2. Both the acupoint frequency statistics and the standardized acupoint definitions are available in Supplementary Material 3.

**TABLE 1:** Baseline of characteristics of included studies.

Study ID	Sample size (T/C)	Age (Year, T/C)	Disease duration (Year, T/C)	Treatment group	Control group	Duration of treatment	Outcomes
Zhao 2012 (34)	33/32	37.48 ± 9.34 39.52 ± 8.67	2.33 ± 1.65 2.72 ± 1.71	ACE + RE	RE	4 weeks	➀
Zhang 2003 (35)	43/35	39.5 ± 9.25 39 ± 19	8.25 ± 3.88 8.25 ± 3.88	ACU	WM + RE	4 weeks	➀➅
Wang 2022a (36)	67/67	44.27 ± 3.65 44.15 ± 3.57	2.61 ± 0.35 2.57 ± 0.43	ACU + CHM + WM	WM	4 weeks	➀➁
Cheng 2009 (37)	40/40	53 ± 8.5 53 ± 8.5	5.84 ± 2.58 5.84 ± 2.58	MOX + CHM + WM	WM	4 weeks	➀
Fan 2021 (38)	45/45	41.55 ± 5.56 42.38 ± 4.15	4.32 ± 1.03 4.67 ± 1.15	MOX + CHM	WM	4 weeks	➀➂➃➄
Zhou 2008 (39)	110/110	48.60 ± 7.48 50.24 ± 6.95	4.75 ± 1.69 4.89 ± 1.76	EA + MOX + WM	WM	6 weeks	➀➅
Xu 2022 (40)	36/37	42.05 ± 8.56 40.89 ± 9.53	1.12 ± 0.56 1.60 ± 0.77	MOX + CHM	WM	4 weeks	➀➂➄
Zhao 2019 (41)	35/35	47.2 ± 6.8 42.7 ± 8.9	1.44 ± 0.56 1.25 ± 0.11	MOX	WM	12 weeks	➀
Yang 2017 (42)	30/30	41.17 ± 7.43 42.25 ± 6.81	4.13 ± 1.26 3.84 ± 1.47	MOX	WM	8 weeks	➀➁➂
He 2021 (43)	48/48	32.80 ± 4.80 33.70 ± 5.10	0.02 ± 0.01 0.02 ± 0.01	MOX + RE	WM	4 weeks	➁➂➃
Zhao 2018 (44)	55/55	46.25 ± 10.12 45.37 ± 12.46	2.45 ± 1.31 2.83 ± 1.72	ACU + CHM	WM	4 weeks	➀➄
Zhu 2003 (45)	50/20	42.5 ± 11.25 43 ± 11	10.5 ± 4.75 9.92 ± 4.54	ACE	WM	4 weeks	➀
Sun 2017 (46)	36/36	43.53 ± 9.24 44.14 ± 9.47	6.44 ± 2.55 6.47 ± 2.51	MOX	WM	12 weeks	➀
Ma 1997 (47)	60/30	45.5 ± 11.25 46.5 ± 23.25	6.75 ± 3.13 5.25 ± 2.38	ACU	WM	4 weeks	➀
Wang 2006 (48)	30/30	40 ± 6.5/ 41 ± 6.5	0.5∼12 0.6∼13	MOX	WM	4 weeks	➀
Li 2006 (49)	56/60	37.1 37.3	0.25∼2.33 0.25∼2	ACE	WM	4 weeks	➀
Kang 2022 (50)	50/50	40.02 ± 9.11 39.27 ± 9.80	3.77 ± 1.84 3.65 ± 2.41	ACU + CHM	WM	12 weeks	➀➁➃➄
Wu 2015 (51)	52/50	49.5 ± 7.6 48.5 ± 7.3	NR NR	ACU + MOX + CHM	CHM	8 weeks	➀➅
Zhang 2021a (52)	39/38	39.4 ± 11.1 38.9 ± 10.2	4.23 ± 3.15 3.96 ± 2.89	ACU	WM	8 weeks	➀➂
Gong 2020 (53)	50/50	36.2 ± 9.0 39.5 ± 8.5	2.3 ± 1.6 2.6 ± 1.4	ACE	WM	6 weeks	➀➃➄
Feng 2020 (54)	36/36	31.54 ± 3.48 32.22 ± 3.97	5.36 ± 1.83 5.21 ± 2.13	ACU	WM	4 weeks	➀
Zu 2017 (55)	45/45	39.56 ± 10.69 40.76 ± 11.09	3.11 ± 1.30 3.42 ± 1.41	MOX + RE + WM	WM + RE	4 weeks	➀➃➄
Lv 2017a (56)	50/50	40.86 ± 10.83 41.09 ± 10.68	3.19 ± 1.2 3.27 ± 1.28	MOX + CHM + WM	WM	4 weeks	➀➃➄
Guo 2016 (57)	35/35	46.25 ± 15.11 50.2 ± 14.19	5.08 ± 2.1 4.83 ± 1.74	ACU + MOX + WM	WM	6 weeks	➀
Chen 2004 (58)	100/30	44.5 ± 13.25 42 ± 11.5	11.13 ± 5.44 10.13 ± 4.84	ACE	WM	24 weeks	➀
Chang 2017 (59)	45/45	38.24 ± 9.79 40.01 ± 11.23	3.05 ± 1.20 3.16 ± 1.41	MOX + RE + WM	WM + RE	6 weeks	➀➃➄
Wang 2020a (60)	32/32	27.24 ± 2.23 26.12 ± 5.09	5.77 ± 1.99 5.34 ± 2.12	EA + CHM + WM	WM	8 weeks	➀
Xu 2009 (61)	40/40	47.37 ± 5.17 49.13 ± 4.92	2.79 ± 3.91 3.03 ± 4.18	ACU + CHM	WM	6 weeks	➀
Gu 2016 (62)	38/37	40.5 ± 2.8 42.5 ± 3.2	4.9 ± 1.3 5.2 ± 2.12	MOX + CHM	WM	8 weeks	➀➅
Zhang 2022 (13)	47/47	44 ± 9 46 ± 8	3.41 ± 0.73 3.59 ± 0.80	EA + WM	WM	8 weeks	➀➃
Xie 2019 (63)	65/65	36 ± 8 35 ± 8	3.40 ± 1.03 3.34 ± 1.01	ACU + CHM	CHM	4 weeks	➀➄
Shen 2019 (64)	50/50	37 ± 9 38 ± 10	0.9 ± 0.1 0.8 ± 0.1	MOX + WM	WM	12 weeks	➀➅
Shen 2012 (65)	33/30	46 ± 7 41 ± 7	3.5 ± 1.3 3.8 ± 1.6	ACU	WM	12 weeks	➀
Zong 2015 (66)	34/32	43 ± 12 42.5 ± 11.25	1.05 ± 0.275 3.55 ± 1.225	ACE	WM	6 weeks	➀➅
Du 2008 (67)	42/40	46 ± 9.5 43.5 ± 9.25	14.7 ± 6.65 15.75 ± 7.125	ACU + RE + CHM	WM + RE	4 weeks	➀
Hou 2018 (68)	48/48	35.76 ± 4.53 36.27 ± 4.36	4.69 ± 1.21 4.73 ± 1.17	MOX + WM	WM	4 weeks	➀
Ye 2024 (69)	60/60	39.49 ± 4.28 39.44 ± 4.38	3.19 ± 0.42 3.23 ± 0.39	ACU + CHM	WM	4 weeks	➀
Wang 2022b (70)	30/30	47.22 ± 4.02 47.42 ± 4.03	4.55 ± 1.29 4.49 ± 1.37	ACU	WM	4 weeks	➀
Du 2007 (71)	89/80	43.6 41.7	4.8 ± 1.4 4.7 ± 2.1	AA + CHM	WM	4 weeks	➀
Cong 2018 (72)	75/72	38.61 ± 11.74 37.82 ± 12.67	3.75 ± 2.81 3.61 ± 2.47	ACU + CHM	WM	6 weeks	➀➁➃➄
Zhou 2003 (73)	34/32	38.5 ± 9.75 38.5 ± 9.75	5 ± 1.5 5 ± 1.5	MOX	WM	4 weeks	➀
Ge 2014 (74)	31/31	35.6 ± 7.5 38.4 ± 7.8	3.7 ± 2.8 4.0 ± 2.5	EA + WM	WM	8 weeks	➀
Zhang 2012 (75)	45/45	37.63 ± 8.71 35.27 ± 8.56	6.32 ± 3.11 6.95 ± 3.23	ACU + RE	WM	4 weeks	➀
Chen 2013 (76)	40/40	47.5 ± 12.25 46.5 ± 13.75	8.5 ± 3.25 9.5 ± 4.25	MOX + RE + WM	WM + RE	4 weeks	➀➅
Xu 2023 (77)	50/51	39.54 ± 4.52 39.66 ± 4.49	1.99 ± 0.47 1.96 ± 0.50	ACU + CHM + RE	RE	4 weeks	➀➁➅
Wang 2020b (78)	36/36	35.0 ± 3.2 34.8 ± 2.9	7.0 ± 2.1 6.9 ± 1.8	ACU + CHM + WM	WM	6 weeks	➀
Teng 2014 (79)	40/40	38.5 ± 8.75 39.5 ± 8.75	5.2 ± 2.4 5.75 ± 2.625	EA + RE + WM	WM	8 weeks	➀
Zhang 2009 (80)	50/40	40.73 ± 8.1 38.73 ± 7.5	4.2 ± 1.2 4.1 ± 1.7	ACU + CHM	WM	4 weeks	➀➅
Cao 2001 (81)	40/40	50 ± 14 47.5 ± 13.75	4.21 ± 1.90 4.25 ± 1.875	ACU + RE	WM + RE	6 weeks	➀
Bao 2014 (82)	50/50	41. .2 ± 6. 7 42. .40. 3 ± 5. 7	4. 3 ± 1. 2 4. 5 ± 1. 6	ACU + RE	WM	4 weeks	➀
He 2015 (83)	30/30	43.5 ± 9.8 44.2 ± 10.2	4.1 ± 2.3 4.2 ± 1.9	ACU + WM	WM	4 weeks	➀➄
Hui 2012 (84)	60/60	49.63 ± 6.12 48.65 ± 5.11	3.54 ± 3.96 3.34 ± 4.21	MOX + RE	WM	8 weeks	➀➅
Lu 2021 (85)	40/40	38.12 ± 8.28 37.57 ± 7.65	5.25 ± 3.50 4.77 ± 3.26	MOX + CHM	WM	4 weeks	➀➁➂➄
Wang 2010 (86)	45/45	46.5 ± 11.75 46.5 ± 11.75	6.25 ± 2.88 6.25 ± 2.88	ACU + RE	WM	3 weeks	➀
Li 2016 (87)	30/30	43.18 ± 11.63 44.82 ± 2.52	NR 4.36 ± 2.52	WA	WM	8 weeks	➀
Zhang 2020 (88)	34/34	39.3 ± 10.1 38.5 ± 9.9	4.21 ± 3.03 3.96 ± 2.61	WA	WM	8 weeks	➀➁➂➃
Wang 2020c (89)	40/40	38.3 ± 4.3 38.2 ± 4.1	2.4 ± 0.6 2.3 ± 0.3	ACU + CHM + WM	WM	4 weeks	➀➁
Wang 2008 (90)	80/78	44.5 ± 13.25 44.5 ± 12.25	19 ± 8 17.75 ± 7.625	MOX + CHM	WM + RE	6 weeks	➀
Lv 2017b (91)	46/46	39.78 ± 10.58 40.47 ± 11.36	3.12 ± 1.31 3.26 ± 1.39	MOX + RE + WM	WM	6 weeks	➀➃➄
Jia 2015 (92)	32/32	44 ± 8 40.5 ± 8.25	4.5 ± 1.75 4 ± 1.5	MOX + WM	WM	8 weeks	➀
Zhang 2021b (14)	52/50	32.05 ± 5.08 32.12 ± 5.10	3.52 ± 1.23 3.28 ± 1.01	WA + CHM + WM	WM	8 weeks	➀➃➄
Zheng 2020 (93)	40/40	42.63 ± 10.45 46.70 ± 10.70	11.46 ± 3.52 10.63 ± 2.95	MOX + RE	WM	4 weeks	➀➁➄
Li 2024 (94)	30/30	34.27 ± 8.33 32.07 ± 8.92	2.42 ± 1.19 3.01 ± 1.13	ACU + WM	WM	8 weeks	➀➁➅
Xiu 2020 (95)	36/36	36.10 ± 3.11 35.92 ± 3.13	2.29 ± 0.79 2.30 ± 0.75	MOX + CHM + WM	WM	8 weeks	➀➂➄
Zhou 2021 (15)	47/46	36.52 ± 6.24 35.24 ± 5.45	8.32 ± 1.16 8.05 ± 1.12	WA + CHM + WM	WM	4 weeks	➀➃➄
Hua 2022 (96)	59/59	47.06 ± 4.10 47.11 ± 3.25	2.68 ± 0.29 2.74 ± 0.35	ACU + CHM + WM	WM	12 weeks	➀➁➂➄
Wang 2013 (97)	45/45	43.65 ± 13.58 44.56 ± 15.01	NR NR	ACE	WM	4 weeks	➀
Han 2009 (98)	50/40	40.73 ± 8.1 38.73 ± 7.5	4.2 ± 1.2 4.1 ± 1.7	ACU	WM	4 weeks	➀➅
Du 2008a (99)	89/80	43.61 ± 10.16 41.76 ± 1.3	4.5 ± 1.4 4.1 ± 1.2	AA + CHM	WM	4 weeks	➀➅
Zhang 2010 (100)	40/40	42.64 ± 6.91 38.73 ± 7.52	3.91 ± 2.3 4.12 ± 1.73	ACU + WM	WM	6 weeks	➀➅
Du 2008b (101)	89/80	44.5 ± 11.75 41.5 ± 11.75	4.3 ± 1.4 4.7 ± 1.4	AA + CHM	WM	4 weeks	➀
Lin 2023 (102)	30/33	48 ± 11 47.5 ± 11.25	17.5 ± 8.25 15.15 ± 7.43	MOX	WM	12 weeks	➀
Wang 2012 (103)	40/30	44 ± 3.27 42 ± 3.31	7.75 ± 3.63 7.71 ± 3.65	MOX + RE	RE	7 weeks	➀
Duan 2012 (104)	320/320	45.5 ± 14.75 46 ± 14.5	9.63 ± 4.69 9.63 ± 4.69	ACE + RE	RE	8 weeks	➀
He 2001 (105)	36/35	41.5 ± 13.25 41.5 ± 13.25	10.09 ± 4.96 10.09 ± 4.96	ACU + RE	RE	4 weeks	➀
Li 2008 (106)	32/31	34.6 36.5	6.13 ± 2.94 5.75 ± 2.63	ACU + MOX + RE	RE	4 weeks	➀➅

ACU, acupuncture; MOX, moxibustion; WA, warm acupuncture; ACE, acupoint catgut embedding; EA, electroacupuncture; AA, auricular acupressure; CHM, Chinese Herbal Medicine; RE, retention enema; WM, western medicine; CT, combination therapy; T, treatment group; C, control group.

**TABLE 2:** Descriptions of the included acupuncture and related therapies.

Study ID	Acupuncture-related therapy	Acupoints	Treatment schedule
Zhao 2012 (34)	ACE + RE	Tianshu(ST25) Xiawan(CV10) Shangjuxu(ST37) Guanyuan(CV4) Quchi(LI11) Neiting(ST44)	Once per week
Zhang 2003 (35)	ACU	Sanjiaoshu(BL22) Dachangshu(BL25)	30 min per session, once daily; 10 days per treatment course
Wang 2022a (36)	ACU + CHM + WM	Shangjuxu(ST37) Yinlingquan(SP9) Tianshu(ST25) Zhigou(TE6) Neiting(ST44) Dachangshu(BL25) Shuidao(ST28) Wailing(ST26)	30 min per session, once daily; 5 days per course with 2-day interval before the next
Cheng 2009 (37)	MOX + CHM + WM	Zusanli(ST36) Shangjuxu(ST37) Xiajuxu(ST39) Fenglong(ST40) Shousanli(LI10) Hegu(LI4)	10 s per session
Fan 2021 (38)	MOX + CHM	Shenque(CV8)	40–45 min per session, once daily; 7 days per treatment course
Zhou 2008 (39)	EA + MOX + WM	Tianshu(ST25) Zhongwan(CV12) Qihai(CV6) Zusanli(ST36) Shangjuxu(ST37) Yinlingquan(SP9)	30 min per session, once daily; 10 days per course with 2-day interval
Xu 2022 (40)	MOX + CHM	Tianshu(ST25) Zhongwan(CV12) Pishu(BL20) Huiyang(BL35)	30 min per session, once per week
Zhao 2019 (41)	MOX	Shenque(CV8)	1.5 h per session, twice weekly
Yang 2017 (42)	MOX	Pishu(BL20) Zhongwan(CV12) Zusanli(ST36) Dachangshu(BL25) Tianshu(ST25) Shangjuxu(ST37)	15 min per session, once daily
He 2021 (43)	MOX + RE	Dachangshu(BL25) Tianshu(ST25) Shangjuxu(ST37)	Once daily; 10 days per course with 2-day interval before the next
Zhao 2018 (44)	ACU + CHM	Tianshu(ST25) Shangjuxu(ST37)	30 min per session, once daily; 6 days treatment, 1 day rest
Zhu 2003 (45)	ACE	Zhongwan(CV12) Zusanli(ST36) Tianshu(ST25)	Once every 2 weeks
Sun 2017 (46)	MOX	Shenque(CV8)	2 h per session
Ma 1997 (47)	ACU	Tianshu(ST25) Zhongwan(CV12) Guanyuan(CV4) Zusanli(ST36) Taichong(LR3) Dachangshu(BL25) Shenshu(BL23) Shangliao(BL31) Ciliao(BL32)	30 min per session, once daily
Wang 2006 (48)	MOX	Shenque(CV8)	Once daily; 10-day course with 3-day interval before the next
Li 2006 (49)	ACE	Dachangshu(BL25) Zusanli(ST36) Shangjuxu(ST37)	Not reported
Kang 2022 (50)	ACU + CHM	Zhongwan(CV12) Xiawan(CV10) Qihai(CV6) Guanyuan(CV4) Daheng(SP15) Wailing(ST26) Huaroumen(ST24)	30 min per session, three times per week
Wu 2015 (51)	ACU + MOX + CHM	Zhongwan(CV12) Xiawan(CV10) Shenque(CV8) Tianshu(ST25) Guilai(ST27) Qihai(CV6) Guanyuan(CV4) Zusanli(ST36) Yinlingquan(SP9)	ACU: 30 min/session, once daily, 10-day course; MOX: every other day
Zhang 2021a (52)	WA + WM	Pishu(BL20) Zhongwan(CV12) Zusanli(ST36) Tianshu(ST25) Taibai(SP3) Shangjuxu(ST37) Sanyinjiao(SP6) Guanyuan(CV4)	20 min per session, five times per week; 10 days per course
Gong 2020 (53)	ACE	Zusanli(ST36) Guanyuan(CV4) Pishu(BL20)	Not reported
Feng 2020 (54)	ACU	Guanyuan(CV4) Qihai(CV6) Changqiang(GV1) Dachangshu(BL25) Tianshu(ST25)	20 min per session, once daily
Zu 2017 (55)	MOX + RE + WM	Dachangshu(BL25) Pishu(BL20) Shangjuxu(ST37) Xiajuxu(ST39) Tianshu(ST25)	Saturating desensitization moxibustion dose, once daily; 14-day course, 2–3 day interval
Lv 2017a (56)	MOX + CHM + WM	Dachangshu(BL25) Pishu(BL20) Shangjuxu(ST37) Xiajuxu(ST39) Tianshu(ST25)	Saturating moxibustion dose, once daily; 14-day course
Guo 2016 (57)	ACU + MOX + WM	Shenshu(BL23) Pishu(BL20) Dachangshu(BL25) Weishu(BL21) Gongsun(SP4) Tianshu(ST25) Zusanli(ST36) Taixi(KI3) Zhongwan(CV12) Guanyuan(CV4)	ACU: 30 min/session, once daily; MOX: 5 moxa cones per acupoint, once daily; 10-day course with 2-day interval
Chen 2004 (58)	ACE	Dachangshu(BL25) Tianshu(ST25) Zusanli(ST36)	Once per month
Chang 2017 (59)	MOX + RE + WM	Shenque(CV8)	40 min per session, once daily
Wang 2020a (60)	EA + CHM + WM	Zhongwan(CV12) Tianshu(ST25) Zusanli(ST36) Yinlingquan(SP9) Pishu(BL20) Taichong(LR3)	30 min per session, once daily; 4-week treatment course
Xu 2009 (61)	ACU + CHM	Shangjuxu(ST37) Xiajuxu(ST39) Yinlingquan(SP9) Gongsun(SP4) Tianshu(ST25) Hegu(LI4) Quchi(LI11)	30–40 min per session, once daily
Gu 2016 (62)	MOX + CHM	Shenque(CV8) Mingmen(GV4) Guanyuan(CV4) Zhongwan(CV12) Zusanli(ST36) Tianshu(ST25) Dachangshu(BL25)	Eight moxa cones per point, twice daily
Zhang 2022 (13)	EA + WM	Pishu(BL20) Zhongwan(CV12) Zusanli(ST36) Tianshu(ST25) Taibai(SP3) Shangjuxu(ST37) Sanyinjiao(SP6) Guanyuan(CV4)	20 min per session, once daily; 5-day course with 2-day interval
Xie 2019 (63)	ACU + CHM	Pishu(BL20) Guanyuan(CV4) Weishu(BL21) Tianshu(ST25) Zhongwan(CV12) Zusanli(ST36) Qihai(CV6) Dachangshu(BL25) Gongsun(SP4)	20–30 min per session, once daily; 1-week treatment course
Shen 2019 (64)	MOX + WM	Zhongwan(CV12) Tianshu(ST25) Guanyuan(CV4) Shangjuxu(ST37)	Two moxa cones per point, once daily; 12-day course with 3-day interval
Shen 2012 (65)	ACU	Zhongwan(CV12) Tianshu(ST25) Guanyuan(CV4) Zusanli(ST36) Shenque(CV8)	30 min per session, once daily; 10-day course with 7-day interval
Zong 2015 (66)	ACE	Zhongwan(CV12) Zusanli(ST36) Tianshu(ST25)	Once every 2 weeks
Du 2008 (67)	ACU + RE + CHM	Zusanli(ST36) Shangjuxu(ST37) Xiajuxu(ST39)	30 min per session, once daily
Hou 2018 (68)	MOX + WM	Qihai(CV6) Tianshu(ST25) Zhongwan(CV12) Yinlingquan(SP9) Shangjuxu(ST37)	Burn moxa fully per session, once daily; 15-day course with 3-day interval
Ye 2024 (69)	ACU + CHM	Tianshu(ST25) Quchi(LI11) Shangjuxu(ST37)	Two to three times per week
Wang 2022b (70)	ACU	Dachangshu(BL25) Tianshu(ST25)	30 min per session, once daily
Du 2007 (71)	AA + CHM	Spleen(MA-IC 3) Large Intestine(MA-IC 5) Endocrine(MA-AT 1) Sympathetic(MA-AH 7) Subcortex(MA-AT 4)	Press 3–5 times daily; 10–20 presses per acupoint
Cong 2018 (72)	ACU + CHM	Dachangshu(BL25) Shenque(CV8) Tianshu(ST25) Shangjuxu(ST37) Sanyinjiao(SP6) Pishu(BL20) Hegu(LI4) Xiajuxu(ST39) Zusanli(ST36) Guanyuan(CV4) Quchi(LI11) Neiting(ST44)	15 min per session, once daily; 5-day course with 2-day interval
Zhou 2003 (73)	MOX	Zhongwan(CV12) Tianshu(ST25) Zusanli(ST36)	5–7 moxa cones until skin reddens, once daily; 10-day course
Ge 2014 (74)	EA + WM	Ganshu(BL18) Pishu(BL20) Dachangshu(BL25) Shenshu(BL23) Ciliao(BL32) Tianshu(ST25) Qihai(CV6) Guanyuan(CV4) Shangjuxu(ST37) Sanyinjiao(SP6) Taichong(LR3)	30 min per session, once daily; 5-day course with 2–3 day interval
Zhang 2012 (75)	ACU + RE	Pishu(BL20) Zhongwan(CV12) Tianshu(ST25) Zusanli(ST36) Shangjuxu(ST37)	30 min per session, once daily; 2-week treatment course
Chen 2013 (76)	MOX + RE + WM	Tianshu(ST25) Shenque(CV8) Guanyuan(CV4) Zhongwan(CV12) Zusanli(ST36) Qihai(CV6) Pishu(BL20) Shangjuxu(ST37) Yinlingquan(SP9)	Not reported
Xu 2023 (77)	ACU + CHM + RE	Yinbai(SP1) Shaoshang(LU11)	20 min per session, every other day
Wang 2020b (78)	ACU + CHM + WM	Dachangshu(BL25) Shangjuxu(ST37) Xiajuxu(ST39) Shenque(CV8) Tianshu(ST25) Zusanli(ST36)	15 min per session, once daily; 5-day course with 2-day interval
Teng 2014 (79)	EA + RE + WM	Zhongwan(CV12) Tianshu(ST25) Guanyuan(CV4) Zusanli(ST36) Sanyinjiao(SP6)	Not reported
Zhang 2009 (80)	ACU + CHM	Scalp Acupuncture Stomach Zone Scalp Acupuncture Intestinal Zone	5–10 min per session, once daily
Cao 2001 (81)	ACU + RE	Pishu(BL20) Zhangmen(LR13) Tianshu(ST25) Zusanli(ST36) Zhongwan(CV12) Guanyuan(CV4) Mingmen(GV4) Gongsun(SP4)	20 min per session, once daily
Bao 2014 (82)	ACU + RE	Guanyuan(CV4) Tianshu(ST25) Zusanli(ST36) Yinlingquan(SP9) Sanyinjiao(SP6)	20 min per session, once daily
He 2015 (83)	ACU + WM	Tianshu(ST25) Qihai(CV6) Guanyuan(CV4) Hegu(LI4) Shangjuxu(ST37) Sanyinjiao(SP6)	Once daily; 5-day course with 2-day interval
Hui 2012 (84)	MOX + RE	Shenque(CV8) Zhongwan(CV12) Zusanli(ST36)	Three moxa cones per acupoint, once daily
Lu 2021 (85)	MOX + CHM	Shenque(CV8)	20 min per session, once every 2 days
Wang 2010 (86)	ACU + RE	Tianshu(ST25) Daheng(SP15) Zusanli(ST36) Shenque(CV8)	30 min per session, once daily; 7-day course with 1–2 day interval
Li 2016 (87)	WA	Tianshu(ST25) Qihai(CV6) Guanyuan(CV4) Changqiang(GV1) Dachangshu(BL25) Zusanli(ST36) Sanyinjiao(SP6)	20 min per session, once every 2 days
Zhang 2020 (88)	WA	Pishu(BL20) Zhongwan(CV12) Zusanli(ST36) Tianshu(ST25) Taibai(SP3) Shangjuxu(ST37) Sanyinjiao(SP6) Guanyuan(CV4)	20 min per session, 5 times per week; 10 sessions per course
Wang 2020c (89)	ACU + CHM + WM	Quchi(LI11) Hegu(LI4) Tianshu(ST25) Neiting(ST44) Shangjuxu(ST37)	15–20 min per session, once daily; 5-day course with 2-day interval
Wang 2008 (90)	MOX + CHM	Shenque(CV8) Zusanli(ST36) Tianshu(ST25) Shangjuxu(ST37)	20 min per session, once daily; 10-day course with 2-day interval
Lv 2017b (91)	MOX + RE + WM	Dachangshu(BL25) Pishu(BL20) Shangjuxu(ST37) Xiajuxu(ST39) Tianshu(ST25)	15 min per session, once daily
Jia 2015 (92)	MOX + WM	Zhongji(CV3) Guanyuan(CV4) Qihai(CV6) Tianshu(ST25) Daheng(SP15) Dachangshu(BL25) Zusanli(ST36) Shangjuxu(ST37) Sanyinjiao(SP6) Taichong(LR3)	Once daily
Zhang 2021b (14)	WA + CHM + WM	Zhongwan(CV12) Guanyuan(CV4) Qihai(CV6) Zusanli(ST36) Tianshu(ST25) Yinlingquan(SP9)	Once daily; 6-day course with 1-day interval
Zheng 2020 (93)	MOX + RE	Shenque(CV8)	30 min per session, once daily; 7-day course
Li 2024 (94)	ACU + WM	Tianshu(ST25)	20 min per session, once daily; 5-day course with 2-day interval
Xiu 2020 (95)	MOX + CHM + WM	Zhongwan(CV12) Guanyuan(CV4) Qihai(CV6) Tianshu(ST25)	30–40 min per session, once daily
Zhou 2021 (15)	WA + CHM + WM	Guanyuan(CV4) Zhongwan(CV12) Tianshu(ST25) Zusanli(ST36) Shangjuxu(ST37) Pishu(BL20) Shenshu(BL23) Dachangshu(BL25)	30 min per session, once daily; 5 days per course with 2-day interval before the next
Hua 2022 (96)	ACU + CHM + WM	Quchi(LI11) Tianshu(ST25) Guanyuan(CV4) Shangjuxu(ST37) Zusanli(ST36)	30 min per session, once daily; 5 days per course with 2-day interval before the next
Wang 2013 (97)	ACE	Pishu(BL20) Weishu(BL21) Dachangshu(BL25)	Once every 15 days
Han 2009 (98)	ACU	Scalp Acupuncture Stomach Zone Scalp Acupuncture Intestinal Zone	5–10 min per session, once daily
Du 2008a (99)	AA + CHM	Spleen(MA-IC 3) Large Intestine(MA-IC 5) Endocrine(MA-AT 1) Sympathetic(MA-AH 7) Subcortex(MA-AT 4)	Press 3–5 times daily; 10–20 presses per acupoint
Zhang 2010 (100)	ACU + WM	Scalp Acupuncture Stomach Zone Scalp Acupuncture Intestinal Zone	5–10 min per session, once daily; 20-day course with 2-day interval
Du 2008b (101)	AA + CHM	Spleen(MA-IC 3) Large Intestine(MA-IC 5) Endocrine(MA-AT 1) Sympathetic(MA-AH 7) Subcortex(MA-AT 4)	Press 3–5 times daily; 10–20 presses per acupoint
Lin 2023 (102)	MOX	Qihai(CV6) Tianshu(ST25) Shangjuxu(ST37) Xiajuxu(ST39)	Three times per week
Wang 2012 (103)	MOX + RE	Shenque(CV8)	6–8 h
Duan 2012 (104)	ACE + RE	Tianshu(ST25) Dachangshu(BL25) Guanyuan(CV4) Zhongwan(CV12) Zusanli(ST36) Yinlingquan(SP9)	Once every 2 weeks; 14-day course with 7-day interval
He 2001 (105)	ACU + RE	Zusanli(ST36) Shangjuxu(ST37) Xiajuxu(ST39)	30 min per session, once daily; 14-day course with 3-day interval
Li 2008 (106)	ACU + MOX + RE	Zhongwan(CV12) Qihai(CV6) Zusanli(ST36) Sanyinjiao(SP6) Hegu(LI4) Dachangshu(BL25) Tianshu(ST25) Shangjuxu(ST37)	30 min per session

ACU, acupuncture; MOX, moxibustion; WA, warm acupuncture; ACE, acupoint catgut embedding; EA, electroacupuncture; AA, auricular acupressure; CHM, Chinese Herbal Medicine; RE, retention enema; WM, western medicine.

### 3.3 Risk of bias, certainty of evidence, and consistency

**FIGURE 2:**
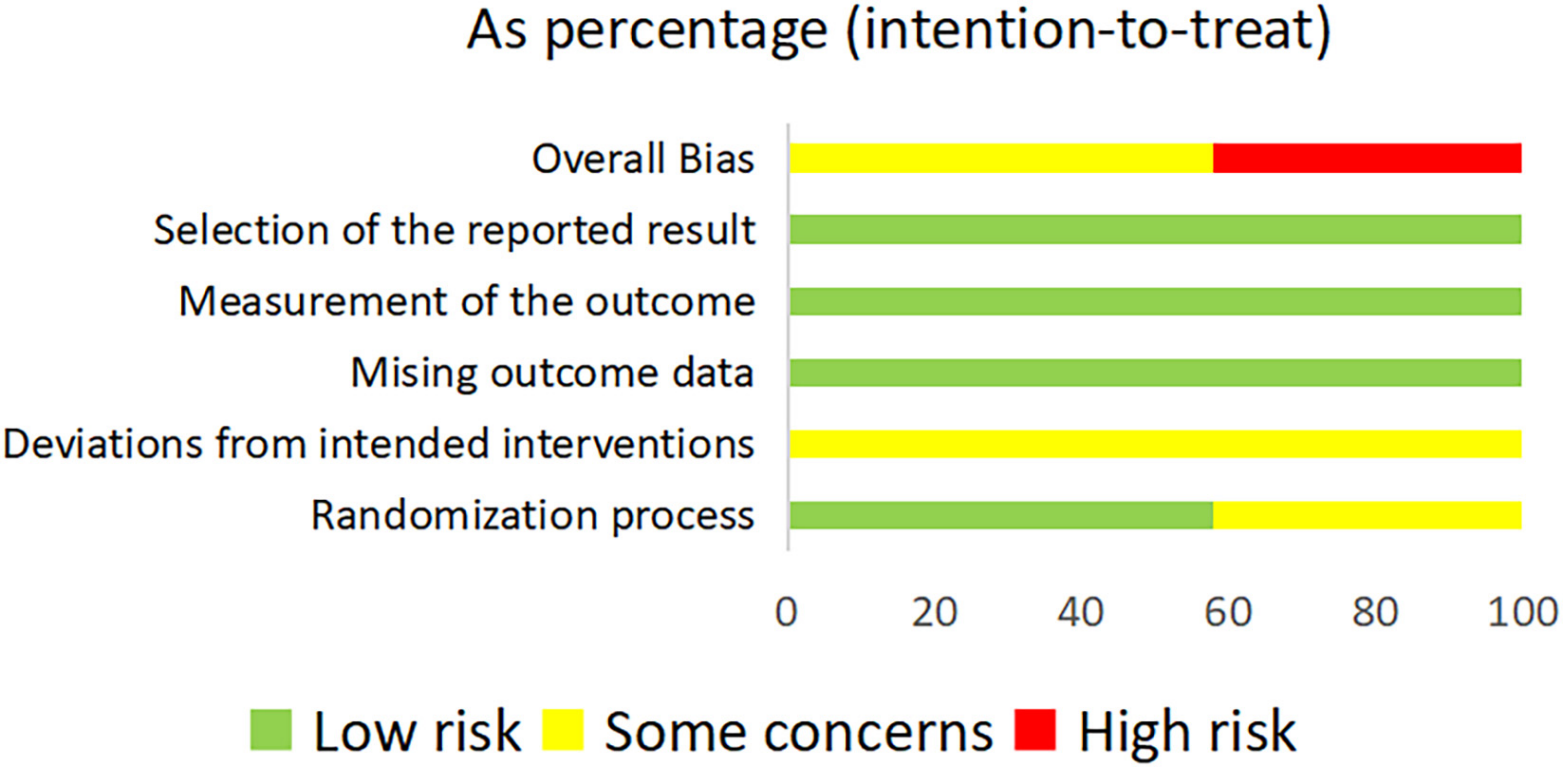
Assessment of risk of bias.

A total of 76 RCTs were included in this study. 32 studies (42.1%) were rated as high risk of bias due to unclear randomization. 76 studies (100%) did not describe any blinding. Considering the characteristics of the intervention, the risk of bias was judged to be some concerns. 76 studies (100%) had a low risk of missing outcome data and outcome measurement bias. There was no evidence of selective reporting or other potential sources of bias. Overall, 41 studies (57.9%) were rated as some concerns, while 35 studies (42.1%) were rated as high risk. A summary of the risk assessment is provided in Supplementary Material 4 and illustrated in Figure 2. As no closed loops were formed between the included interventions, consistency testing was deemed unnecessary. The consistency model demonstrated good agreement across all comparisons, with no significant inconsistency observed between direct and indirect evidence. Heterogeneity was assessed using τ^2^, and the results showed low heterogeneity in dichotomous variables, indicating high consistency between studies. For continuous data, there was moderate to high heterogeneity (Supplementary Material 5).

### 3.4 Network meta-analysis results

#### 3.4.1 Network plots

**FIGURE 3:**
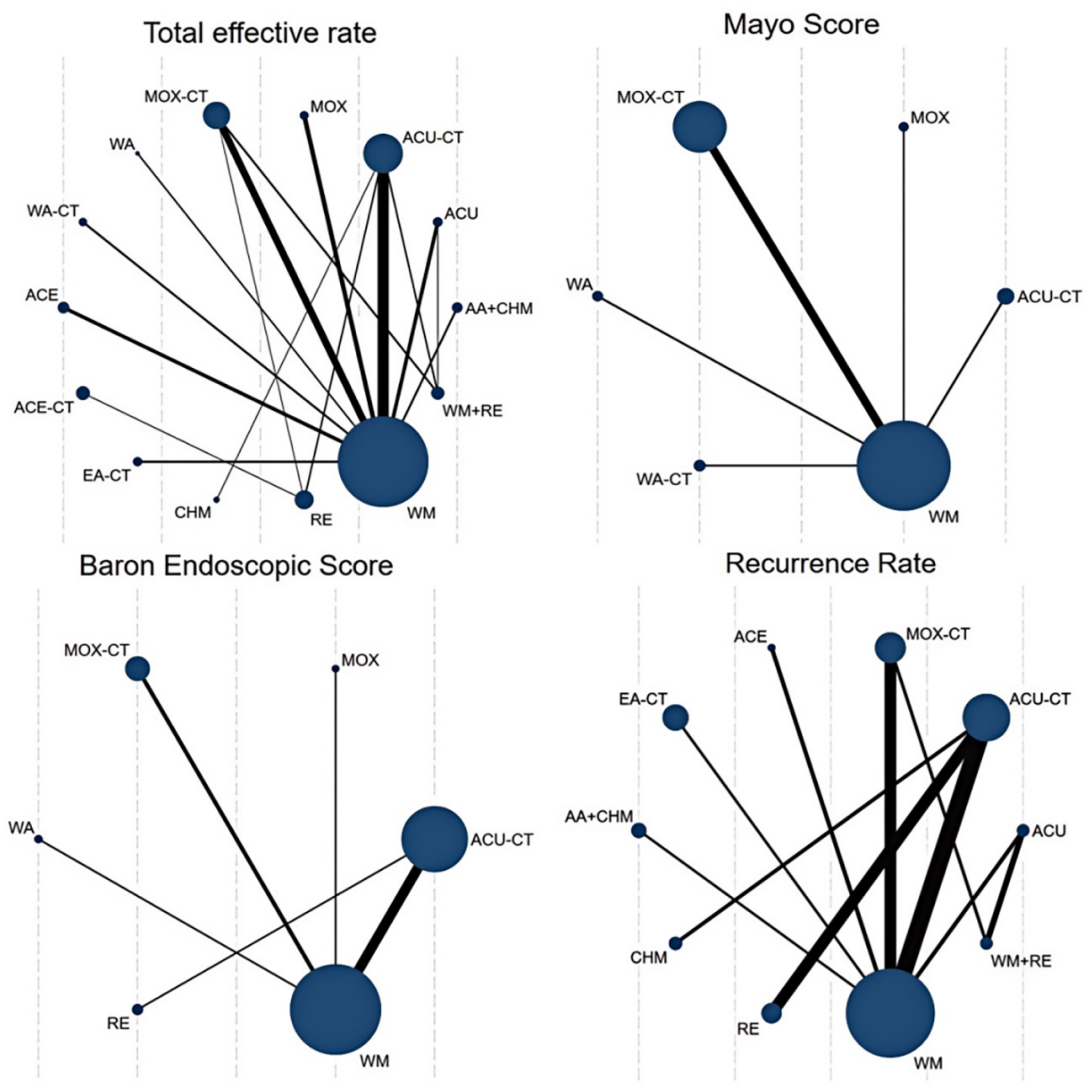
Network plot for different interventions. ACU, acupuncture; MOX, moxibustion; WA, warm acupuncture; ACE, acupoint catgut embedding; EA, Electroacupuncture; AA, auricular acupressure; CHM, Chinese Herbal Medicine; RE, retention enema; WM, western medicine; CT, combination therapy.

Among the 76 included RCTs, 75 studies reported total effective rate, involving 10 acupuncture-related therapies; 9 studies reported Mayo score, involving 5 acupuncture-related therapies; 12 studies reported Baron endoscopic score, involving 4 acupuncture-related therapies; 13 studies reported IL-6 levels, involving 5 acupuncture-related therapies; 18 studies reported TNF-α levels, involving 5 acupuncture-related therapies; and 15 studies reported recurrence rate, involving 6 acupuncture-related therapies. Node size reflects the number of participants, while the thickness of the connecting lines represents the number of randomized trials (see Figure 3 and Supplementary Material 6).

#### 3.4.2 Total effective rate

The total effective rate was defined as the proportion of patients achieving an “effective” or “improved” outcome. An “effective” outcome required the resolution of symptoms and an essentially normal mucosa on colonoscopy, whereas an “improved” outcome was defined by the presence of intermittent symptoms and only mild inflammatory changes.

**FIGURE 4:**
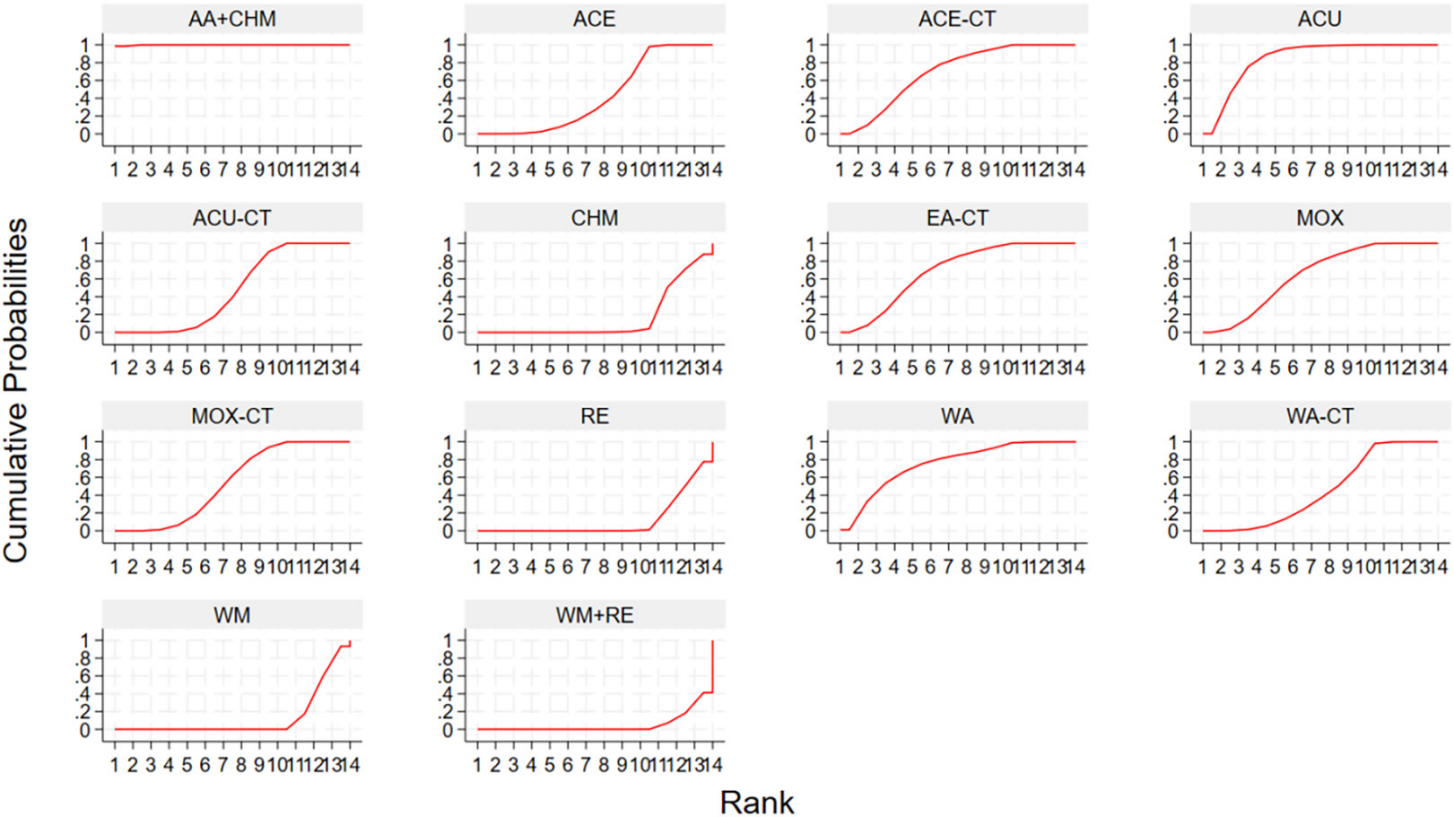
SUCRA plot for total effective rate. ACU, acupuncture; MOX, moxibustion; WA, warm acupuncture; ACE, acupoint catgut embedding; EA, Electroacupuncture; AA, auricular acupressure; CHM, Chinese Herbal Medicine; RE, retention enema; WM, western medicine; CT, combination therapy.

This network meta-analysis evaluated the effectiveness of various acupuncture-related interventions in improving clinical symptoms in patients. The results indicated that all ten acupuncture-related interventions significantly improved total effective rate compared with control groups. Among these, AA + CHM (RR 1.71, 95% CI: 1.47, 1.99, SUCRA = 99.9%) showed a relatively greater benefit, followed by ACU (RR 1.37, 95% CI: 1.22, 1.54, SUCRA = 84.8%) and WA (RR 1.34, 95% CI: 1.11, 1.61, SUCRA = 75.1%). Indirect comparisons among acupuncture-related therapies indicated that AA + CHM showed superior efficacy over all other interventions, and ACU was superior to ACU-CT (RR 1.12, 95% CI: 1.01, 1.24), MOX-CT (RR 1.11, 95% CI: 1.00, 1.23), WA-CT (RR 1.13, 95% CI: 1.00, 1.28), and ACE (RR 1.14, 95% CI: 1.02, 1.28) (Table 3). To explore the relative efficacy of each intervention, we calculated SUCRA values (Figure 4; Supplementary Material 7).

**TABLE 3:** League table of total effective rate.

AA+CHM													
1.25 (1.06, 1.46)	ACU												
1.40 (1.22, 1.60)	1.12 (1.01, 1.24)	ACU-CT											
1.34 (1.15, 1.57)	1.08 (0.95, 1.23)	0.96 (0.87, 1.06)	MOX										
1.38 (1.20, 1.59)	1.11 (1.00, 1.23)	0.99 (0.93, 1.05)	1.03 (0.93, 1.14)	MOX-CT									
1.28 (1.04, 1.58)	1.02 (0.85, 1.24)	0.91 (0.77, 1.08)	0.95 (0.79, 1.15)	0.92 (0.78, 1.10)	WA								
1.41 (1.21, 1.64)	1.13 (1.00, 1.28)	1.01 (0.92, 1.10)	1.05 (0.93, 1.18)	1.02 (0.93, 1.12)	1.11 (0.92, 1.33)	WA-CT							
1.42 (1.23, 1.65)	1.14 (1.02, 1.28)	1.02 (0.94, 1.10)	1.06 (0.95, 1.19)	1.03 (0.94, 1.12)	1.11 (0.93, 1.33)	1.01 (0.91, 1.12)	ACE						
1.32 (1.12, 1.56)	1.06 (0.92, 1.22)	0.95 (0.86, 1.04)	0.99 (0.86, 1.13)	0.96 (0.85, 1.07)	1.04 (0.85, 1.26)	0.94 (0.82, 1.07)	0.93 (0.82, 1.06)	ACE-CT					
1.33 (1.13, 1.56)	1.06 (0.93, 1.22)	0.95 (0.85, 1.05)	0.99 (0.87, 1.13)	0.96 (0.86, 1.07)	1.04 (0.86, 1.26)	0.94 (0.83, 1.06)	0.93 (0.83, 1.05)	1.00 (0.87, 1.16)	EA-CT				
1.62 (1.37, 1.90)	1.30 (1.13, 1.49)	1.15 (1.05, 1.27)	1.20 (1.05, 1.38)	1.17 (1.05, 1.31)	1.27 (1.04, 1.54)	1.15 (1.01, 1.30)	1.14 (1.01, 1.28)	1.22 (1.07, 1.40)	1.22 (1.06, 1.40)	CHM			
1.65 (1.41, 1.94)	1.33 (1.16, 1.51)	1.18 (1.09, 1.29)	1.23 (1.08, 1.40)	1.20 (1.08, 1.32)	1.29 (1.07, 1.56)	1.17 (1.04, 1.32)	1.16 (1.04, 1.30)	1.25 (1.18, 1.32)	1.25 (1.09, 1.42)	1.02 (0.90, 1.16)	RE		
1.65 (1.45, 1.88)	1.32 (1.20, 1.45)	1.18 (1.13, 1.22)	1.23 (1.12, 1.34)	1.19 (1.13, 1.25)	1.29 (1.09, 1.52)	1.17 (1.08, 1.26)	1.16 (1.08, 1.24)	1.24 (1.12, 1.38)	1.24 (1.13, 1.37)	1.02 (0.92, 1.13)	1.00 (0.91, 1.09)	WM	
1.71 (1.47, 1.99)	1.37 (1.22, 1.54)	1.22 (1.12, 1.33)	1.27 (1.13, 1.44)	1.24 (1.15, 1.33)	1.34 (1.11, 1.61)	1.21 (1.08, 1.36)	1.20 (1.08, 1.34)	1.29 (1.13, 1.47)	1.29 (1.14, 1.46)	1.06 (0.93, 1.20)	1.03 (0.92, 1.16)	1.04 (0.96, 1.13)	WM+RE

CU, acupuncture; MOX, moxibustion; WA, warm acupuncture; ACE, acupoint catgut embedding; EA, electroacupuncture; AA, auricular acupressure; CHM, Chinese Herbal Medicine; RE, retention enema; WM, western medicine; CT, combination therapy. Cells highlighted in blue represent comparisons in which the row intervention is significantly more effective than the column intervention. For instance, 1.25 (1.06, 1.46) indicates that AA + CHM significantly outperforms ACU.

#### 3.4.3 Mayo score

**FIGURE 5:**
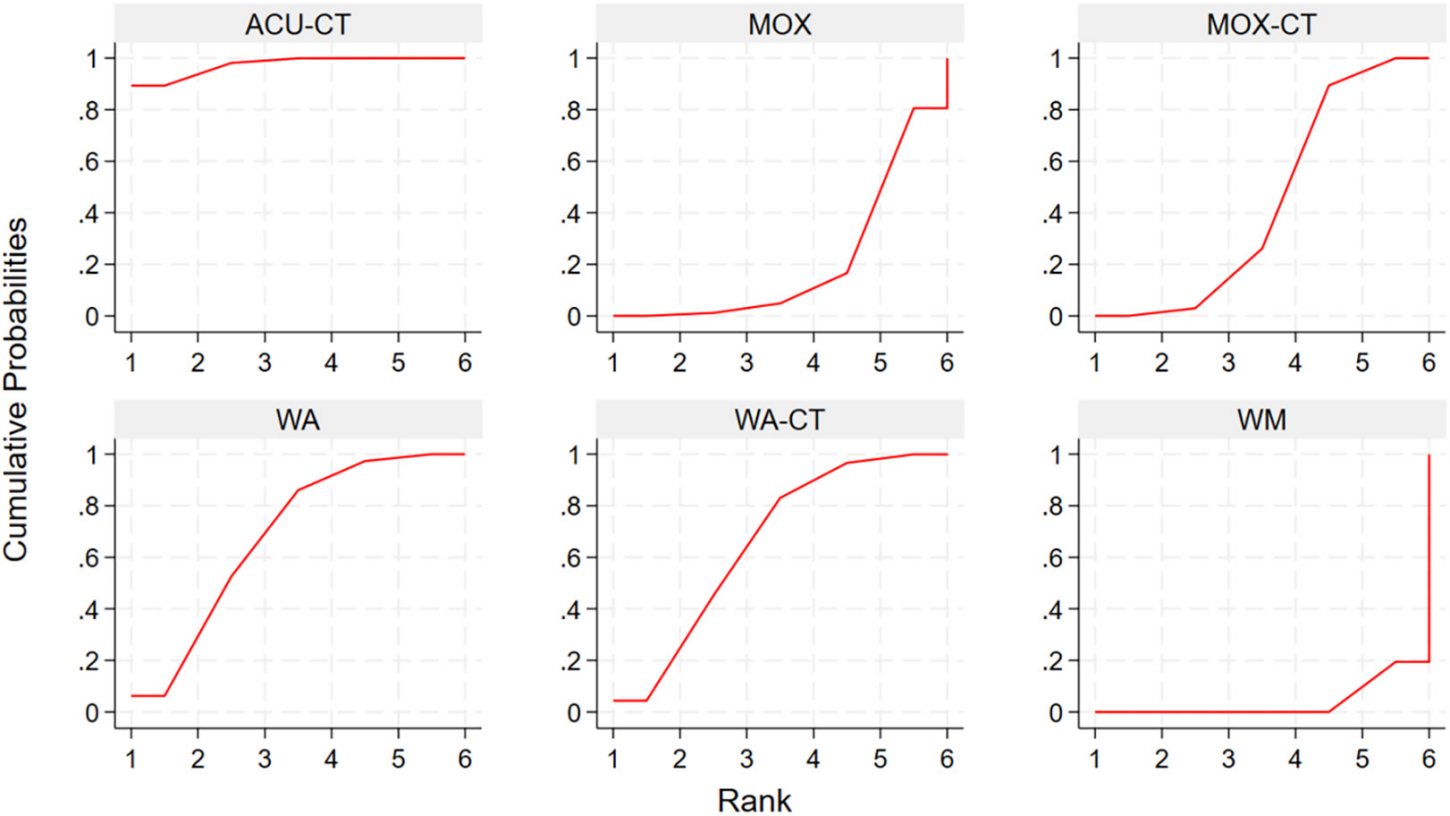
SUCRA plot for Mayo score. ACU, acupuncture; MOX, moxibustion; WA, warm acupuncture; WM, western medicine; CT, combination therapy.

Network meta-analysis results based on Mayo score showed that ACU-CT (SMD-4.85, 95% CI: –6.66, –3.05, SUCRA = 97.5%) was associated with the greatest reduction in Mayo score compared with WM, followed by WA (SMD-2.89, 95% CI: –4.68, –1.09, SUCRA = 68.4%), WA-CT (SMD-2.79, 95% CI: –4.57, –1.02, SUCRA = 65.8%), and MOX-CT (SMD-1.82, 95% CI: –2.60, –1.04, SUCRA = 43.7%). Indirect comparisons among five acupuncture-related therapies, ACU-CT was more effective than MOX (SMD-4.14, 95% CI: –6.65, –1.63) and MOX-CT (SMD-3.04, 95% CI: –5.00, –1.07) (Table 4). To explore the relative efficacy of each intervention, we calculated SUCRA values (Figure 5; Supplementary Material 7).

**TABLE 4:** League table of Mayo score.

ACU-CT					
−4.14 (−6.65, −1.63)	MOX				
−3.04 (−5.00, −1.07)	1.10 (−0.80, 3.01)	MOX-CT			
−1.97 (−4.52, 0.58)	2.17 (−0.33, 4.67)	1.07 (−0.89, 3.03)	WA		
−2.06 (−4.59, 0.47)	2.08 (−0.40, 4.56)	0.98 (−0.96, 2.92)	−0.09 (−2.62, 2.43)	WA-CT	
−4.85 (−6.66, −3.05)	−0.71 (−2.45, 1.02)	−1.82 (−2.60, −1.04)	−2.89 (−4.68, −1.09)	−2.79 (−4.57, −1.02)	WM

ACU, acupuncture; MOX, moxibustion; WA, warm acupuncture; WM, western medicine; CT, combination therapy. Cells highlighted in blue represent comparisons in which the row intervention is significantly more effective than the column intervention. For instance, –4.14 (−6.65,–1.63) indicates that ACU-CT significantly outperforms MOX.

#### 3.4.4 Baron endoscopy score

**FIGURE 6:**
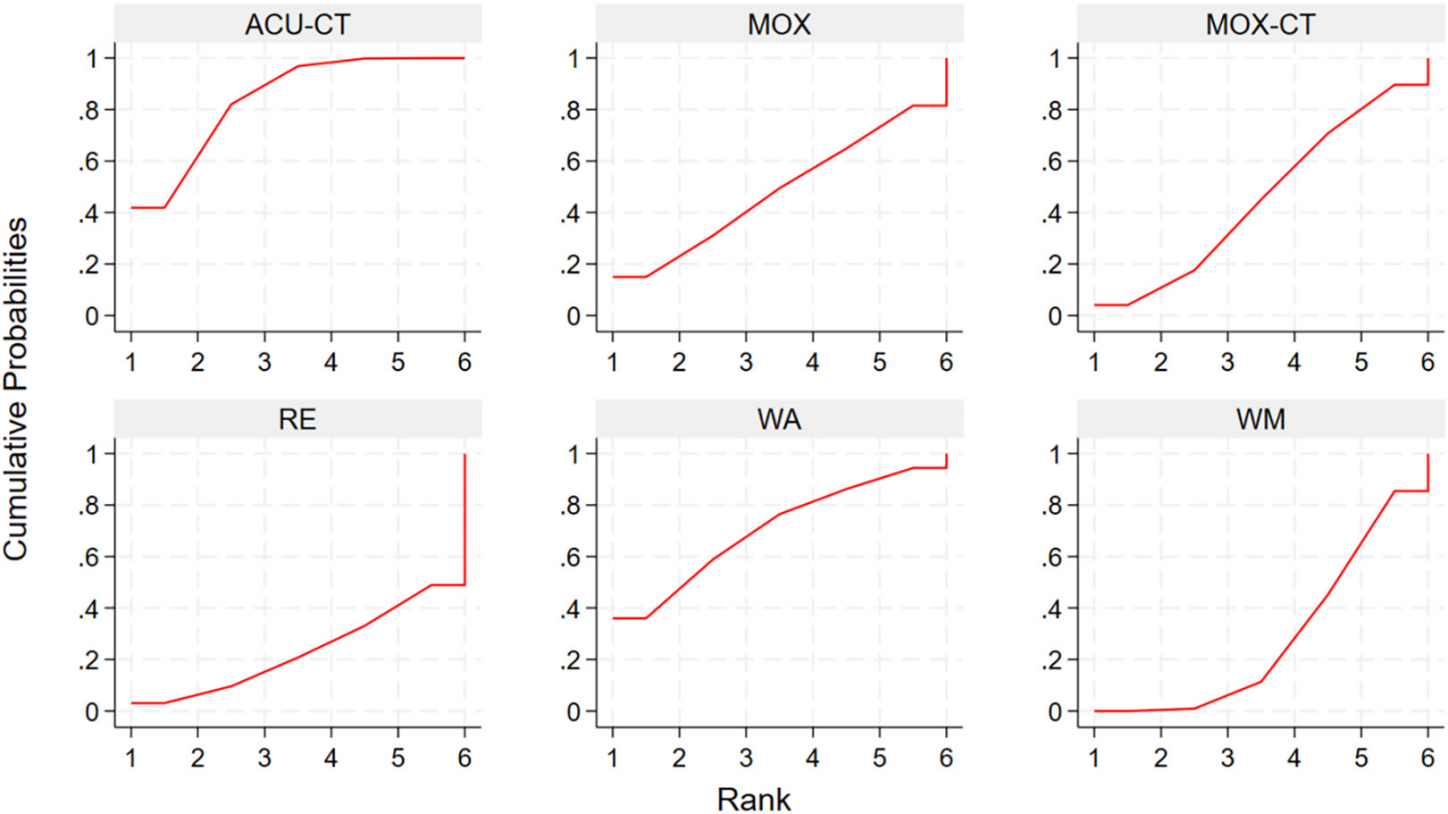
SUCRA plot for Baron endoscopy score. ACU, acupuncture; MOX, moxibustion; WA, warm acupuncture; RE, retention enema; WM, western medicine; CT, combination therapy.

Network meta-analysis results based on baron endoscopy score indicated that only ACU-CT led to a statistically significant reduction compared with WM (SMD-2.31, 95% CI: –3.81, –0.81, SUCRA = 84.1%). Other interventions showed a downward trend, but without statistical significance (Table 5). To explore the relative efficacy of each intervention, we calculated SUCRA values (Figure 6; Supplementary Material 7).

**TABLE 5:** League table of Baron endoscopy score.

ACU-CT					
−1.60 (−5.56, 2.37)	MOX				
−1.70 (−4.29, 0.89)	−0.10 (−4.34, 4.13)	MOX-CT			
−0.35 (−4.32, 3.63)	1.25 (−3.94, 6.44)	1.36 (−2.88, 5.59)	WA		
−3.06 (−6.74, 0.61)	−1.47 (−6.87, 3.94)	−1.36 (−5.86, 3.14)	−2.72 (−8.13, 2.70)	RE	
−2.31 (−3.81, −0.81)	−0.71 (−4.38, 2.95)	−0.61 (−2.72, 1.50)	−1.96 (−5.64, 1.71)	0.75 (−3.22, 4.72)	WM

ACU, acupuncture; MOX, moxibustion; WA, warm acupuncture; RE, retention enema; WM, western medicine; CT, combination therapy. Cells highlighted in blue represent comparisons in which the row intervention is significantly more effective than the column intervention. For instance, –2.31 (−-3.81, –0.81) indicates that ACU-CT significantly outperforms WM.

#### 3.4.5 IL-6

Network meta-analysis results based on IL-6 showed that among all interventions compared with WM, WA exhibited the most significant reduction (SMD-3.10, 95% CI: –4.56, –1.65, SUCRA = 96.1%), followed by ACE (SMD-2.13, 95% CI: –3.49, –0.77, SUCRA = 77%), WA-CT (SMD-1.76, 95% CI: –2.54, –0.98, SUCRA = 69.2%), and MOX-CT (SMD-1.18, 95% CI: –1.85, –0.50, SUCRA = 49%). Compared with WM + RE, WA (SMD-2.85, 95% CI: –4.71, –0.99, SUCRA = 96.1%), WA-CT (SMD-1.51, 95% CI: –2.90, –0.11, SUCRA = 69.2%), and ACE (SMD-1.88, 95% CI: –3.66, –0.09, SUCRA = 77%) also demonstrated significantly greater reductions. In indirect comparisons, WA was superior to ACU-CT (SMD 2.24, 95% CI: 0.51, 3.98) and MOX-CT (SMD 1.93, 95% CI: 0.32, 3.54). Other comparisons showed a trend of improvement but without statistical significance (Supplementary Material 8). To explore the relative efficacy of each intervention, we calculated SUCRA values (Supplementary Material 7).

#### 3.4.6 TNF-α

The network meta-analysis showed that WA-CT was the most effective intervention in reducing TNF-α levels compared with WM (SMD-2.32, 95% CI: –4.54, –0.10, SUCRA = 76.8%), followed by MOX-CT (SMD-2.00, 95% CI: –3.19, –0.81, SUCRA = 71.7%) and ACU-CT (SMD-1.64, 95% CI: –3.04, –0.24, SUCRA = 61.4%). However, no statistically significant differences were observed among the acupuncture-related interventions (Supplementary Material 8). To explore the relative efficacy of each intervention, we calculated SUCRA values (Supplementary Material 7).

#### 3.4.7 Recurrence rate

For recurrence rate, ACU significantly reduced recurrence compared with WM (OR 0.12, 95% CI: 0.03, 0.43, SUCRA = 89.3%), followed by ACE (OR 0.17, 95% CI: 0.04, 0.77, SUCRA = 78.1%), ACU-CT (OR 0.21, 95% CI: 0.08, 0.57, SUCRA = 76.6%), and MOX-CT (OR 0.21, 95% CI: 0.10, 0.44, SUCRA = 75.4%). Compared with WM + RE, ACU (OR 0.15, 95% CI: 0.03, 0.65, SUCRA = 89.3%) and MOX-CT (OR 0.26, 95% CI: 0.09, 0.76, SUCRA = 75.4%) also showed superior efficacy. Compared with RE, only ACU-CT (OR 0.23, 95% CI: 0.06, 0.84, SUCRA = 76.6%) showed a statistically significant advantage. No significant differences were found among acupuncture-related interventions (Supplementary Material 8). To explore the relative efficacy of each intervention, we calculated SUCRA values (Supplementary Material 7).

### 3.5 Adverse events

It is worth noting that there were 13 RCTs reported adverse events, involving 125 patients. Among them, 88 adverse events occurred in the control groups and 37 in the treatment groups (Supplementary Material 9). There were no serious adverse effects in any of the studies. The available data suggest that acupuncture-related therapies are associated with a lower incidence and severity of adverse events compared to control group, indicating a potentially better safety profile.

### 3.6 Publication bias

The potential for publication bias was assessed through visual inspection of funnel plots for all outcomes. The funnel plots for the total effective rate and recurrence rate presented a generally symmetrical appearance, suggesting a low likelihood of publication bias. Similarly, the distributions for IL-6 and TNF-α also showed a broadly symmetrical pattern, indicating that publication bias for these inflammatory markers was not prominent. In contrast, the funnel plots for the Mayo score and Baron endoscopy score displayed some degree of asymmetry. This observed asymmetry may point to the possible presence of publication bias or reflect underlying clinical and methodological heterogeneity among the included studies for these specific outcomes. Overall, while minor asymmetries were present for some measures, the funnel plot analysis did not provide clear evidence of substantial publication bias across the network meta-analysis. See Supplementary Material 10 for details.

### 3.7 Quality of evidence assessment

The overall certainty of evidence for the outcomes evaluated was moderate to low. Total effective rate was graded as moderate certainty, primarily limited by a serious risk of bias across studies due to inadequate reporting of randomization and allocation concealment, as well as the inherent challenges in blinding participants and practitioners. For Mayo score, Baron endoscopy score, and adverse events, the certainty was low. These ratings were influenced by serious risks of bias, considerable heterogeneity, and imprecision resulting from limited sample sizes and wide confidence intervals. Additionally, potential publication bias could not be excluded, given the predominance of positive results and regional concentration of studies (see Table 6 for details).

**TABLE 6:** Assessment of quality of evidence.

Outcome	Participants (studies)	Risk of bias	Inconsistency	Indirectness	Imprecision	Publication bias	Certainty (GRADE)
Total effective rate	7,404 (75 RCTs)	Serious ➀	Not serious	Not serious	Not serious	Possible ➁	⊕⊕⊕○ Moderate
Mayo score	734 (9 RCTs)	Serious ➀	Serious ➁	Not serious	Serious ➃	Possible ➁	⊕⊕○○ Low
Baron endoscopy score	1,124 (12 RCTs)	Serious ➀	Serious ➄	Not serious	Serious ➃	Possible ➁	⊕⊕○○ Low
Adverse events	1,236 (13 RCTs)	Serious ➅	Not serious	Not serious	Serious ➃	Possible ➁	⊕⊕○○ Low

➀ The majority of the included trials only mentioned “randomization” without describing the specific random allocation sequence generation or allocation concealment methods. Due to the nature of the acupuncture interventions, performance bias was likely high as blinding of practitioners and participants was not feasible. Many trials also did not report blinding of outcome assessors. ➁ Publication bias cannot be ruled out, as the funnel plot’s symmetry may be misleading given the field’s propensity for positive results and the overwhelming predominance of Chinese studies, introducing potential language and geographic bias. ➂ Considerable heterogeneity (τ^2^ = 0.71) was observed, reflecting inconsistent effects on the Mayo score across the included trials. ➃ The total sample size for this outcome is limited, resulting in a wide 95% confidence interval for the pooled effect estimate and imprecise results. ➄ The moderate-to-high heterogeneity (τ^2^ = 0.343) reflects substantial inconsistency across studies. ➅ Most studies did not systematically monitor or report adverse events, leading to an underestimation of their incidence.

## 4 Discussion

### 4.1 Principal findings

This systematic review and network meta-analysis provides a comprehensive hierarchy of the relative efficacy and safety of various acupuncture-related therapies for UC, based on 76 RCTs. Our findings suggest several potentially important clinical implications:

1. For total effective rate: AA + CHM demonstrated the highest probability of being the best intervention. This highlights the potent synergistic effect that can be achieved by combining internal herbal medicine with external acupoint stimulation.
2. For promoting mucosal healing: Baron score assesses the extent of UC disease, and Mayo score assesses mucosal healing. Intestinal mucosal healing is considered to be a key goal of UC treatment, which is closely related to reducing the recurrence rate, reducing the risk of colectomy, and preventing colorectal cancer (107). ACU-CT was significantly better than the control group in improving colonoscopic scores, indicating that acupuncture combined therapy could promote the healing of the damaged intestinal mucosa and control the progression of the disease.
3. For reducing systemic inflammation: TNF-α and IL-6 are key cytokines involved in inflammatory and autoimmune processes and their overexpression is closely related to inflammatory damage to the intestinal mucosa (108). Our quantitative findings provide specific support for the anti-inflammatory potential of acupuncture therapies. The results showed that WA and WA-CT were the most effective interventions for lowering key pro-inflammatory cytokines IL-6 and TNF-α, respectively. This indicates that the thermal stimulation characteristic of warm acupuncture may have a specific and powerful anti-inflammatory effect, modulating the underlying immune dysregulation in UC.
4. For preventing disease relapse: ACU alone showed the greatest advantage in reducing recurrence rates. This remarkable finding suggests that conventional acupuncture may possess long-term regulatory effects that help sustain remission, potentially by restoring physiological balance and regulating the gut-brain axis.
5. Safety profile: The available data on adverse events consistently indicated that acupuncture-related therapies were associated with a lower incidence and severity of adverse events compared to conventional pharmaceutical treatments. This favorable safety profile positions these therapies as valuable complementary or alternative options, especially for patients intolerant to or seeking to reduce the burden of long-term drug use.

Acupoint analysis revealed that three studies using auricular acupressure all selected Spleen (MA-IC 3), Large Intestine (MA-IC 5), Endocrine (MA-AT 1), Sympathetic (MA-AH 7), and Subcortex (MA-AT 4). 2 studies using scalp acupuncture were used in both the stomach zone and intestinal zone. High-frequency acupoint analysis showed that Tianshu (ST25), Zusanli (ST36), Shangjuxu (ST37), and Zhongwan (CV12) were the most commonly used points. Tianshu is the Front-Mu point of the large intestine, which has the effect of regulating the gastrointestinal function and restoring normal intestinal motility and transformation. It is frequently used to relieve symptoms of bowel dysfunction such as abdominal pain, diarrhea, and constipation in UC patients, and is also a commonly selected point in experimental animal models of acupuncture for UC (109, 110). Therefore, the improvements in Mayo score and endoscopic outcomes observed in our study may be mechanistically underpinned by the widespread use of these pivotal acupoints, which are believed to exert multi-target effects. Zusanli is the Lower He-Sea point of the stomach, is known for harmonizing the stomach and intestines, regulating Qi of the Yangming meridian, enhancing immunity, and improving systemic function. Shangjuxu is the Lower He-Sea point of the large intestine, which has the effect of regulating the stomach and intestines, regulating qi and dissolving stagnation. Zhongwan is the Front-Mu point of the stomach, which has the effect of soothing the middle coke qi machine, and reducing stomach inversion and relieving pain. Acupuncture can regulate immune balance, reduce the expression of pro-inflammatory factors, and repair the intestinal mucosal barrier function, so it is effective for UC (111, 112).

The differences in the therapeutic efficacy of various acupuncture-related therapies for UC may be attributed to their distinct stimulation modalities, mechanisms of action, and intervention characteristics. Traditional manual acupuncture uses physical stimulation on the body surface as a method to regulate the immune response by activating the three anti-inflammatory pathways, including the cholinergic anti-inflammatory pathway, splenic sympathetic anti-inflammatory pathway, and the hypothalamic–pituitary–adrenal (HPA) axis (113). Electroacupuncture combines millineedle stimulation with electrical stimulation to enhance the amount of stimulation to acupuncture points, which can jointly stimulate the vagus nerve, activate the cholinergic anti-inflammatory pathway, and inhibit the expression of pro-inflammatory factors, thereby alleviating the inflammatory response of UC (114). Moxibustion stimulates meridian acupuncture points with warmth, strengthens the flow of qi and blood in the body, and regulates the intestinal flora by inhibiting the expression of IL-12 and TNF-α (115). Acupoint catgut embedding involves implanting absorbable sutures into specific acupoints, delivering prolonged and gentle stimulation. This method has been reported to support intestinal microecological balance and immune regulation, offering long-lasting and stable clinical efficacy (116). Auricular acupressure stimulates corresponding reflex points related to the affected internal organs, thereby promoting intestinal peristalsis and enhancing gastrointestinal function. Given the unique features of each intervention in stimulation method, intensity, and therapeutic mechanisms, personalized treatment approaches tailored to patients’ physical constitution and clinical presentation are warranted in future practice.

### 4.2 Strengths

This study has several notable strengths: (1) The literature search system is comprehensive, the sample size included is sufficient, and the variety of therapies is covered, which enhances the representativeness and stability of the conclusions; (2) The network meta-analysis method was used to achieve direct and indirect comparison between a variety of acupuncture-related therapies, thereby overcoming the limitations of traditional pairwise meta-analysis that are restricted to two-group comparisons. (3) Outcomes included symptom scores, endoscopic scores, serum inflammatory markers and recurrence rate, allowing for a multidimensional evaluation of therapeutic efficacy. (4) Acupuncture-related interventions demonstrated a low incidence of adverse events, indicating a favorable safety profile, highlighting the advantages of the holistic regulatory approach of traditional Chinese medicine.

### 4.3 Limitations

This study has several limitations: (1) Among the 76 RCTs, some studies only mentioned “randomization” without providing specific details on the randomization method. Moreover, most RCTs did not report blinding procedures or allocation concealment, which may affect the credibility of the risk of bias assessments. (2) Only a limited number of studies reported Baron endoscopic score and Mayo score, which may compromise the stability and generalizability of the analyses. (3) The majority of the included studies were conducted in China, introducing a potential regional bias. Therefore, the international applicability of the findings requires further validation. (4) Due to limitations in the original literature, interventions involving combination therapies (one of which is acupuncture-related therapy) were uniformly categorized as combination therapy, including ACU + MOX + WM, ACU + MOX + CHM, ACU + MOX + RE, etc. Given the diversity of combination therapy strategies, further clarification will be provided in future studies. (5) A small number of included studies did not fully report the details of their treatment schedules. While this does not undermine the primary findings of our network meta-analysis regarding the comparative efficacy of different therapy classes, it highlights a key area for improvement in future trial reporting to enhance reproducibility and clinical translation.

## 5 Conclusion

ACU, MOX, WA, and their combination therapies may represent the most effective treatment for UC. The identified core acupoints provide a standardized basis for clinical practice. The results of this analysis suggest that acupuncture has the potential to serve as a component of the comprehensive management strategy for UC and is expected to gain broader clinical application in the future. Future research should focus on conducting large-sample, high-quality RCTs with longer follow-up periods to confirm the long-term efficacy and to elucidate the specific mechanisms of action behind these distinct acupuncture interventions.

## Data Availability

The original contributions presented in the study are included in the article/Supplementary Material, further inquiries can be directed to the corresponding author.
