# Supplementary material for: Comparative clinical efficacy of acupuncture-related therapies for ulcerative colitis: a systematic review and network meta-analysis
Source: Front Med (Lausanne). 2025 Dec 12;12:1676608. doi: 10.3389/fmed.2025.1676608 (PMC12741068; doi:10.3389/fmed.2025.1676608)

Supplementary Appendix

Comparative Efficacy and Safety of Acupuncture-Related Therapies for Ulcerative Colitis: A Systematic Review and Network Meta-Analysis of Randomized Controlled Trials

Table of contents

Appendix 1: PRISMA NMA Checklist 2
Appendix 2: Search strategy 7
Appendix 3: Characteristics of included studies 8
Appendix 4: Risk of bias of randomized clinical trials 34
Appendix 5: Evaluation of inconsistency and heterogeneity 39
Appendix 6: Network maps of outcomes 40
Appendix 7: League Tables of Secondary Outcomes. 42
Appendix 8: SUCRA and cumulative probability plots 45
Appendix 9: Summary Table of Adverse Events Reported in Included Trials 51
Appendix 10: Funnel plots 52

Appendix 1: PRISMA NMA Checklist

Section/Topic	Item #	Checklist Item	Reported on Page #
**TITLE**			
Title	1	Identify the report as a systematic review *incorporating a network meta-analysis (or related form of meta-analysis).*	Title
**ABSTRACT**			
Structured summary	2	Provide a structured summary including, as applicable:<br>**Background:** main objectives<br>**Methods:** data sources; study eligibility criteria, participants, and interventions; study appraisal; and *synthesis methods, such as network meta-analysis.*<br>**Results:** number of studies and participants identified; summary estimates with corresponding confidence/credible intervals; *treatment rankings may also be discussed. Authors may choose to summarize pairwise comparisons against a chosen treatment included in their analyses for brevity.*<br>**Discussion/Conclusions:** limitations; conclusions and implications of findings.<br>**Other:** primary source of funding; systematic review registration number with registry name.	Abstract
**INTRODUCTION**			
Rationale	3	Describe the rationale for the review in the context of what is already known*, including mention of why a network meta-analysis has been conducted.*_	1 Introduction
Objectives	4	Provide an explicit statement of questions being addressed, with reference to participants, interventions, comparisons, outcomes, and study design (PICOS).	2.2 Inclusion and Exclusion Criteria

METHODS

Protocol and registration	5	Indicate whether a review protocol exists and if and where it can be accessed (e.g., Web address); and, if available, provide registration information, including registration number.	2 Methods
Eligibility criteria	6	Specify study characteristics (e.g., PICOS, length of follow-up) and report characteristics (e.g., years considered, language, publication status) used as criteria for eligibility, giving rationale. *Clearly describe eligible treatments included in the treatment network, and note whether any have been clustered or merged into the same node (with justification).*_	2.2 Inclusion and Exclusion Criteria
Information sources	7	Describe all information sources (e.g., databases with dates of coverage, contact with study authors to identify additional studies) in the search and date last searched.	2.1 Search strategy
Search	8	Present full electronic search strategy for at least one database, including any limits used, such that it could be repeated.	Appendix 2
Study selection	9	State the process for selecting studies (i.e., screening, eligibility, included in systematic review, and, if applicable, included in the meta-analysis).	2.3 Study selection and data extraction
Data collection process	10	Describe method of data extraction from reports (e.g., piloted forms, independently, in duplicate) and any processes for obtaining and confirming data from investigators.	2.3 Study selection and data extraction
Data items	11	List and define all variables for which data were sought (e.g., PICOS, funding sources) and any assumptions and simplifications made.	2.3 Study selection and data extraction
**Geometry of the network**	**S1**	Describe methods used to explore the geometry of the treatment network under study and potential biases related to it. This should include how the evidence base has been graphically summarized for presentation, and what characteristics were compiled and used to describe the evidence base to readers.	2.5 Certainty of Evidence
Risk of bias within individual studies	12	Describe methods used for assessing risk of bias of individual studies (including specification of whether this was done at the study or outcome level), and how this information is to be used in any data synthesis.	2.4 Quality assessment
Summary measures	13	State the principal summary measures (e.g., risk ratio, difference in means). *Also describe the use of*	2.6 Statistical Analysis

		additional summary measures assessed, such as treatment rankings and surface under the cumulative ranking curve (SUCRA) values, as well as modified approaches used to present summary findings from meta-analyses.	
Planned methods of analysis	14	Describe the methods of handling data and combining results of studies for each network meta-analysis. This should include, but not be limited to:<br>• *Handling of multi-arm trials;*<br>• *Selection of variance structure;*<br>• *Selection of prior distributions in Bayesian analyses; and*<br>• *Assessment of model fit.*	2.6 Statistical Analysis
**Assessment of Inconsistency**	**S2**	Describe the statistical methods used to evaluate the agreement of direct and indirect evidence in the treatment network(s) studied. Describe efforts taken to address its presence when found.	2.6 Statistical Analysis
Risk of bias across studies	15	Specify any assessment of risk of bias that may affect the cumulative evidence (e.g., publication bias, selective reporting within studies).	2.4 Quality assessment
Additional analyses	16	Describe methods of additional analyses if done, indicating which were pre-specified. This may include, but not be limited to, the following:<br>• Sensitivity or subgroup analyses;<br>• Meta-regression analyses;<br>• *Alternative formulations of the treatment network; and*<br>• *Use of alternative prior distributions for Bayesian analyses (if applicable).*_	2.6 Statistical Analysis

RESULTS†

Study selection	17	Give numbers of studies screened, assessed for eligibility, and included in the review, with reasons for exclusions at each stage, ideally with a flow diagram.	3.1 Literature Search Results
**Presentation of network structure**	**S3**	Provide a network graph of the included studies to enable visualization of the geometry of the treatment network.	3.1 Literature Search Results
**Summary of network geometry**	**S4**	Provide a brief overview of characteristics of the treatment network. This may include commentary on the abundance of trials and randomized patients for the different interventions and pairwise comparisons in the network, gaps of evidence in the treatment network, and potential biases reflected by the network structure.	3.4 Network Meta-Analysis Results
Study characteristics	18	For each study, present characteristics for which data were extracted (e.g., study size, PICOS, follow-up period) and provide the citations.	3.2 Included study characteristics
Risk of bias within studies	19	Present data on risk of bias of each study and, if available, any outcome level assessment.	3.3 Risk of bias, Certainty of Evidence, and Consistency
Results of individual studies	20	For all outcomes considered (benefits or harms), present, for each study: 1) simple summary data for each intervention group, and 2) effect estimates and confidence intervals. *Modified approaches may be needed to deal with information from larger networks.*	3.4 Network Meta-Analysis Results
Synthesis of results	21	Present results of each meta-analysis done, including confidence/credible intervals. *In larger networks, authors may focus on comparisons versus a particular comparator (e.g. placebo or standard care), with full findings presented in an appendix. League tables and forest plots may be considered to summarize pairwise comparisons.* If additional summary measures were explored (such as treatment rankings), these should also be presented.	3.4 Network Meta-Analysis Results and 3.6 Publication Bias
**Exploration for inconsistency**	**S5**	Describe results from investigations of inconsistency. This may include such information as measures of model fit to compare consistency and inconsistency models, *P* values from statistical tests, or summary of inconsistency estimates from different parts of the treatment network.	3.6 Publication Bias
Risk of bias across studies	22	Present results of any assessment of risk of bias across studies for the evidence base being studied.	3.7 Quality of Evidence Assessment

Results of additional analyses	23	Give results of additional analyses, if done (e.g., sensitivity or subgroup analyses, meta-regression analyses, *alternative network geometries studied, alternative choice of prior distributions for Bayesian analyses,* and so forth).	3.4 Network Meta-Analysis Results
**DISCUSSION**			
Summary of evidence	24	Summarize the main findings, including the strength of evidence for each main outcome; consider their relevance to key groups (e.g., healthcare providers, users, and policy-makers).	4.1 Principal Findings
Limitations	25	Discuss limitations at study and outcome level (e.g., risk of bias), and at review level (e.g., incomplete retrieval of identified research, reporting bias). *Comment on the validity of the assumptions, such as transitivity and consistency. Comment on any concerns regarding network geometry (e.g., avoidance of certain comparisons).*	4.3 Limitations
Conclusions	26	Provide a general interpretation of the results in the context of other evidence, and implications for future research.	5 Conclusion
**FUNDING**			
Funding	27	Describe sources of funding for the systematic review and other support (e.g., supply of data); role of funders for the systematic review. This should also include information regarding whether funding has been received from manufacturers of treatments in the network and/or whether some of the authors are content experts with professional conflicts of interest that could affect use of treatments in the network.	8 Funding

Appendix 2: Search strategy

Database	Search strategy
PubMed	#1 "Acupuncture"[MeSH Terms] OR "acupuncture therapy"[MeSH Terms] OR "Acupuncture"[Title/Abstract] OR "Pharmacopuncture"[Title/Abstract] OR "Electro-acupuncture"[Title/Abstract] OR "warm needle"[Title/Abstract] OR "fire needle"[Title/Abstract] OR "blood letting therapy"[Title/Abstract] OR "Moxibustion"[MeSH Terms] OR "Moxibustion"[Title/Abstract] OR ("Auricular"[All Fields] AND "application pressure"[Title/Abstract]) OR "auricular needle"[Title/Abstract] OR "acupoint catgut embedding"[Title/Abstract] OR "acupoint injection"[Title/Abstract]<br>#2 ulcerative colitis[MeSH Terms] OR ulcerative colitis[Title/Abstract] OR UC[Title/Abstract]<br>#3 #1 AND#2
Embase	（'ulcerative colitis'/exp OR 'ulcerative colitis':ti,ab) AND (acupuncture OR 'acupuncture therapy' OR pharmacopuncture OR 'electro-acupuncture' OR 'warm needle' OR 'fire needle' OR 'blood letting therapy' OR moxibustion OR 'auricular needle' OR 'acupoint catgut embedding' OR 'acupoint injection':ti,ab)<br>Limit to study types: Randomized Controlled Trial
Cochrane library	#1 MeSH descriptor: [Colitis, Ulcerative] explode all trees<br>#2 (ulcerative colitis OR UC):ti,ab,kw (Word variations have been searched)<br>#3 #1 OR #2<br>#4 MeSH descriptor: [Acupuncture] explode all trees<br>#5 (Acupuncture OR acupuncture therapy OR Acupuncture OR Pharmacopuncture OR Electro-acupuncture OR warm needle OR fire needle OR blood letting therapy OR Moxibustion OR Moxibustion OR auricular needle OR acupoint catgut embedding OR acupoint injection)<br>#6 #5 or #4<br>#7 #3 and #6
Web of science	(TS=("ulcerative colitis" OR "UC")) AND TS=("acupuncture" OR "acupuncture therapy" OR "pharmacopuncture" OR "electro-acupuncture" OR "warm needle" OR "fire needle" OR "blood letting therapy" OR "moxibustion" OR "auricular needle" OR "acupoint catgut embedding" OR "acupoint injection")

Appendix 3: Characteristics of included studies

Table S3.1: Baseline of characteristics of included studies

Study ID	Sample size (T/ C)	Age(Year,T/C)	Disease duration(Year,T/C)	Treatment Group	Control Group	Duration of treatment	Outcomes
Zhao 2012[1]	33/32	37.48±9.34<br>39.52±8.67	2.33±1.65<br>2.72±1.71	ACE+RE	RE	4 weeks	①
Zhang 2003[2]	43/35	39.5±9.25<br>39±19	8.25±3.88<br>8.25±3.88	ACU	WM+RE	4 weeks	①⑥
Wang 2022a[3]	67/67	44.27±3.65<br>44.15±3.57	2.61±0.35<br>2.57±0.43	ACU+CHM+WM	WM	4 weeks	①②
Cheng 2009[4]	40/40	53±8.5<br>53±8.5	5.84±2.58<br>5.84±2.58	MOX+CHM+WM	WM	4 weeks	①
Fan 2021[5]	45/45	41.55±5.56<br>42.38±4.15	4.32±1.03<br>4.67±1.15	MOX+CHM	WM	4 weeks	①③④⑤
Zhou 2008[6]	110/110	48.60±7.48<br>50.24±6.95	4.75±1.69<br>4.89±1.76	EA+MOX+WM	WM	6 weeks	①⑥
Xu 2022[7]	36/37	42.05±8.56<br>40.89±9.53	1.12±0.56<br>1.60±0.77	MOX+CHM	WM	4 weeks	①③⑤
Zhao 2019[8]	35/35	47.2±6.8<br>42.7±8.9	1.44±0.56<br>1.25±0.11	MOX	WM	12 weeks	①
Yang 2017[9]	30/30	41.17±7.43<br>42.25±6.81	4.13±1.26<br>3.84±1.47	MOX	WM	8 weeks	①②③

He 2021[10]	48/48	32.80±4.80<br>33.70±5.10	0.02±0.01<br>0.02±0.01	MOX+RE	WM	4 weeks	②③④
Zhao 2018[11]	55/55	46.25±10.12<br>45.37±12.46	2.45±1.31<br>2.83±1.72	ACU+CHM	WM	4 weeks	①⑤
Zhu 2003[12]	50/20	42.5±11.25<br>43±11	10.5±4.75<br>9.92±4.54	ACE	WM	4 weeks	①
Sun 2017[13]	36/36	43.53±9.24<br>44.14±9.47	6.44±2.55<br>6.47±2.51	MOX	WM	12 weeks	①
Ma 1997[14]	60/30	45.5±11.25<br>46.5±23.25	6.75±3.13<br>5.25±2.38	ACU	WM	4 weeks	①
Wang 2006[15]	30/30	40±6.5/<br>41±6.5	0.5~12<br>0.6~13	MOX	WM	4 weeks	①
Li 2006[16]	56/60	37.1<br>37.3	0.25~2.33<br>0.25~2	ACE	WM	4 weeks	①
Kang 2022[17]	50/50	40.02±9.11<br>39.27±9.80	3.77±1.84<br>3.65±2.41	ACU+CHM	WM	12 weeks	①②④⑤
Wu 2015[18]	52/50	49.5±7.6<br>48.5±7.3	NR<br>NR	ACU+MOX+CHM	CHM	8 weeks	①⑥
Zhang 2021a[19]	39/38	39.4±11.1<br>38.9±10.2	4.23±3.15<br>3.96±2.89	ACU	WM	8 weeks	①③
Gong 2020[20]	50/50	36.2±9.0<br>39.5±8.5	2.3±1.6<br>2.6±1.4	ACE	WM	6 weeks	①④⑤
Feng 2020[21]	36/36	31.54±3.48<br>32.22±3.97	5.36±1.83<br>5.21±2.13	ACU	WM	4 weeks	①

Zu 2017[22]	45/45	39.56±10.69<br>40.76±11.09	3.11±1.30<br>3.42±1.41	MOX+RE+WM	WM+RE	4 weeks	①④⑤
Lv 2017a[23]	50/50	40.86±10.83<br>41.09±10.68	3.19±1.2<br>3.27±1.28	MOX+CHM+WM	WM	4 weeks	①④⑤
Guo 2016[24]	35/35	46.25±15.11<br>50.2±14.19	5.08±2.1<br>4.83±1.74	ACU+MOX+WM	WM	6 weeks	①
Chen 2004[25]	100/30	44.5±13.25<br>42±11.5	11.13±5.44<br>10.13±4.84	ACE	WM	24 weeks	①
Chang 2017[26]	45/45	38.24±9.79<br>40.01±11.23	3.05±1.20<br>3.16±1.41	MOX+RE+WM	WM+RE	6 weeks	①④⑤
Wang 2020a[27]	32/32	27.24±2.23<br>26.12±5.09	5.77±1.99<br>5.34±2.12	EA+CHM+WM	WM	8 weeks	①
Xu 2009[28]	40/40	47.37±5.17<br>49.13±4.92	2.79±3.91<br>3.03±4.18	ACU+CHM	WM	6 weeks	①
Gu 2016[29]	38/37	40.5±2.8<br>42.5±3.2	4.9±1.3<br>5.2±2.12	MOX+CHM	WM	8 weeks	①⑥
Zhang 2022[30]	47/47	44±9<br>46±8	3.41±0.73<br>3.59±0.80	EA+WM	WM	8 weeks	①④
Xie 2019[31]	65/65	36±8<br>35±8	3.40±1.03<br>3.34±1.01	ACU+CHM	CHM	4 weeks	①⑤
Shen 2019[32]	50/50	37±9<br>38±10	0.9±0.1<br>0.8±0.1	MOX+WM	WM	12 weeks	①⑥
Shen 2012[33]	33/30	46±7<br>41±7	3.5±1.3<br>3.8±1.6	ACU	WM	12 weeks	①

Zong 2015[34]	34/32	43±12<br>42.5±11.25	1.05±0.275<br>3.55±1.225	ACE	WM	6 weeks	①⑥
Du 2008[35]	42/40	46±9.5<br>43.5±9.25	14.7±6.65<br>15.75±7.125	ACU+RE+CHM	WM+RE	4 weeks	①
Hou 2018[36]	48/48	35.76±4.53<br>36.27±4.36	4.69±1.21<br>4.73±1.17	MOX+WM	WM	4 weeks	①
Ye 2024[37]	60/60	39.49±4.28<br>39.44±4.38	3.19±0.42<br>3.23±0.39	ACU+CHM	WM	4 weeks	①
Wang 2022b[38]	30/30	47.22±4.02<br>47.42±4.03	4.55±1.29<br>4.49±1.37	ACU	WM	4 weeks	①
Du 2007[39]	89/80	43.6<br>41.7	4.8±1.4<br>4.7±2.1	AA+CHM	WM	4 weeks	①
Cong 2018[40]	75/72	38.61±11.74<br>37.82±12.67	3.75±2.81<br>3.61±2.47	ACU+CHM	WM	6 weeks	①②④⑤
Zhou 2003[41]	34/32	38.5±9.75<br>38.5±9.75	5±1.5<br>5±1.5	MOX	WM	4 weeks	①
Ge 2014[42]	31/31	35.6±7.5<br>38.4±7.8	3.7±2.8<br>4.0±2.5	EA+WM	WM	8 weeks	①
Zhang 2012[43]	45/45	37.63±8.71<br>35.27±8.56	6.32±3.11<br>6.95±3.23	ACU+RE	WM	4 weeks	①
Chen 2013[44]	40/40	47.5±12.25<br>46.5±13.75	8.5±3.25<br>9.5±4.25	MOX+RE+WM	WM+RE	4 weeks	①⑥
Xu 2023[45]	50/51	39.54±4.52<br>39.66±4.49	1.99±0.47<br>1.96±0.50	ACU+CHM+RE	RE	4 weeks	①②⑥

Wang 2020b[46]	36/36	35.0±3.2<br>34.8±2.9	7.0±2.1<br>6.9±1.8	ACU+CHM+WM	WM	6 weeks	①
Teng 2014[47]	40/40	38.5±8.75<br>39.5±8.75	5.2±2.4<br>5.75±2.625	EA+RE+WM	WM	8 weeks	①
Zhang 2009[48]	50/40	40.73±8.1<br>38.73±7.5	4.2±1.2<br>4.1±1.7	ACU+CHM	WM	4 weeks	①⑥
Cao 2001[49]	40/40	50±14<br>47.5±13.75	4.21±1.90<br>4.25±1.875	ACU+RE	WM+RE	6 weeks	①
Bao 2014[50]	50/50	41. 2±6. 7<br>42. 40. 3±5. 7	4. 3±1. 2<br>4. 5±1. 6	ACU+RE	WM	4 weeks	①
He 2015[51]	30/30	43.5±9.8<br>44.2±10.2	4.1±2.3<br>4.2±1.9	ACU+WM	WM	4 weeks	①⑤
Hui 2012[52]	60/60	49.63±6.12<br>48.65±5.11	3.54±3.96<br>3.34±4.21	MOX+RE	WM	8 weeks	①⑥
Lu 2021[53]	40/40	38.12±8.28<br>37.57±7.65	5.25±3.50<br>4.77±3.26	MOX+CHM	WM	4 weeks	①②③⑤
Wang 2010[54]	45/45	46.5±11.75<br>46.5±11.75	6.25±2.88<br>6.25±2.88	ACU+RE	WM	3 weeks	①
Li 2016[55]	30/30	43.18±11.63<br>44.82±2.52	NR<br>4.36±2.52	WA	WM	8 weeks	①
Zhang 2020[56]	34/34	39.3±10.1<br>38.5±9.9	4.21±3.03<br>3.96±2.61	WA	WM	8 weeks	①②③④
Wang 2020c[57]	40/40	38.3±4.3<br>38.2±4.1	2.4±0.6<br>2.3±0.3	ACU+CHM+WM	WM	4 weeks	①②

Wang 2008[58]	80/78	44.5±13.25	19±8	MOX+CHM	WM+RE	6 weeks	①
		44.5±12.25	17.75±7.625				
Lv 2017b[59]	46/46	39.78±10.58	3.12±1.31	MOX+RE+WM	WM	6 weeks	①④⑤
		40.47±11.36	3.26±1.39				
Jia 2015[60]	32/32	44±8	4.5±1.75	MOX+WM	WM	8 weeks	①
		40.5±8.25	4±1.5				
Zhang 2021b[61]	52/50	32.05±5.08	3.52±1.23	WA+CHM+WM	WM	8 weeks	①④⑤
		32.12±5.10	3.28±1.01				
Zheng 2020[62]	40/40	42.63±10.45	11.46±3.52	MOX+RE	WM	4 weeks	①②⑤
		46.70±10.70	10.63±2.95				
Li 2024[63]	30/30	34.27±8.33	2.42±1.19	ACU+WM	WM	8 weeks	①②⑥
		32.07±8.92	3.01±1.13				
Xiu 2020[64]	36/36	36.10±3.11	2.29±0.79	MOX+CHM+WM	WM	8 weeks	①③⑤
		35.92±3.13	2.30±0.75				
Zhou 2021[65]	47/46	36.52±6.24	8.32±1.16	WA+CHM+WM	WM	4 weeks	①④⑤
		35.24±5.45	8.05±1.12				
Hua 2022[66]	59/59	47.06±4.10	2.68±0.29	ACU+CHM+WM	WM	12 weeks	①②③⑤
		47.11±3.25	2.74±0.35				
Wang 2013[67]	45/45	43.65±13.58	NR	ACE	WM	4 weeks	①
		44.56±15.01	NR				
Han 2009[68]	50/40	40.73±8.1	4.2±1.2	ACU	WM	4 weeks	①⑥
		38.73±7.5	4.1±1.7				
Du 2008a[69]	89/80	43.61±10.16	4.5±1.4	AA+CHM	WM	4 weeks	①⑥
		41.76±1.3	4.1±1.2				

Zhang 2010[70]	40/40	42.64±6.91<br>38.73±7.52	3.91±2.3<br>4.12±1.73	ACU+WM	WM	6 weeks	①⑥
Du 2008b[71]	89/80	44.5±11.75<br>41.5±11.75	4.3±1.4<br>4.7±1.4	AA+CHM	WM	4 weeks	①
Lin 2023[72]	30/33	48±11<br>47.5±11.25	17.5±8.25<br>15.15±7.43	MOX	WM	12 weeks	①
Wang 2012[73]	40/30	44±3.27<br>42±3.31	7.75±3.63<br>7.71±3.65	MOX+RE	RE	7 weeks	①
Duan 2012[74]	320/320	45.5±14.75<br>46±14.5	9.63±4.69<br>9.63±4.69	ACE+RE	RE	8 weeks	①
He 2001[75]	36/35	41.5±13.25<br>41.5±13.25	10.09±4.96<br>10.09±4.96	ACU+RE	RE	4 weeks	①
Li 2008[76]	32/31	34.6<br>36.5	6.13±2.94<br>5.75±2.63	ACU+MOX+RE	RE	4 weeks	①⑥

Abbreviations:T, treatment group; C, control group; NR, not report;ACU,acupuncture;MOX,moxibustion;WA,warm acupuncture;ACE,acupoint catgut embedding;EA,electroacupuncture;AA,auricular acupressure;CHM,Chinese Herbal Medicine;RE,retention enema;WM,western medicine. ①Total effective rate; ②Baron endoscopy score; ③Mayo score; ④IL-6; ⑤TNF-α;⑥Recurrence rate.

References

1 Hongbo, Z. *et al.* Clinical Observation of 33 Cases of Ulcerative Colitis Treated with Jiawei Baitouweng Decoction for Enteral Drip Combined with Acupoint Catgut Embedding. *Journal of Traditional Chinese Medicine* **53**, 2112-2114+2121, doi:10.13288/j.11-2166/r.2012.24.012 (2012).

2 Yue, Z. & Zhenhui, Y. Acupuncture at Jiaji Points Combined with Plum-Blossom Needle Tapping for Treating 43 Cases of Ulcerative Colitis. *Journal of Traditional Chinese Medicine* **44** (2003).

3 Defang, W., Ming, Z. & Xiaoyan, L. Observation on the Efficacy of Acupuncture Combined with Modified Scutellaria Decoction in Treating Active Ulcerative Colitis (Dampness-Heat Accumulation Syndrome). *Guide Journal of Traditional Chinese Medicine and Pharmacology* **28** (2022).

4 Min, C. Clinical Observation of 40 Cases of Ulcerative Colitis Treated with Meridian-Focused Moxibustion Combined with Wenzhong Guyuan Decoction. *Journal of Traditional Chinese Medicine* **15**, 49-50 (2009).

5 Puyu, F. & Ning, W. The effect of Baitouweng Decoction combined with penetrating moxibustion on inflammatory cytokines and immune function in patients with damp-heat type ulcerative colitis. *Journal of Traditional Chinese Medicine* **36**, 1335-1338, doi:10.16368/j.issn.1674-8999.2021.06.280 (2021).

6 Guoying, Z. & Jianhong, J. Clinical Observation of Electroacupuncture Combined with Moxibustion and Medication in the Treatment of Ulcerative Colitis. *Journal of Traditional Chinese Medicine*, 2069-2071, doi:10.13193/j.archtcm.2008.09.230.zhougy.088 (2008).

7 Baocai, X. & Wei, C. Evaluation of the Effect of Lei's Silver Cup Moxibustion with Ginger Partition Combined with the Fire-Reinforcing Earth-Generating Method on Ulcerative Colitis in Remission. *Chinese General Practice* **20**, 844-847+902, doi:10.16766/j.cnki.issn.1674-4152.002469 (2022).

8 Wenwen, Z. *et al.* Bioinformatics study on the mechanism of umbilical moxibustion therapy on the BTNL2-HLA signaling pathway in patients with ulcerative colitis. *Chinese Journal of Information on Traditional Chinese Medicine* **26** (2019).

9 Zhouyu, Y., Dingyan, B., Zhan, Y. & Yongheng, H. Clinical Study on Herbal Cake-Separated Moxibustion for Mild to Moderate Ulcerative Colitis with Spleen Deficiency and Dampness Accumulation Syndrome. *Chinese Journal of Information on Traditional Chinese Medicine* **24**, 32-35 (2017).

10 Yi, H. *et al.* Therapeutic Efficacy of Yu Kuining Formula Enema Combined with Heat-sensitive Moxibustion in the Treatment of Ulcerative Colitis (Dampness-Heat Accumulation Syndrome) and Its Immunomodulatory Mechanism. *Journal of Emergency in Traditional Chinese Medicine* **30** (2021).

11 Qiang, Z., Yanan, L., Peichao, Q., Yongming, W. & Xinjun, L. "Clinical Study on the 'Using Fire to Chase the Dragon Fire' Method Combined with He-Mu Point Acupuncture for the Treatment of Acute Ulcerative Colitis". *Journal of Emergency in Traditional Chinese Medicine* **27** (2018).

12 Ying, Z., Weijian, Y. & Hongyan, Y. The effect of acupoint catgut embedding on serum IL-2 receptor and T lymphocyte subsets in patients with ulcerative colitis. *Chinese Journal of Integrated Traditional and Western Medicine* **23** (2003).

13 Chunquan, S., Jinling, L., Tiantian, D., Yazheng, P. & Jiguo, Y. Clinical Observation of Umbilical Moxibustion Therapy Combined with Mesalazine in the Treatment of Ulcerative Colitis with Spleen-Kidney Yang Deficiency Syndrome. *Chinese Journal of Integrated Traditional and Western Medicine on Digestion* **25**, 525-528 (2017).

14 Sheng, M. & Guilan, Z. Observation on the Efficacy of Combined Acupuncture and Moxibustion in Treating 60 Cases of Ulcerative Colitis. *Chinese Acupuncture & Moxibustion*, 275-276 (1997).

15 Songmei, W., Xingguo, L., Liqun, Z., Yingchun, X. & Qing, L. Clinical Observation of Shenque (CV8) Herbal-Cake-Separated Moxibustion Therapy for Ulcerative Colitis. *Chinese Acupuncture & Moxibustion* **26** (2006).

16 Hongjun, L., Guoping, L. & Hongyan, L. Clinical Observation of Acupoint Catgut Embedding Therapy for Ulcerative Colitis. *Chinese Acupuncture & Moxibustion*, 261-263 (2006).

17 Jinjie, K., Liming, Y. & Qingxin, C. Observation on the Effect of Banxia Xiexin Decoction Combined with Abdominal Acupuncture in the Treatment of Mild to Moderate Active Ulcerative Colitis with Dampness-Heat Syndrome of the Large Intestine. *Medical Innovation of China* **19**, 90-93 (2022).

18 Ziying, W. Comparison of the efficacy of acupuncture combined with oral Chinese herbal medicine in the treatment of chronic nonspecific ulcerative colitis. *Journal of Zhejiang Chinese Medical University* **39**, 315-316, doi:10.16466/j.issn1005-5509.2015.04.019 (2015).

19 Yanjun, Z., Yujie, C. & Dongdong, Z. The Effect of Regulating Spleen and Stomach with Warming and Cooling Needling Method on Th17/Treg Balance and Cytokine Levels in Patients with Ulcerative Colitis. *Modern Journal of Integrated Traditional Chinese and Western Medicine* **30**, 24-29, doi:10.3969/j.issn.1008-8849.2021.01.005 (2021).

20 Hong, G. The effect of acupoint catgut embedding therapy on the intestinal mucosa of patients with ulcerative colitis. *Modern Journal of Integrated Traditional Chinese and Western Medicine* **29**, 288-290 (2020).

21 Xue, F., Ziqi, L., Xiaoming, G. & Jiena, P. Analysis of the Efficacy of Acupuncture and Moxibustion in Traditional Chinese Medicine for Treating Chronic Ulcerative Colitis. *Clinical Journal of Diabetes World* **17**, 43 (2020).

22 Guiying, Z., Yujie, C., Xuelian, D., Xiaolei, Z. & Yanjun, Z. Clinical Observation on the Treatment of Ulcerative Colitis with Midnight-Midday Ebb-Flow Herbal Retention Enema Combined with Heat-Sensitive Moxibustion. *Journal of Sichuan of Traditional Chinese Medicine* **35**, 77-79 (2017).

23 Liqun, L. Clinical Observation of Bupiwei Xieyinhuo Shengyang Decoction Combined with Heat-sensitive Moxibustion in the Treatment of Ulcerative Colitis. *Journal of Sichuan of Traditional Chinese Medicine* **35**, 95-97 (2017).

24 Baojun, G. *et al.* Observation on the Therapeutic Effect of Acupuncture for Strengthening the Spleen and Nourishing the Kidney in Treating Ulcerative Colitis. *Journal of Sichuan of Traditional Chinese Medicine* **34**, 182-185 (2016).

25 Jie, C. Clinical Observation on Specific Acupoint Thread Embedding Therapy for Chronic Non-Specific Ulcerative Colitis. *Journal of Sichuan of Traditional Chinese Medicine*, 89-90 (2004).

26 Yujie, C., Yanhong, W., Xuelian, D., Xiaolei, Z. & Siyu, D. Randomized Controlled Study on Moxibustion at Shenque and Bazhen Acupoints Combined with Chinese Herbal Medicine Enema in the Treatment of Ulcerative Colitis. *Journal of Sichuan of Traditional Chinese Medicine* **35**, 199-202 (2017).

27 Hailin, W. *et al.* Clinical Study on Tongxie Yaofang Combined with Electroacupuncture in the Treatment of Ulcerative Colitis with Liver-Spleen Disharmony Pattern. *Journal of Sichuan of Traditional Chinese Medicine* **38** (2020).

28 Tianshu, X. & Wei, D. Observation on the Efficacy of Combined Acupuncture and Medication in Treating Mild to Moderate Non-specific Ulcerative Colitis. *Practical Preventive Medicine* **16** (2009).

29 Lingyun, G., Qingshan, Z., Shuyun, Z. & Xinjie, Z. Clinical Observation of Wumei Pill Combined with Thunder-fire Moxibustion in the Treatment of Ulcerative Colitis. *Journal of Clinical Medicine in Practice* **20**, 131-132 (2016).

30 Dongdong, Z., Yanjun, Z. & Yeshan, Z. Observation on the Efficacy of Warm Needling Therapy Combined with Western Medicine in the Treatment of Ulcerative Colitis. *Shanghai Journal of Acupuncture and Moxibustion* **41**, 443-449, doi:10.13460/j.issn.1005-0957.2022.05.0443 (2022).

31 Wensong, X., Lucui, W., Jinzhou, W. & Fang, Z. Efficacy of Acupuncture Combined with Chinese Herbal Medicine in the Treatment of Ulcerative Colitis and Its Impact on Related Indicators. *Shanghai Journal of Acupuncture and Moxibustion* **38**, 378-383, doi:10.13460/j.issn.1005-0957.2019.04.0378 (2019).

32 Qun, S., Yunlu, Z. & Jing, L. Observation on the Efficacy of Indirect Moxibustion with Aconite Cake Combined with Mesalazine in the Treatment of Ulcerative Colitis with Damp-Heat Type. *Shanghai Journal of Acupuncture and Moxibustion* **38**, 374-377, doi:10.13460/j.issn.1005-0957.2019.04.0374 (2019).

33 Qun, S., Jing, L. & Songming, G. Observation on the therapeutic effect of acupuncture at the Shizi point as the main treatment for ulcerative colitis. *Shanghai Journal of Acupuncture and Moxibustion* **31**, 319-320 (2012).

34 Zong, W., Yi, L. & Zhu, Y. Acupoint Catgut Embedding Therapy for Mild to Moderate Ulcerative Colitis: A Report of 34 Cases. *Shaanxi Journal of Traditional Chinese Medicine* **36** (2015).

35 Hongfei, D., Jian, H. & Chunyan, H. Comprehensive therapy for chronic ulcerative colitis: a clinical study of 42 cases. *Shandong Journal of Traditional Chinese Medicine* **27**, 304-306, doi:10.3969/j.issn.0257-358X.2008.05.009 (2008).

36 Weiwei, H., Zhimei, W. & Liangliang, X. Clinical efficacy of indirect moxibustion combined with mesalazine in the treatment of ulcerative colitis and its effects on serum immunoglobulin, interleukin-17, and thromboxane B2. *Journal of Clinical and Experimental Medicine* **17**, 2097-2101 (2018).

37 Jia, Y., Chang, L. & Meiping, Z. Clinical efficacy of Tongxie Decoction combined with acupuncture in the treatment of ulcerative colitis and its effects on serum inflammatory factors and intestinal flora. *Liaoning Journal of Traditional Chinese Medicine* **51**, 159-162, doi:10.13192/j.issn.1000-1719.2024.06.042 (2024).

38 Hu, W. Clinical Study on Electroacupuncture at Dachangshu and Front-Mu Points for Ulcerative Colitis. *Liaoning Journal of Traditional Chinese Medicine* **49**, 173-175, doi:10.13192/j.issn.1000-1719.2022.11.045 (2022).

39 Yanru, D. *et al.* Observation on the Efficacy of Combining Acupuncture and Medication in Treating 89 Cases of Ulcerative Colitis. *Liaoning Journal of Traditional Chinese Medicine* **34**, 65-65, doi:10.3969/j.issn.1000-1719.2007.01.045 (2007).

40 Longling, C., Yonghui, L. & Yuanquan, Z. Clinical Value Analysis of Acupuncture Combined with Changyanqing in the Treatment of Ulcerative Colitis with Dampness-Heat Syndrome of Large Intestine in Active Stage. *Liaoning Journal of Traditional Chinese Medicine* **45**, 811-815, doi:10.13192/j.issn.1000-1719.2018.04.047 (2018).

41 Juhua, Z. Clinical Observation of 34 Cases of Ulcerative Colitis Treated with Ginger-partitioned Moxibustion. *Jiangsu Journal of Traditional Chinese Medicine*, 44-45 (2003).

42 Fei, G. *et al.* Study on the Mechanism of Electroacupuncture Combined with Drug Therapy in the Treatment of UC Based on the Brain-Gut Axis—with 31 Clinical Cases Included. *Jiangsu Journal of Traditional Chinese Medicine*, 65-66,67 (2014).

43 Runlian, Z., Guanglin, N., Xueqing, H. & Shuyu, W. Observation on the Efficacy of Traditional Chinese Medicine Retention Enema Combined with Acupuncture in the Treatment of Ulcerative Colitis. *Jilin Journal of Chinese Medicine* **32** (2012).

44 Yan, C. & Zheng, Y. Study on the Effect of Heat-sensitive Moxibustion Combined with Kangfuxin Liquid Enema in the Treatment of Ulcerative Colitis. *Chinese Nursing Research* **27**, 1970-1971 (2013).

45 Chunxia, X., Rongjun, L., Xiaoli, Y., Jian, W. & Yan, Y. Clinical Observation on Treating Ulcerative Colitis from the Perspective of the Lung Using Jingjie Lianqiao Decoction Combined with Acupuncture at Guiyan Point. *Journal of Hunan University of Chinese Medicine* **43**, 338-342 (2023).

46 Haiping, W. Clinical efficacy of Qingre Huashi Decoction combined with acupuncture in the treatment of ulcerative colitis and its effect on serum inflammatory factors. *Journal of Hubei University of Chinese Medicine* **22**, 78-80 (2020).

47 Yang, T., Weiguo, D. & Shaoqian, T. Combination of Patrinia Mixture and Dense Wave Electroacupuncture in the Treatment of 40 Cases of Ulcerative Colitis. *Henan traditional chinese medicine [henan zhong yi]* **34**, 2362-2365, doi:10.16367/j.issn.1003-5028.2014.12.067 (2014).

48 Hongchang, Z., Shukai, H. & Jinyou, D. 50 Cases of Chronic Non-Specific Ulcerative Colitis Treated with Qi-Replenishing, Blood-Activating, and Collateral-Dredging Formula Combined with Scalp Acupuncture. *Hebei Journal of Traditional Chinese Medicine* **31**, 538-539 (2009).

49 Shiqiang, C. Acupuncture combined with drug enema in the treatment of 40 cases of chronic ulcerative colitis. *Hebei Journal of Traditional Chinese Medicine* 852 (2001).

50 Yanhua, B., Zhenyu, Y. & Yuzhu, S. Clinical Observation of Lian Yang San Retention Enema Combined with Acupuncture Therapy in the Treatment of Chronic Non-Specific Ulcerative Colitis. *Hebei Journal of Traditional Chinese Medicine* **36**, 212+249 (2014).

51 Jun, H., Muxi, L. & Zhenzhen, M. Clinical efficacy of acupuncture combined with umbilical ring moxibustion with ginger for spleen-kidney yang deficiency ulcerative colitis and its effects on related inflammatory factors. *Journal of Guangzhou University of Traditional Chinese Medicine* **32**, 687-689+693, doi:10.13359/j.cnki.gzxbtcm.2015.04.024 (2015).

52 Jianping, H., Li, L., Jianrong, H. & Xiaoquan, D. Colonic Drip Combined with Ginger-partitioned Moxibustion in the Treatment of Ulcerative Colitis: A Clinical Observation. *Journal of Basic Chinese Medicine* **18**, 1027-1029, doi:10.19945/j.cnki.issn.1006-3250.2012.09.042 (2012).

53 Sha, L., Xiaofeng, J., Huan, Q., Yan, S. & Jiaming, L. The Therapeutic Effect of Huangqi Jianzhong Decoction Combined with Moxibustion on Patients with Ulcerative Colitis of Spleen Deficiency and Dampness Accumulation Type and Its Impact on T Cell Subsets. *Chinese Journal of Integrated Traditional and Western Medicine on Digestion* **29**, 879-885 (2021).

54 Guangquan, W. & Minghai, Y. Acupuncture combined with herbal enema in the treatment of 45 cases of ulcerative colitis. *Medical Innovation of China* **7**, 168-169 (2010).

55 Hong, L. Clinical Observation of the Therapeutic Effect of Comprehensive Acupuncture and Moxibustion Therapy on Ulcerative Colitis. *Psychological Doctor* **22**, 6-7 (2016).

56 Yanjun, Z., Yujie, C., Dongdong, Z., Shixu, G. & Qiaoling, W. Observation on the Efficacy of Regulating the Spleen and Stomach with Warming and Cooling Acupuncture Based on the Theory of "Earth as the Pivot and Four Phenomena, One Qi Circulating" in the Treatment of Ulcerative Colitis. *Modern Journal of Integrated Traditional Chinese and Western Medicine* **29**, 3901-3906, doi:10.3969/j.issn.1008-8849.2020.35.006 (2020).

57 Kun, W. & Mei, W. Observation on the Efficacy of Modified Shaoyao Decoction Combined with Acupuncture in Treating Active Ulcerative Colitis with Dampness-Heat Accumulation Syndrome. *Modern Journal of Integrated Traditional Chinese and Western Medicine* **29**, 763-766 (2020).

58 Hong, W., Zhaoxia, C., Xin, W. & Lirong, L. Observation on the Efficacy of Traditional Chinese Medicine in Treating and Nursing Ulcerative Colitis. *Modern Journal of Integrated Traditional Chinese and Western Medicine*, 5205-5206 (2008).

59 Liqun, L., Yan, L. & Lei, Z. Randomized controlled study on the treatment of ulcerative colitis with traditional Chinese medicine retention enema combined with moxibustion. *World Journal of Integrated Traditional and Western Medicine* **12**, 970-973, doi:10.13935/j.cnki.sjzx.170720 (2017).

60 Jiannan, J. The effect of combined acupuncture and medication on T cell subsets in patients with ulcerative colitis. *Shanghai Journal of Acupuncture and Moxibustion* **34**, 63-65, doi:10.13460/j.issn.1005-0957.2015.09.0858 (2015).

61 Yanjun, Z., Yujie, C., Dongdong, Z., Shixu, G. & Qiaoling, W. Clinical Study on Warm Acupuncture Combined with Bukou Buxu Decoction in the Treatment of Ulcerative Colitis with Spleen-Kidney Yang Deficiency Syndrome. *Shaanxi Journal of Traditional Chinese Medicine* **42** (2021).

62 Weiwei, Z., Linkang, P. & Xingshan, L. Observation on the Efficacy of Kuijie Formula Combined with Medicinal Partition Moxibustion in the Treatment of Ulcerative Colitis with Dampness-Heat in the Large Intestine. *Journal of Shandong University of Traditional Chinese Medicine* **44** (2020).

63 Chunling, L., Linghua, L., Xu, H., Jie, Y. & Heng, Z. Clinical Observation of Acupuncture at Mu Points Combined with Oral Mesalazine in the Treatment of Ulcerative Colitis. *Journal of Kunming Medical University* **45**, 72-78 (2024).

64 Hao, X., Jian, G. & Shun, L. Clinical Study on Modified Zhenren Yangzang Decoction Combined with Moxibustion for Ulcerative Colitis with Spleen-Kidney Yang Deficiency Syndrome. *Hebei Journal of Traditional Chinese Medicine* **42** (2020).

65 Li, Z., Lingling, Z. & Xiaojian, J. The efficacy of warm acupuncture combined with Shenling Baizhu Powder in the treatment of ulcerative colitis with spleen deficiency and dampness obstruction syndrome and its effects on brain-gut interaction and inflammatory factors. *Hebei Journal of Traditional Chinese Medicine* **43** (2021).

66 Haiyan, H. Clinical Observation of Acupuncture Combined with Modified Shenling Baizhu Powder in the Treatment of Ulcerative Colitis with Spleen Deficiency and Dampness-Heat Syndrome. *Journal of Guangzhou University of Traditional Chinese Medicine* **39**, 586-593, doi:10.13359/j.cnki.gzxbtcm.2022.03.021 (2022).

67 Bing, W. Stellate ganglion block combined with acupoint catgut embedding for ulcerative colitis. *Anhui Medical and Pharmaceutical Journal* **17**, 1784-1785 (2013).

68 Shukai, H., Hongchang, Z., Wenge, S. & Jinyou, D. Treatment of 50 Cases of Chronic Ulcerative Colitis with the Method of Tonifying Qi, Activating Blood Circulation, and Dredging Collaterals Combined with Scalp Acupuncture Therapy. *Journal of Traditional Chinese Medicine* **50**, 330-331, doi:10.13288/j.11-2166/r.2009.04.038 (2009).

69 Yanru, D., Yanxia, Z., Shengzhen, S., Wan, Z. & Zhikun, W. Clinical Efficacy Observation of Combining Acupuncture and Medicine in Treating 89 Cases of Ulcerative Colitis. *Journal of Traditional Chinese Medicine* **49**, 314-316, doi:10.3321/j.issn:1001-1668.2008.04.010 (2008).

70 Hongchang, Z., Shukai, H. & Jinyou, D. Observation on the Efficacy of Scalp Acupuncture Combined with Sulfasalazine in the Treatment of 46 Cases of Chronic Ulcerative Colitis. *Journal of Emergency in Traditional Chinese Medicine* **19** (2010).

71 Yanru, D., Diangui, L., Wan, Z., Feng, Z. & Qiquan, L. The effect of combining acupoint therapy with medication on clinical efficacy and SOD, NO levels in patients with ulcerative colitis (UC). *China Journal of Chinese Materia Medica* **33**, 2404-2406, doi:10.3321/j.issn:1001-5302.2008.20.029 (2008).

72 Xiaoying, L. *et al.* Clinical efficacy of moxibustion in the treatment of ulcerative colitis and its effect on vitamin D receptors. *Journal of Acupuncture and Tuina Science* **21**, 40-50 (2023).

73 Xiangdong, W., Xuanfeng, D. & Weili, D. 40 Cases of Ulcerative Colitis Treated with Changning Enema Combined with Du Moxibustion. *Shaanxi Journal of Traditional Chinese Medicine* **33**, 1127-1128 (2012).

74 Dongjiang, D. *et al.* Clinical Observation on Acupoint Catgut Embedding Combined with Modified Pulsatilla Decoction Enema in the Treatment of Chronic Non-Specific Ulcerative Colitis. *Hebei Journal of Traditional Chinese Medicine* **34**, 721-722 (2012).

75 Jian, H. 36 cases of chronic nonspecific ulcerative colitis treated with herbal enema combined with acupuncture. *Journal of Guizhou University of Traditional Chinese Medicine*, 12, doi:10.16588/j.cnki.issn1002-1108.2001.04.009 (2001).

76 Xiaolan, L., Xingqian, L., Kai, C., Qingfeng, M. & Qing, Y. Clinical Study on the Combination of Chinese and Western Medicine Retention Enema with Acupuncture in the Treatment of Ulcerative Colitis. *Journal of Colorectal & Anal Surgery*, 161-163 (2008).

Table S3.2: Descriptions of the included acupuncture and related therapies.

Study ID	Acupuncture-related therapy	Acupoints	Treatment Schedule
Zhao 2012	ACE+RE	Tianshu(ST25) Xiawan(CV10) Shangjuxu(ST37) Guanyuan(CV4) Quchi(LI11) Neiting(ST44)	Once per week
Zhang 2003	ACU	Sanjiaoshu(BL22) Dachangshu(BL25)	30 minutes per session, once daily; 10 days per treatment course
Wang 2022a	ACU+CHM+WM	Shangjuxu(ST37) Yinlingquan(SP9) Tianshu(ST25) Zhigou(TE6) Neiting(ST44) Dachangshu(BL25) Shuidao(ST28) Wailing(ST26)	30 minutes per session, once daily; 5 days per course with 2-day interval before the next
Cheng 2009	MOX+CHM+WM	Zusanli(ST36) Shangjuxu(ST37) Xiajuxu(ST39) Fenglong(ST40) Shousanli(LI10) Hegu(LI4)	10 seconds per session
Fan 2021	MOX+CHM	Shenque(CV8)	40–45 minutes per session, once daily; 7 days per treatment course
Zhou 2008	EA+MOX+WM	Tianshu(ST25) Zhongwan(CV12) Qihai(CV6) Zusanli(ST36) Shangjuxu(ST37) Yinlingquan(SP9)	30 minutes per session, once daily; 10 days per course with 2-day interval
Xu 2022	MOX+CHM	Tianshu(ST25) Zhongwan(CV12) Pishu(BL20) Huiyang(BL35)	30 minutes per session, once per week
Zhao 2019	MOX	Shenque(CV8)	1.5 hours per session, twice weekly
Yang 2017	MOX	Pishu(BL20) Zhongwan(CV12) Zusanli(ST36) Dachangshu(BL25) Tianshu(ST25) Shangjuxu(ST37)	15 minutes per session, once daily
He 2021	MOX+RE	Dachangshu(BL25) Tianshu(ST25) Shangjuxu(ST37)	Once daily; 10 days per course with 2-day interval before the next
Zhao 2018	ACU+CHM	Tianshu(ST25) Shangjuxu(ST37)	30 minutes per session, once daily; 6 days treatment, 1 day rest
Zhu 2003	ACE	Zhongwan(CV12) Zusanli(ST36) Tianshu(ST25)	Once every two weeks
Sun 2017	MOX	Shenque(CV8)	2 hours per session

Ma 1997	ACU	Tianshu(ST25) Zhongwan(CV12) Guanyuan(CV4) Zusanli(ST36) Taichong(LR3) Dachangshu(BL25) Shenshu(BL23) Shangliao(BL31) Ciliao(BL32)	30 minutes per session, once daily
Wang 2006	MOX	Shenque(CV8)	Once daily; 10-day course with 3-day interval before the next
Li 2006	ACE	Dachangshu(BL25) Zusanli(ST36) Shangjuxu(ST37)	Not reported
Kang 2022	ACU+CHM	Zhongwan(CV12) Xiawan(CV10) Qihai(CV6) Guanyuan(CV4) Daheng(SP15) Wailing(ST26) Huaroumen(ST24)	30 minutes per session, three times per week
Wu 2015	ACU+MOX+CHM	Zhongwan(CV12) Xiawan(CV10) Shenque(CV8) Tianshu(ST25) Guilai(ST27) Qihai(CV6) Guanyuan(CV4) Zusanli(ST36) Yinlingquan(SP9)	ACU: 30 min/session, once daily, 10-day course; MOX: every other day
Zhang 2021a	WA+WM	Pishu(BL20) Zhongwan(CV12) Zusanli(ST36) Tianshu(ST25) Taibai(SP3) Shangjuxu(ST37) Sanyinjiao(SP6) Guanyuan(CV4)	20 minutes per session, five times per week; 10 days per course
Gong 2020	ACE	Zusanli(ST36) Guanyuan(CV4) Pishu(BL20)	Not reported
Feng 2020	ACU	Guanyuan(CV4) Qihai(CV6) Changqiang(GV1) Dachangshu(BL25) Tianshu(ST25)	20 minutes per session, once daily
Zu 2017	MOX+RE+WM	Dachangshu(BL25) Pishu(BL20) Shangjuxu(ST37) Xiajuxu(ST39) Tianshu(ST25)	Saturating desensitization moxibustion dose, once daily; 14-day course, 2–3 day interval
Lv 2017a	MOX+CHM+WM	Dachangshu(BL25) Pishu(BL20) Shangjuxu(ST37) Xiajuxu(ST39) Tianshu(ST25)	Saturating moxibustion dose, once daily; 14-day course
Guo 2016	ACU+MOX+WM	Shenshu(BL23) Pishu(BL20) Dachangshu(BL25) Weishu(BL21) Gongsun(SP4) Tianshu(ST25) Zusanli(ST36) Taixi(KI3) Zhongwan(CV12) Guanyuan(CV4)	ACU: 30 min/session, once daily; MOX: 5 moxa cones per acupoint, once daily; 10-day course with 2-day interval
Chen 2004	ACE	Dachangshu(BL25) Tianshu(ST25) Zusanli(ST36)	Once per month
Chang 2017	MOX+RE+WM	Shenque(CV8)	40 minutes per session, once daily
Wang 2020a	EA+CHM+WM	Zhongwan(CV12) Tianshu(ST25) Zusanli(ST36) Yinlingquan(SP9)	30 minutes per session, once daily; 4-week treatment course

		Pishu(BL20) Taichong(LR3)	
Xu 2009	ACU+CHM	Shangjuxu(ST37) Xiajuxu(ST39) Yinlingquan(SP9) Gongsun(SP4) Tianshu(ST25) Hegu(LI4) Quchi(LI11)	30–40 minutes per session, once daily
Gu 2016	MOX+CHM	Shenque(CV8) Mingmen(GV4) Guanyuan(CV4) Zhongwan(CV12) Zusanli(ST36) Tianshu(ST25) Dachangshu(BL25)	Eight moxa cones per point, twice daily
Zhang 2022	EA+WM	Pishu(BL20) Zhongwan(CV12) Zusanli(ST36) Tianshu(ST25) Taibai(SP3) Shangjuxu(ST37) Sanyinjiao(SP6) Guanyuan(CV4)	20 minutes per session, once daily; 5-day course with 2-day interval
Xie 2019	ACU+CHM	Pishu(BL20) Guanyuan(CV4) Weishu(BL21) Tianshu(ST25) Zhongwan(CV12) Zusanli(ST36) Qihai(CV6) Dachangshu(BL25) Gongsun(SP4)	20–30 minutes per session, once daily; 1-week treatment course
Shen 2019	MOX+WM	Zhongwan(CV12) Tianshu(ST25) Guanyuan(CV4) Shangjuxu(ST37)	Two moxa cones per point, once daily; 12-day course with 3-day interval
Shen 2012	ACU	Zhongwan(CV12) Tianshu(ST25) Guanyuan(CV4) Zusanli(ST36) Shenque(CV8)	30 minutes per session, once daily; 10-day course with 7-day interval
Zong 2015	ACE	Zhongwan(CV12) Zusanli(ST36) Tianshu(ST25)	Once every two weeks
Du 2008	ACU+RE+CHM	Zusanli(ST36) Shangjuxu(ST37) Xiajuxu(ST39)	30 minutes per session, once daily
Hou 2018	MOX+WM	Qihai(CV6) Tianshu(ST25) Zhongwan(CV12) Yinlingquan(SP9) Shangjuxu(ST37)	Burn moxa fully per session, once daily; 15-day course with 3-day interval
Ye 2024	ACU+CHM	Tianshu(ST25) Quchi(LI11) Shangjuxu(ST37)	Two to three times per week
Wang 2022b	ACU	Dachangshu(BL25) Tianshu(ST25)	30 minutes per session, once daily
Du 2007	AA+CHM	Spleen(MA-IC 3) Large Intestine(MA-IC 5) Endocrine(MA-AT 1) Sympathetic(MA-AH 7) Subcortex(MA-AT 4)	Press 3–5 times daily; 10–20 presses per acupoint
Cong 2018	ACU+CHM	Dachangshu(BL25) Shenque(CV8) Tianshu(ST25) Shangjuxu(ST37) Sanyinjiao(SP6) Pishu(BL20) Hegu(LI4) Xiajuxu(ST39) Zusanli(ST36) Guanyuan(CV4) Quchi(LI11)	15 minutes per session, once daily; 5-day course with 2-day interval

		Neiting(ST44)	
Zhou 2003	MOX	Zhongwan(CV12) Tianshu(ST25) Zusanli(ST36)	5–7 moxa cones until skin reddens, once daily; 10-day course
Ge 2014	EA+WM	Ganshu(BL18) Pishu(BL20) Dachangshu(BL25) Shenshu(BL23) Ciliao(BL32) Tianshu(ST25) Qihai(CV6) Guanyuan(CV4) Shangjuxu(ST37) Sanyinjiao(SP6) Taichong(LR3)	30 minutes per session, once daily; 5-day course with 2–3 day interval
Zhang 2012	ACU+RE	Pishu(BL20) Zhongwan(CV12) Tianshu(ST25) Zusanli(ST36) Shangjuxu(ST37)	30 minutes per session, once daily; 2-week treatment course
Chen 2013	MOX+RE+WM	Tianshu(ST25) Shenque(CV8) Guanyuan(CV4) Zhongwan(CV12) Zusanli(ST36) Qihai(CV6) Pishu(BL20) Shangjuxu(ST37) Yinlingquan(SP9)	Not reported
Xu 2023	ACU+CHM+RE	Yinbai(SP1) Shaoshang(LU11)	20 minutes per session, every other day
Wang 2020b	ACU+CHM+WM	Dachangshu(BL25) Shangjuxu(ST37) Xiajuxu(ST39) Shenque(CV8) Tianshu(ST25) Zusanli(ST36)	15 minutes per session, once daily; 5-day course with 2-day interval
Teng 2014	EA+RE+WM	Zhongwan(CV12) Tianshu(ST25) Guanyuan(CV4) Zusanli(ST36) Sanyinjiao(SP6)	Not reported
Zhang 2009	ACU+CHM	Scalp Acupuncture Stomach Zone<br>Scalp Acupuncture Intestinal Zone	5–10 minutes per session, once daily
Cao 2001	ACU+RE	Pishu(BL20) Zhangmen(LR13) Tianshu(ST25) Zusanli(ST36) Zhongwan(CV12) Guanyuan(CV4) Mingmen(GV4) Gongsun(SP4)	20 minutes per session, once daily
Bao 2014	ACU+RE	Guanyuan(CV4) Tianshu(ST25) Zusanli(ST36) Yinlingquan(SP9) Sanyinjiao(SP6)	20 minutes per session, once daily
He 2015	ACU+WM	Tianshu(ST25) Qihai(CV6) Guanyuan(CV4)<br>Hegu(LI4) Shangjuxu(ST37) Sanyinjiao(SP6)	Once daily; 5-day course with 2-day interval
Hui 2012	MOX+RE	Shenque(CV8) Zhongwan(CV12) Zusanli(ST36)	Three moxa cones per acupoint, once daily
Lu 2021	MOX+CHM	Shenque(CV8)	20 minutes per session, once every 2 days

Wang 2010	ACU+RE	Tianshu(ST25) Daheng(SP15) Zusanli(ST36) Shenque(CV8)	30 minutes per session, once daily; 7-day course with 1–2 day interval
Li 2016	WA	Tianshu(ST25) Qihai(CV6) Guanyuan(CV4) Changqiang(GV1) Dachangshu(BL25) Zusanli(ST36) Sanyinjiao(SP6)	20 minutes per session, once every 2 days
Zhang 2020	WA	Pishu(BL20) Zhongwan(CV12) Zusanli(ST36) Tianshu(ST25) Taibai(SP3) Shangjuxu(ST37) Sanyinjiao(SP6) Guanyuan(CV4)	20 minutes per session, 5 times per week; 10 sessions per course
Wang 2020c	ACU+CHM+WM	Quchi(LI11) Hegu(LI4) Tianshu(ST25) Neiting(ST44) Shangjuxu(ST37)	15–20 minutes per session, once daily; 5-day course with 2-day interval
Wang 2008	MOX+CHM	Shenque(CV8) Zusanli(ST36) Tianshu(ST25) Shangjuxu(ST37)	20 minutes per session, once daily; 10-day course with 2-day interval
Lv 2017b	MOX+RE+WM	Dachangshu(BL25) Pishu(BL20) Shangjuxu(ST37) Xiajuxu(ST39) Tianshu(ST25)	15 minutes per session, once daily
Jia 2015	MOX+WM	Zhongji(CV3) Guanyuan(CV4) Qihai(CV6) Tianshu(ST25) Daheng(SP15) Dachangshu(BL25) Zusanli(ST36) Shangjuxu(ST37) Sanyinjiao(SP6) Taichong(LR3)	Once daily
Zhang 2021b	WA+CHM+WM	Zhongwan(CV12) Guanyuan(CV4) Qihai(CV6) Zusanli(ST36) Tianshu(ST25) Yinlingquan(SP9)	Once daily; 6-day course with 1-day interval
Zheng 2020	MOX+RE	Shenque(CV8)	30 minutes per session, once daily; 7-day course
Li 2024	ACU+WM	Tianshu(ST25)	20 minutes per session, once daily; 5-day course with 2-day interval
Xiu 2020	MOX+CHM+WM	Zhongwan(CV12) Guanyuan(CV4) Qihai(CV6) Tianshu(ST25)	30–40 minutes per session, once daily
Zhou 2021	WA+CHM+WM	Guanyuan(CV4) Zhongwan(CV12) Tianshu(ST25) Zusanli(ST36) Shangjuxu(ST37) Pishu(BL20) Shenshu(BL23) Dachangshu(BL25)	30 minutes per session, once daily; 5 days per course with 2-day interval before the next
Hua 2022	ACU+CHM+WM	Quchi(LI11) Tianshu(ST25) Guanyuan(CV4) Shangjuxu(ST37) Zusanli(ST36)	30 minutes per session, once daily; 5 days per course with 2-day interval before the next

Wang 2013	ACE	Pishu(BL20) Weishu(BL21) Dachangshu(BL25)	Once every 15 days
Han 2009	ACU	Scalp Acupuncture Stomach Zone<br>Scalp Acupuncture Intestinal Zone	5–10 minutes per session, once daily
Du 2008a	AA+CHM	Spleen(MA-IC 3) Large Intestine(MA-IC 5) Endocrine(MA-AT 1)<br>Sympathetic(MA-AH 7) Subcortex(MA-AT 4)	Press 3–5 times daily; 10–20 presses per acupoint
Zhang 2010	ACU+WM	Scalp Acupuncture Stomach Zone<br>Scalp Acupuncture Intestinal Zone	5–10 minutes per session, once daily; 20-day course with 2-day interval
Du 2008b	AA+CHM	Spleen(MA-IC 3) Large Intestine(MA-IC 5) Endocrine(MA-AT 1)<br>Sympathetic(MA-AH 7) Subcortex(MA-AT 4)	Press 3–5 times daily; 10–20 presses per acupoint
Lin 2023	MOX	Qihai(CV6) Tianshu(ST25) Shangjuxu(ST37) Xiajuxu(ST39)	Three times per week
Wang 2012	MOX+RE	Shenque(CV8)	6–8 hours
Duan 2012	ACE+RE	Tianshu(ST25) Dachangshu(BL25) Guanyuan(CV4) Zhongwan(CV12)<br>Zusanli(ST36) Yinlingquan(SP9)	Once every 2 weeks; 14-day course with 7-day interval
He 2001	ACU+RE	Zusanli(ST36) Shangjuxu(ST37) Xiajuxu(ST39)	30 minutes per session, once daily; 14-day course with 3-day interval
Li 2008	ACU+MOX+RE	Zhongwan(CV12) Qihai(CV6)<br>Zusanli(ST36) Sanyinjiao(SP6) Hegu(LI4) Dachangshu(BL25)<br>Tianshu(ST25) Shangjuxu(ST37)	30 minutes per session

ACU,acupuncture;MOX,moxibustion;WA,warm acupuncture;ACE,acupoint catgut embedding;EA,electroacupuncture;AA,auricular acupressure;CHM,Chinese Herbal Medicine;RE,retention enema;WM,western medicine.

Table S3.3: Frequency table of acupoints.

Acupoints	Frequency/Count	Percentage/%	Acupoints	Frequency/Count	Percentage/%
Tianshu(ST25)	52	14.9%	Xiawan(CV10)	3	0.9%
Zusanli(ST36)	38	10.9%	Changqiang(GV1)	2	0.6%
Shangjuxu(ST37)	33	9.5%	Ciliao(BL32)	2	0.6%
Zhongwan(CV12)	29	8.3%	Mingmen(GV4)	2	0.6%
Guanyuan(CV4)	28	8.0%	Wailing(ST26)	2	0.6%
Dachangshu(BL25)	24	6.9%	Fenglong(ST40)	1	0.3%
Pishu(BL20)	19	5.4%	Ganshu(BL18)	1	0.3%
Shenque(CV8)	17	4.9%	Guilai(ST27)	1	0.3%
Qihai(CV6)	15	4.3%	Huaroumen(ST24)	1	0.3%
Sanyinjiao(SP6)	11	3.2%	Huiyang(BL35)	1	0.3%
Xiajuxu(ST39)	10	2.9%	Sanjiaoshu(BL22)	1	0.3%
Yinlingquan(SP9)	10	2.9%	Shangliao(BL31)	1	0.3%
Hegu(LI4)	6	1.7%	Shaoshang(LU11)	1	0.3%
Quchi(LI11)	6	1.7%	Shousanli(LI10)	1	0.3%
Gongsun(SP4)	4	1.1%	Shuidao(ST28)	1	0.3%
Neiting(ST44)	4	1.1%	Taixi(KI3)	1	0.3%
Shenshu(BL23)	4	1.1%	Yinbai(SP1)	1	0.3%
Taichong(LR3)	4	1.1%	Zhangmen(LR13)	1	0.3%
Daheng(SP15)	3	0.9%	Zhigou(TE6)	1	0.3%
Taibai(SP3)	3	0.9%	Zhongji(CV3)	1	0.3%
Weishu(BL21)	3	0.9%			

Table S3.4: Definition, Anatomical Localization, and Clinical Relevance of Acupoints

Acupoint Name (Code)	Anatomical Localization	Meridian Affiliation	Clinical Relevance & Rationale for UC Treatment
Tianshu (ST25)	2 cun lateral to the center of the umbilicus.	Stomach Meridian	Front-Mu point of the Large Intestine. The primary acupoint for regulating intestinal function and Qi movement in the abdomen. Directly addresses core UC symptoms like diarrhea, abdominal pain, and constipation.
Zusanli (ST36)	3 cun below ST35, one finger-breadth lateral to the anterior border of the tibia.	Stomach Meridian	He-Sea point of the Stomach. A fundamental point for strengthening the body's constitution (Tonifying Qi and Blood), regulating gastrointestinal function, and modulating systemic immunity.
Shangjuxu (ST37)	3 cun below ST36, one finger-breadth lateral to the anterior border of the tibia.	Stomach Meridian	Lower He-Sea point of the Large Intestine. A key distal point for treating disorders of the large intestine, specifically indicated for diarrhea and dysentery.
Zhongwan (CV12)	On the anterior midline, 4 cun above the umbilicus.	Conception Vessel	Front-Mu point of the Stomach; Influential point of the Fu-organs. Regulates the function of the Stomach and Spleen, alleviates epigastric and abdominal distension, and addresses digestive weakness.
Guanyuan (CV4)	On the anterior midline, 3 cun below the umbilicus.	Conception Vessel	A major point for strengthening the body's foundational energy (Tonifying Yuan Qi). Used to treat chronic conditions and strengthen the lower body.

Dachangshu (BL25)	At the level of the inferior border of the spinous process of the 4th lumbar vertebra (L4), 1.5 cun lateral to the posterior midline.	Bladder Meridian	Back-Shu point of the Large Intestine. The key point on the back corresponding to the large intestine. Regulates intestinal function and treats lower back pain.
Pishu (BL20)	At the level of the inferior border of the spinous process of the 11th thoracic vertebra (T11), 1.5 cun lateral to the posterior midline.	Bladder Meridian	Back-Shu point of the Spleen. Strengthens the Spleen's function of transformation and transportation, which is central to resolving dampness and diarrhea in UC.
Shenque (CV8)	In the center of the umbilicus.	Conception Vessel	The umbilicus itself. A pivotal point for treating abdominal disorders. Often stimulated by indirect moxibustion to warm Yang and dispel internal cold-dampness.
Qihai (CV6)	On the anterior midline, 1.5 cun below the umbilicus.	Conception Vessel	Sea of Qi. Benefits and regulates the Qi of the entire body. Used for Qi deficiency and to alleviate abdominal pain and distension.
Sanyinjiao (SP6)	3 cun directly above the tip of the medial malleolus, on the posterior border of the tibia.	Spleen Meridian	Intersection point of the Spleen, Liver, and Kidney meridians. Regulates the lower abdomen, strengthens the Spleen, and resolves dampness. A key point for gynecological and digestive issues.
Xiajuxu (ST39)	3 cun below ST37, one finger-breadth lateral to the anterior border of the tibia.	Stomach Meridian	Lower He-Sea point of the Small Intestine. Often used in conjunction with Shangjuxu (ST37) for intestinal disorders, especially those involving malabsorption or undigested food in the stool.

Yinlingquan (SP9)	On the lower border of the medial condyle of the tibia, in the depression between the posterior border of the tibia and the gastrocnemius muscle.	Spleen Meridian	He-Sea point of the Spleen. The primary point for resolving dampness in the body, a key pathological factor in UC characterized by diarrhea with mucus.
Hegu (LI4)	On the dorsum of the hand, between the 1st and 2nd metacarpal bones, in the middle of the 2nd metacarpal bone on the radial side.	Large Intestine Meridian	Yuan-Source point of the Large Intestine. A powerful point for regulating the intestines and relieving pain. Also used for its surface-reaching effect to expel external pathogens.
Quchi (LI11)	When the elbow is flexed, the point is in the depression at the lateral end of the transverse cubital crease, midway between LU5 and the lateral epicondyle of the humerus.	Large Intestine Meridian	He-Sea point of the Large Intestine. Clears heat and damp-heat from the body, reduces inflammation, and regulates Qi and Blood.
Gongsun (SP4)	In the depression distal and inferior to the base of the 1st metatarsal bone, at the border of the red and white skin.	Spleen Meridian	Luo-Connecting point of the Spleen; Confluent point linking with the Chong Mai. Regulates the Stomach and Spleen, alleviates epigastric and abdominal pain, and calms the spirit.
Neiting (ST44)	Proximal to the web margin between the 2nd and 3rd toes, in the depression distal to the 2nd metatarsophalangeal joint.	Stomach Meridian	Ying-Spring point of the Stomach. Clears stomach heat and calms acute episodes of epigastric pain and discomfort.
Shenshu (BL23)	At the level of the inferior border of the spinous process of the 2nd lumbar vertebra (L2), 1.5 cun lateral to the posterior midline.	Bladder Meridian	Back-Shu point of the Kidney. Tonifies Kidney Qi and Yang, which is essential for treating chronic conditions and UC with cold manifestations.

Taichong (LR3)	On the dorsum of the foot, in the depression distal to the junction of the 1st and 2nd metatarsal bones.	Liver Meridian	Yuan-Source and Shu-Stream point of the Liver. Soothes the Liver Qi, alleviates emotional stress and its impact on digestion (Liver-Spleen disharmony), and relieves pain.
Daheng (SP15)	4 cun lateral to the center of the umbilicus.	Spleen Meridian	Regulates the intestines and Spleen, and is effective in relieving abdominal pain and distension.
Taibai (SP3)	On the medial side of the foot, in the depression proximal and inferior to the head of the 1st metatarsal bone, at the border of the red and white skin.	Spleen Meridian	Yuan-Source and Shu-Stream point of the Spleen. Strengthens the Spleen's function and resolves dampness from its root.
Weishu (BL21)	At the level of the inferior border of the spinous process of the 12th thoracic vertebra (T12), 1.5 cun lateral to the posterior midline.	Bladder Meridian	Back-Shu point of the Stomach. Regulates the Stomach and harmonizes the Middle Jiao, addressing nausea and epigastric discomfort.
Xiawan (CV10)	On the anterior midline, 2 cun above the umbilicus.	Conception Vessel	Located between the Stomach and Intestines, it helps separate the clear from the turbid and regulates the lower part of the Stomach.
Changqiang (GV1)	Below the tip of the coccyx, in the depression between the tip of the coccyx and the anus.	Governor Vessel	A local point for treating anorectal disorders, hemorrhoids, and diarrhea.
Ciliao (BL32)	In the 2nd posterior sacral foramen.	Bladder Meridian	A local point for regulating the lower jiao, treating urogenital and intestinal disorders.
Mingmen (GV4)	On the posterior midline, in the depression below the spinous process of the 2nd lumbar vertebra (L2).	Governor Vessel	Gate of Life. Warms and strengthens Kidney Yang, providing the foundational warmth for all bodily functions, including digestion.

Wailing (ST26)	1 cun below ST25, 2 cun lateral to the anterior midline.	Stomach Meridian	A local point in the lower abdomen, used to regulate Qi and relieve pain and distension.
Fenglong (ST40)	8 cun superior to the tip of the external malleolus, one finger-breadth lateral to ST38.	Stomach Meridian	Luo-Connecting point of the Stomach. Resolves phlegm and dampness systemically, and calms the spirit.
Ganshu (BL18)	At the level of the inferior border of the spinous process of the 9th thoracic vertebra (T9), 1.5 cun lateral to the posterior midline.	Bladder Meridian	Back-Shu point of the Liver. Regulates Liver Qi and is used when emotional stress is a major contributing factor to UC.
Guilai (ST29)	4 cun below the umbilicus, 2 cun lateral to the anterior midline.	Stomach Meridian	A local point in the lower abdomen, regulates Qi and blood in the uterus and intestines.
Huaroumen (ST24)	1 cun above ST25, 2 cun lateral to the anterior midline.	Stomach Meridian	A local point in the upper abdomen, regulates Stomach and Spleen Qi.
Huiyang (BL35)	0.5 cun lateral to the tip of the coccyx.	Bladder Meridian	A local point for anorectal disorders and regulating the lower jiao.
Sanjiaoshu (BL22)	At the level of the inferior border of the spinous process of the 1st lumbar vertebra (L1), 1.5 cun lateral to the posterior midline.	Bladder Meridian	Back-Shu point of the Sanjiao (Triple Energizer). Regulates the water passages and Qi movement throughout the body.
Shangliao (BL31)	In the 1st posterior sacral foramen.	Bladder Meridian	A local point for regulating the lower jiao and treating urogenital and intestinal disorders.
Shaoshang (LU11)	On the radial side of the thumb, about 0.1 cun posterior to the corner of the nail.	Lung Meridian	Jing-Well point of the Lung. Clears heat and restores consciousness, used in acute or febrile conditions.

Shousanli (LI10)	On the radial side of the dorsal aspect of the forearm, on the line connecting LI5 with LI11, 2 cun below LI11.	Large Intestine Meridian	Regulates intestinal function and strengthens the body's resistance.
Shuidao (ST28)	3 cun below the umbilicus, 2 cun lateral to the anterior midline.	Stomach Meridian	A local point that regulates the water passages, addressing edema and urinary dysfunction.
Taixi (KI3)	In the depression between the medial malleolus and the Achilles tendon, level with the tip of the medial malleolus.	Kidney Meridian	Yuan-Source and Shu-Stream point of the Kidney. Tonifies Kidney Yin and Yang, the foundation of all bodily Yin and Yang.
Yinbai (SP1)	On the medial side of the great toe, about 0.1 cun posterior to the corner of the nail.	Spleen Meridian	Jing-Well point of the Spleen. Regulates the Spleen, stops bleeding, and calms the spirit.
Zhangmen (LR13)	On the lateral side of the abdomen, below the free end of the 11th floating rib.	Liver Meridian	Front-Mu point of the Spleen; Influential point of the Zang-organs. Regulates the Spleen and Liver, harmonizing the Middle Jiao.
Zhigou (TE6)	On the dorsal aspect of the forearm, on the line connecting TE4 with TE5, 3 cun proximal to the dorsal wrist crease, between the radius and ulna.	Triple Energizer Meridian	Jing-River point of the Sanjiao. Regulates Qi movement, clears heat, and is specific for treating hypochondriac pain and constipation.
Zhongji (CV3)	On the anterior midline, 4 cun below the umbilicus.	Conception Vessel	Front-Mu point of the Bladder. Regulates the lower jiao, addressing urogenital disorders and lower abdominal pain.

Appendix 4: Risk of bias of randomized clinical trials

Study ID	Randomization process	Deviations from intended interventions	Mising outcome data	Measurement of the outcome	Selection of the reported result	Over all
Zhao 2012	Low	Some concerns	Low	Low	Low	Some concerns
Zhang 2003	Some concerns	Some concerns	Low	Low	Low	High
Wang 2022a	Low	Some concerns	Low	Low	Low	Some concerns
Cheng 2009	Low	Some concerns	Low	Low	Low	Some concerns
Fan 2021	Low	Some concerns	Low	Low	Low	Some concerns
Zhou 2008	Low	Some concerns	Low	Low	Low	Some concerns
Xu 2022	Low	Some concerns	Low	Low	Low	Some concerns
Zhao 2019	Low	Some concerns	Low	Low	Low	Some concerns
Yang 2017	Low	Some concerns	Low	Low	Low	Some concerns
He 2021	Low	Some concerns	Low	Low	Low	Some concerns
Zhao 2018	Low	Some concerns	Low	Low	Low	Some concerns
Zhu 2003	Low	Some concerns	Low	Low	Low	Some concerns
Sun 2017	Some concerns	Some concerns	Low	Low	Low	High
Ma 1997	Some concerns	Some concerns	Low	Low	Low	High
Wang 2006	Low	Some concerns	Low	Low	Low	Some concerns
Li 2006	Low	Some concerns	Low	Low	Low	Some concerns

Kang 2022	Low	Some concerns	Low	Low	Low	Some concerns
Wu 2015	Some concerns	Some concerns	Low	Low	Low	High
Zhang 2021a	Low	Some concerns	Low	Low	Low	Some concerns
Gong 2020	Low	Some concerns	Low	Low	Low	Some concerns
Feng 2020	Some concerns	Some concerns	Low	Low	Low	High
Zu 2017	Low	Some concerns	Low	Low	Low	Some concerns
Lv 2017a	Low	Some concerns	Low	Low	Low	Some concerns
Guo 2016	Some concerns	Some concerns	Low	Low	Low	High
Chen 2004	Some concerns	Some concerns	Low	Low	Low	High
Chang 2017	Low	Some concerns	Low	Low	Low	Some concerns
Wang 2020a	Low	Some concerns	Low	Low	Low	Some concerns
Xu 2009	Some concerns	Some concerns	Low	Low	Low	High
Gu 2016	Some concerns	Some concerns	Low	Low	Low	High
Zhang 2022	Low	Some concerns	Low	Low	Low	Some concerns
Xie 2019	Low	Some concerns	Low	Low	Low	Some concerns
Shen 2019	Low	Some concerns	Low	Low	Low	Some concerns
Shen 2012	Some concerns	Some concerns	Low	Low	Low	High
Zong 2015	Some concerns	Some concerns	Low	Low	Low	High
Du 2008	Some concerns	Some concerns	Low	Low	Low	High

Hou 2018	Low	Some concerns	Low	Low	Low	Some concerns
Ye 2024	Low	Some concerns	Low	Low	Low	Some concerns
Wang 2022b	Some concerns	Some concerns	Low	Low	Low	High
Du 2007	Low	Some concerns	Low	Low	Low	Some concerns
Cong 2018	Low	Some concerns	Low	Low	Low	Some concerns
Zhou 2003	Some concerns	Some concerns	Low	Low	Low	High
Ge 2014	Some concerns	Some concerns	Low	Low	Low	High
Zhang 2012	Low	Some concerns	Low	Low	Low	Some concerns
Chen 2013	Some concerns	Some concerns	Low	Low	Low	High
Xu 2023	Low	Some concerns	Low	Low	Low	Some concerns
Wang 2020b	Some concerns	Some concerns	Low	Low	Low	High
Teng 2014	Some concerns	Some concerns	Low	Low	Low	High
Zhang 2009	Some concerns	Some concerns	Low	Low	Low	High
Cao 2001	Some concerns	Some concerns	Low	Low	Low	High
Bao 2014	Some concerns	Some concerns	Low	Low	Low	High
He 2015	Low	Some concerns	Low	Low	Low	Some concerns
Hui 2012	Low	Some concerns	Low	Low	Low	Some concerns
Lu 2021	Low	Some concerns	Low	Low	Low	Some concerns
Wang 2010	Some concerns	Some concerns	Low	Low	Low	High

Li 2016	Low	Some concerns	Low	Low	Low	Some concerns
Zhang 2020	Low	Some concerns	Low	Low	Low	Some concerns
Wang 2020c	Some concerns	Some concerns	Low	Low	Low	High
Wang 2008	Some concerns	Some concerns	Low	Low	Low	High
Lv 2017b	Low	Some concerns	Low	Low	Low	Some concerns
Jia 2015	Low	Some concerns	Low	Low	Low	Some concerns
Zhang 2021b	Low	Some concerns	Low	Low	Low	Some concerns
Zheng 2020	Low	Some concerns	Low	Low	Low	Some concerns
Li 2024	Low	Some concerns	Low	Low	Low	Some concerns
Xiu 2020	Low	Some concerns	Low	Low	Low	Some concerns
Zhou 2021	Low	Some concerns	Low	Low	Low	Some concerns
Hua 2022	Low	Some concerns	Low	Low	Low	Some concerns
Wang 2013	Some concerns	Some concerns	Low	Low	Low	High
Han 2009	Some concerns	Some concerns	Low	Low	Low	High
Du 2008a	Low	Some concerns	Low	Low	Low	Some concerns
Zhang 2010	Some concerns	Some concerns	Low	Low	Low	High
Du 2008b	Low	Some concerns	Low	Low	Low	Some concerns
Lin 2023	Some concerns	Some concerns	Low	Low	Low	High
Wang 2012	Some concerns	Some concerns	Low	Low	Low	High

Duan 2012	Some concerns	Some concerns	Low	Low	Low	High
He 2001	Some concerns	Some concerns	Low	Low	Low	High
Li 2008	Some concerns	Some concerns	Low	Low	Low	High

Appendix 5: Evaluation of inconsistency and heterogeneity

Clinical outcome	Consistency		Heterogeneity
	**Chi square**	**P value**	**τ2**
Total effective rate	5.34	0.1488	0.000000012
Mayo score	9.52	0.20	0.71
Baron endoscopy score	1.10	0.295	0.343
IL-6	9.38	0.220	0.42
TNF-α	1.24	0.2651	0.248
Recurrence rate	0.18	0.6753	0.0000000324

Abbreviations:IL-6,Interleukin-6. TNF-α,Tumor Necrosis Factor-α.

Appendix 6: Network maps of outcomes

The size of the nodes was proportional to the number of participants included in the trial, and the thickness of lines between the interventions relates to the number of studies for that comparison.

Figure S6.1: Network map of the effect on IL-6.

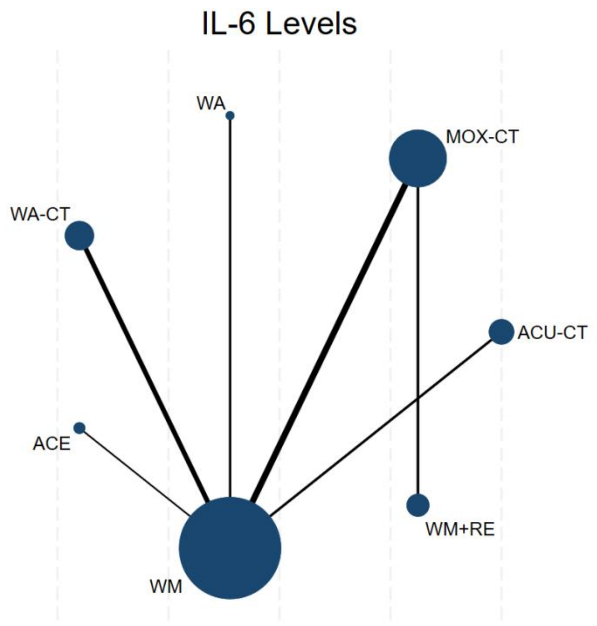

ACU,acupuncture; MOX,moxibustion; WA,warm acupuncture; ACE,acupoint catgut embedding; RE,retention enema; WM,western medicine; CT,combination therapy.

Figure S6.2: Network map of the effect on TNF-α.

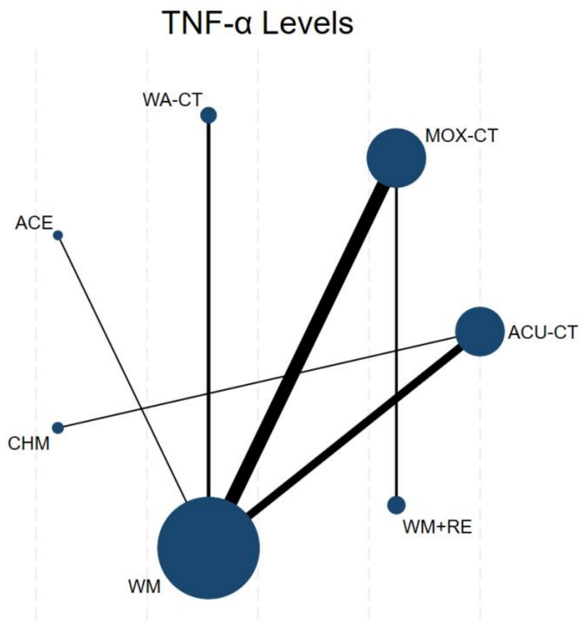

ACU,acupuncture; MOX,moxibustion; WA,warm acupuncture; ACE,acupoint catgut embedding; CHM,Chinese Herbal Medicine; RE,retention enema; WM,western medicine; CT,combination therapy.

Appendix 7: League Tables of Secondary Outcomes.

Table S7.1: IL-6 Levels

ACU-CT						
0.31 (-0.84,1.47)	MOX-CT					
2.24 (0.51,3.98)	1.93 (0.32,3.54)	WA				
0.90 (-0.32,2.12)	0.58 (-0.45,1.62)	-1.35 (-3.00,0.31)	WA-CT			
1.27 (-0.39,2.92)	0.95 (-0.57,2.47)	-0.98 (-2.97,1.02)	0.37 (-1.20,1.94)	ACE		
-0.86 (-1.80,0.07)	-1.18 (-1.85,-0.50)	-3.10 (-4.56,-1.65)	-1.76 (-2.54,-0.98)	-2.13 (-3.49,-0.77)	WM	
-0.61 (-2.10,0.88)	-0.92 (-1.86,0.02)	-2.85 (-4.71,-0.99)	-1.51 (-2.90,-0.11)	-1.88 (-3.66,-0.09)	0.25 (-0.91,1.41)	WM+RE

ACU,acupuncture;MOX,moxibustion;WA,warm acupuncture;ACE,acupoint catgut embedding;RE,retention enema;WM,western medicine;CT,combination therapy. Cells highlighted in blue represent comparisons in which the row intervention is significantly more effective than the column intervention. For instance, -1.18 (-1.85,-0.50) indicates that MOX-CT significantly outperforms WM. Cells highlighted in pink indicate that the row intervention is inferior to the column intervention. For instance, 2.24 (0.51,3.98) indicates that ACU-CT is less effective than WA.

Table S7.2: TNF-α Levels

ACU-CT						
0.37 (-1.47,2.20)	MOX-CT					
0.68 (-1.94,3.31)	0.32 (-2.20,2.83)	WA-CT				
0.14 (-3.29,3.56)	-0.23 (-3.57,3.12)	-0.54 (-4.38,3.29)	ACE			
-2.66 (-5.79,0.46)	-3.03 (-6.65,0.60)	-3.35 (-7.43,0.74)	-2.80 (-7.44,1.84)	CHM		
-1.64 (-3.04,-0.24)	-2.00 (-3.19,-0.81)	-2.32 (-4.54,-0.10)	-1.78 (-4.90,1.35)	1.02 (-2.40,4.45)	WM	
-0.50 (-3.36,2.37)	-0.86 (-3.06,1.34)	-1.18 (-4.52,2.17)	-0.63 (-4.64,3.37)	2.17 (-2.08,6.41)	1.14 (-1.36,3.65)	WM+RE

ACU,acupuncture;MOX,moxibustion;WA,warm acupuncture;ACE,acupoint catgut embedding;CHM,Chinese Herbal Medicine;RE,retention enema;WM,western medicine;CT,combination therapy. Cells highlighted in blue represent comparisons in which the row intervention is significantly more effective than the column intervention. For instance, -1.64 (-3.04,-0.24) indicates that ACU-CT significantly outperforms WM.

Table S7.3: Recurrence Rate

ACU									
0.57 (0.11,2.94)	ACU-CT								
0.56 (0.14,2.21)	0.98 (0.28,3.41)	MOX-CT							
0.69 (0.10,4.92)	1.20 (0.20,7.31)	1.23 (0.23,6.49)	ACE						
0.21 (0.04,1.07)	0.37 (0.09,1.53)	0.38 (0.11,1.30)	0.31 (0.05,1.85)	EA-CT					
0.26 (0.06,1.25)	0.46 (0.12,1.77)	0.47 (0.15,1.48)	0.38 (0.07,2.17)	1.22 (0.33,4.56)	AA+CHM				
0.16 (0.02,1.35)	0.28 (0.07,1.10)	0.29 (0.04,1.83)	0.23 (0.02,2.24)	0.74 (0.10,5.31)	0.61 (0.09,4.16)	CHM			
0.13 (0.02,1.07)	0.23 (0.06,0.84)	0.24 (0.04,1.44)	0.19 (0.02,1.78)	0.62 (0.09,4.18)	0.51 (0.08,3.27)	0.83 (0.13,5.47)	RE		
0.12 (0.03,0.43)	0.21 (0.08,0.57)	0.21 (0.10,0.44)	0.17 (0.04,0.77)	0.56 (0.21,1.47)	0.45 (0.19,1.10)	0.75 (0.14,4.11)	0.89 (0.17,4.61)	WM	
0.15 (0.03,0.65)	0.26 (0.05,1.25)	0.26 (0.09,0.76)	0.21 (0.03,1.47)	0.69 (0.15,3.26)	0.56 (0.13,2.52)	0.92 (0.11,7.50)	1.11 (0.14,8.53)	1.24 (0.37,4.17)	WM+RE

ACU,acupuncture;MOX,moxibustion;ACE,acupoint catgut embedding;EA,Electroacupuncture;AA,auricular acupressure;CHM,Chinese Herbal Medicine;RE,retention enema;WM,western medicine;CT,combination therapy. Cells highlighted in blue represent comparisons in which the row intervention is significantly more effective than the column intervention. For instance, 0.12 (0.03,0.43) indicates that ACU significantly outperforms WM.

Appendix 8: SUCRA and cumulative probability plots

Table S8.1:SUCRA of the effects of acupuncture-related therapies on total effective rate in ulcerative colitis.

Treatment	SUCRA	PrBest	MeanRank
AA+CHM	99.9	98.6	1
ACU	84.8	0.2	3
ACU-CT	47.8	0	7.8
MOX	64.7	0	5.6
MOX-CT	54.1	0	7
WA	75.1	1.1	4.2
WA-CT	46.1	0	8
ACE	42.8	0	8.4
ACE-CT	69.4	0	5
EA-CT	68.7	0	5.1
CHM	16.6	0	11.8
RE	11.9	0	12.4
WM	13.1	0	12.3
WM+RE	5.1	0	13.3

Abbreviations: SUCRA, surface under the cumulative ranking curve.

Table S8.2:SUCRA of the effects of acupuncture-related therapies on Baron endoscopy score in ulcerative colitis.

Treatment	SUCRA	PrBest	MeanRank
ACU-CT	84.1	41.9	1.8
MOX	48.4	15	3.6
MOX-CT	45.4	4.1	3.7
WA	70.4	36	2.5
RE	23.1	3.1	4.8
WM	28.6	0	4.6

Table S8.3:SUCRA of the effects of acupuncture-related therapies on Mayo score in ulcerative colitis.

Treatment	SUCRA	PrBest	MeanRank
ACU-CT	97.5	89.3	1.1
MOX	20.7	0	5
MOX-CT	43.7	0	3.8
WA	68.4	6.2	2.6
WA-CT	65.8	4.4	2.7
WM	3.9	0	5.8

Figure S8.1: Cumulative ranking curve plots of acupuncture-related therapies for IL-6 reduction in ulcerative colitis. Higher surface under the cumulative ranking curve indicates a greater probability of the treatment being the most effective anti-inflammatory option for suppressing IL-6 levels.

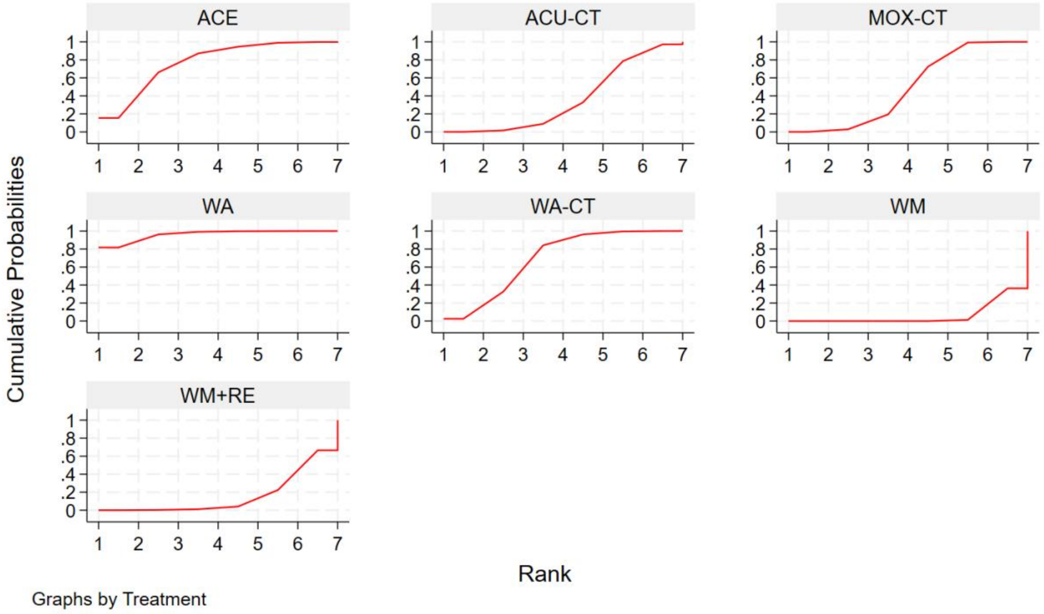

Table S8.4:SUCRA of the effects of acupuncture-related therapies on IL-6 in ulcerative colitis.

Treatment	SUCRA	PrBest	MeanRank
ACU-CT	36.6	0.1	4.8
MOX-CT	49	0.1	4.1
WA	96.1	81.7	1.2
WA-CT	69.2	2.6	2.8
ACE	77	15.5	2.4
WM	6.3	0	6.6
WM+RE	15.8	0	6.1

Figure S8.2: Cumulative ranking curve plots of acupuncture-related therapies for TNF-α reduction in ulcerative colitis. Higher surface under the cumulative ranking curve indicates a greater probability of the treatment being the most effective option for downregulating pro-inflammatory TNF-α expression.

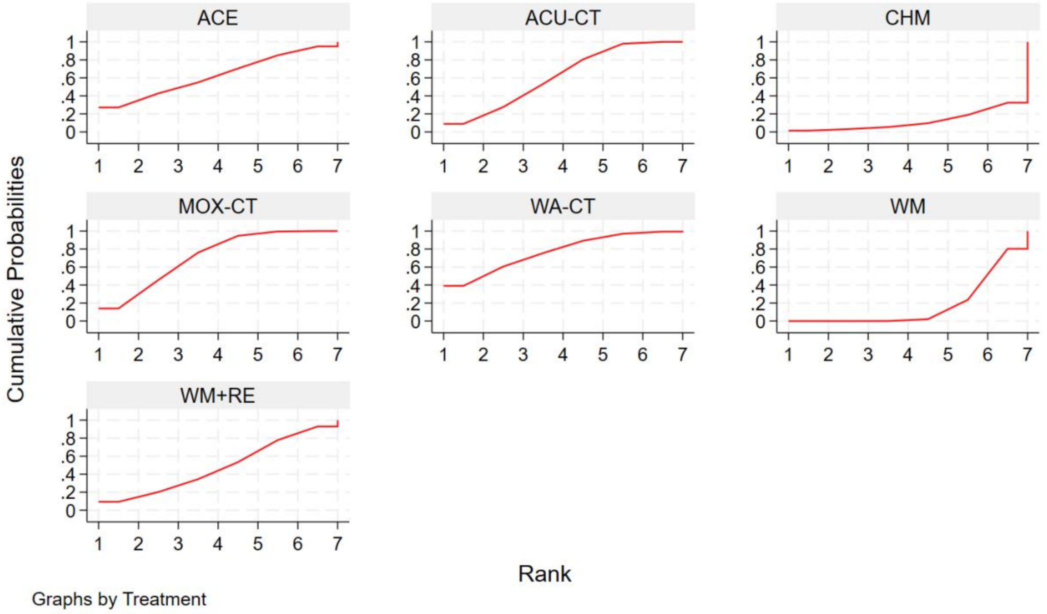

Table S8.5:SUCRA of the effects of acupuncture-related therapies on TNF-α in ulcerative colitis.

Treatment	SUCRA	PrBest	MeanRank
ACU-CT	61.4	8.9	3.3
MOX-CT	71.7	14	2.7
WA-CT	76.8	39.1	2.4
ACE	62.6	27.2	3.2
CHM	11.8	1.4	6.3
WM	17.7	0	5.9
WM+RE	48.1	9.3	4.1

Figure S8.3: Cumulative ranking curve plots of acupuncture-related therapies for reducing recurrence rate in ulcerative colitis. Higher surface under the cumulative ranking curve indicates a greater probability of the treatment being the most effective option for preventing disease relapse.

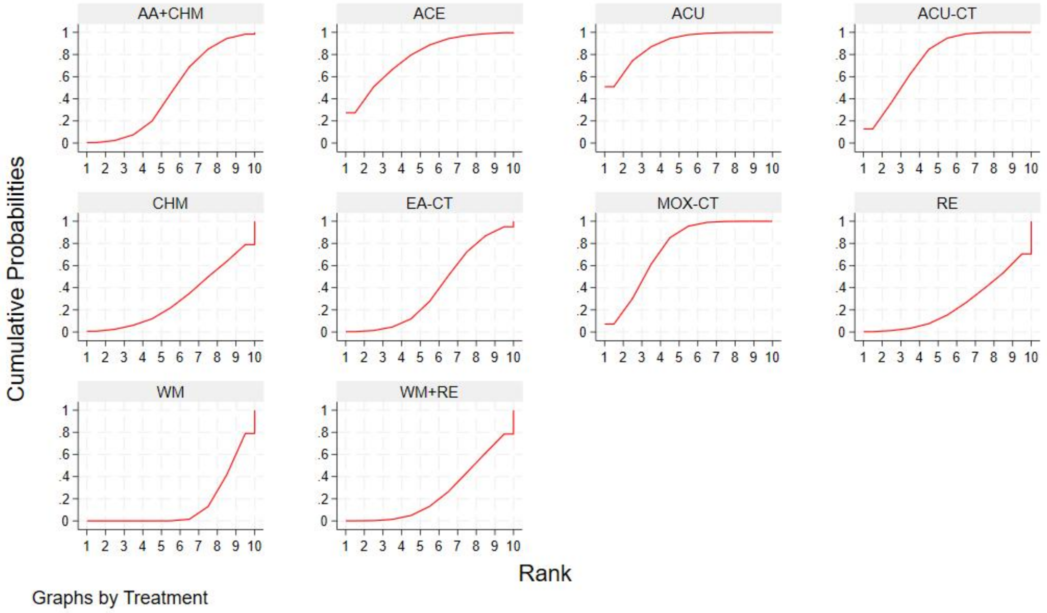

Table S8.6:SUCRA of the effects of acupuncture-related therapies on recurrence rate in ulcerative colitis.

Treatment	SUCRA	PrBest	MeanRank
ACU	89.3	50.9	2
ACU-CT	76.6	12.8	3.1
MOX-CT	75.4	7.1	3.2
ACE	78.1	27.4	3
EA-CT	39	0.3	6.5
AA+CHM	46.9	0.4	5.8
CHM	30	0.7	7.3
RE	24.2	0.3	7.8
WM	15	0	8.7
WM+RE	25.5	0	7.7

Appendix 9: Summary Table of Adverse Events Reported in Included Trials

Study ID	Group	Sample size	Adverse Events
Zhao 2012	ACE+RE	33	NA
	RE	32	nausea, abdominal distension, decreased appetite(n=4)
Zhou 2008	EA+MOX+WM	110	diarrhea(n=4), nausea(n=1), rash(n=1), headache(n=1), leukopenia(n=2)
	WM	110	diarrhea(n=11), nausea(n=3), rash(n=2), leukopenia(n=4)
Yang 2017	MOX	30	anal pain(n=1)
	WM	30	nausea and vomiting(n=1), constipation(n=1)
Zhang 2021	WA+WM	39	nausea(n=1), headache(n=1)
	WM	38	headache(n=1), nausea(n=2), abdominal distension(n=1)
Feng 2020	ACU	36	nausea(n=1), dizziness(n=1)
	WM	36	nausea(n=4), dizziness(n=3), vomiting(n=2)
Gu 2016	MOX+CHM	38	NA
	WM	37	nausea and vomiting(n=3), headache(n=1), skin allergy(n=1), hepatic dysfunction(n=1), leukopenia(n=1)
Shen 2019	MOX+WM	50	gastrointestinal discomfort(n=2), dizziness and headache(n=1)
	WM	50	gastrointestinal discomfort(n=3), dizziness and headache(n=1)
Ye 2024	ACU+CHM	60	abdominal distension(n=1)
	WM	60	dizziness, headaches and abdominal distension(n=2)
Ge 2014	EA+WM	31	nausea(n=1), discomfort(n=1), headache(n=3), leukopenia(n=2), rash(n=2)
	WM	31	nausea(n=3), discomfort(n=1), headache(n=1), leukopenia(n=3)
Zhang 2012	ACU+RE	45	NA
	WM	45	nausea, decreased appetite or itchy skin et al (n=11)
Hui 2012	MOX+RE	55	hemorrhoids (n=5)
	WM	54	nausea, decreased appetite (n=6)
Zhang 2020	WA	34	nausea(n=1), headache(n=1)
	WM	34	headache(n=1), nausea(n=2), abdominal distension(n=1)
Hua 2022	ACU+CHM+WM	59	dizziness(n=2), nausea(n=1)
	WM	59	headache(n=1), dizziness(n=3), nausea(n=2)

Appendix 10: Funnel plots

Figure S10.1: Funnel plot of total effective rate

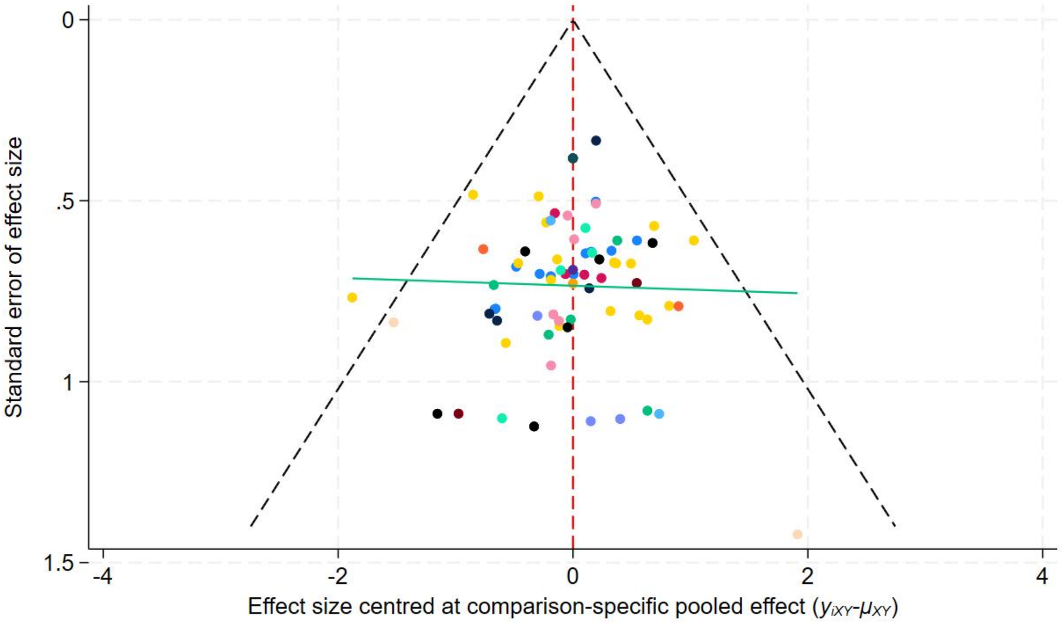

Figure S10.2: Funnel plot of Mayo score

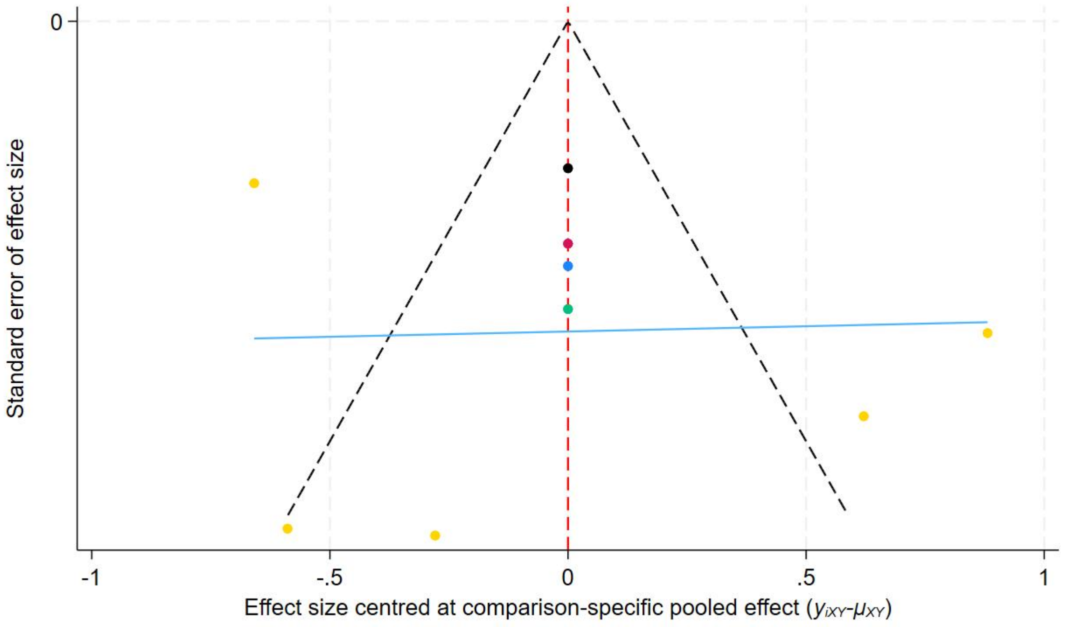

Figure S10.3: Funnel plot of Baron endoscopy score

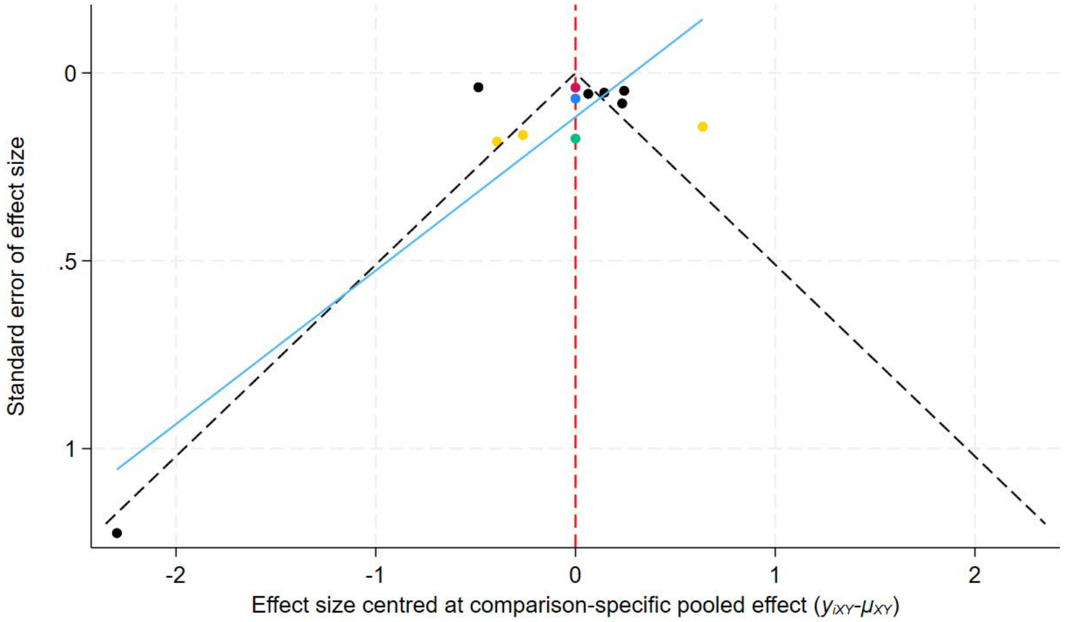

Figure S10.4: Funnel plot of IL-6

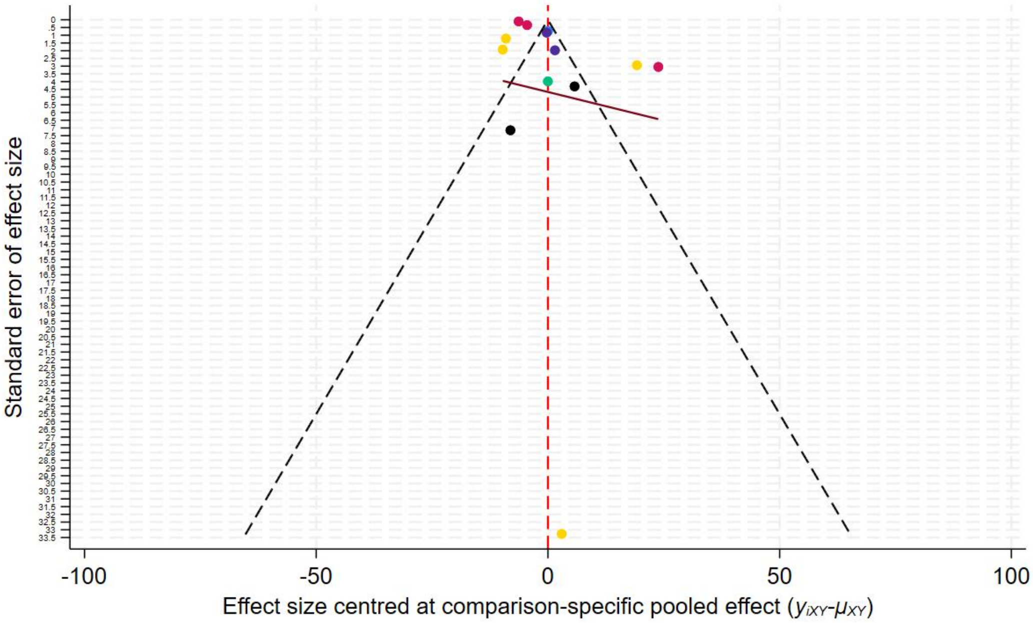

Figure S10.5: Funnel plot of TNF-α

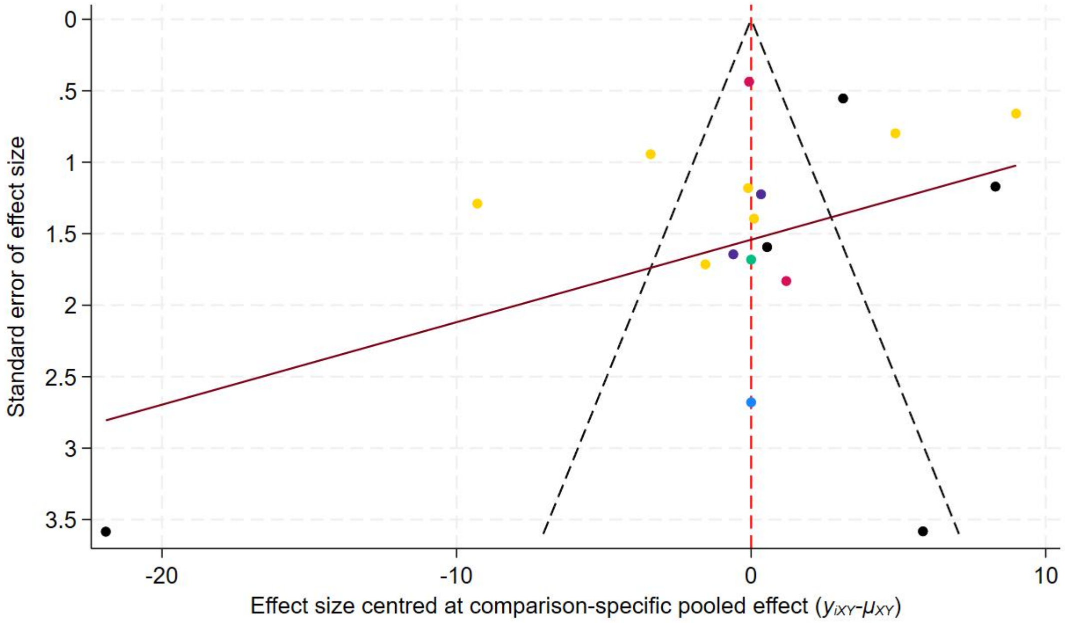

Figure S10.6: Funnel plot of recurrence rate

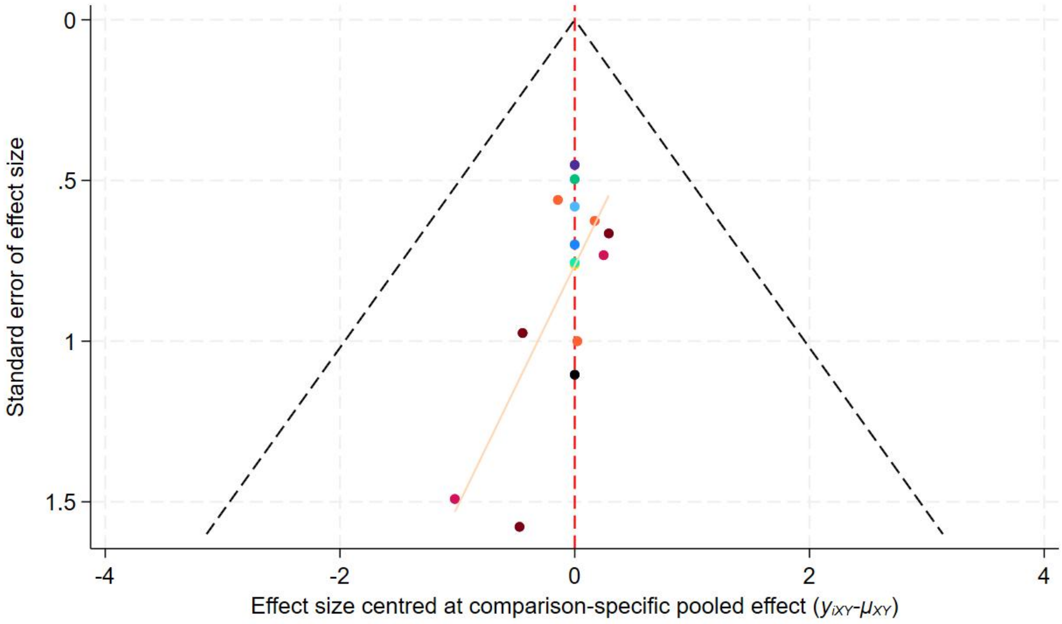

Supplement: Supplementary file 1 [file Data_Sheet_1.pdf]
